# A genome-wide association study reveals a polygenic architecture of speech-in-noise deficits in individuals with self-reported normal hearing

**DOI:** 10.1038/s41598-024-63972-2

**Published:** 2024-06-07

**Authors:** Ishan Sunilkumar Bhatt, Juan Antonio Raygoza Garay, Srividya Grama Bhagavan, Valerie Ingalls, Raquel Dias, Ali Torkamani

**Affiliations:** 1 Department of Communication Sciences and Disorders, University of Iowa, 250 Hawkins Dr, Iowa City, IA 52242 USA; 2 Holden Comprehensive Cancer Center, University of Iowa, Iowa City, IA 52242 USA; 3 Department of Microbiology and Cell Science, University of Florida, Gainesville, FL 32608 USA; 4 Department of Integrative Structural and Computational Biology, Scripps Research Institute, La Jolla, CA 92037 USA

**Keywords:** Speech-in-noise deficits, Hidden hearing loss, Age-related hearing difficulty in noise, Self-reported speech perception, Audiogram, Hearing thresholds, Distortion-product otoacoustic emissions, Extended-high frequency, Genome-wide association, Microtubule-associated protein tau, Glutamate metabotropic receptor 3, Glutamate metabotropic receptor 7, Genetic predisposition to disease, Auditory system, Cochlea, Cortex, Hair cell, Inner ear, Midbrain, Thalamus, Transduction

## Abstract

Speech-in-noise (SIN) perception is a primary complaint of individuals with audiometric hearing loss. SIN performance varies drastically, even among individuals with normal hearing. The present genome-wide association study (GWAS) investigated the genetic basis of SIN deficits in individuals with self-reported normal hearing in quiet situations. GWAS was performed on 279,911 individuals from the UB Biobank cohort, with 58,847 reporting SIN deficits despite reporting normal hearing in quiet. GWAS identified 996 single nucleotide polymorphisms (SNPs), achieving significance (*p* < 5*10^−8^) across four genomic loci. 720 SNPs across 21 loci achieved suggestive significance (*p* < 10^−6^). GWAS signals were enriched in brain tissues, such as the anterior cingulate cortex, dorsolateral prefrontal cortex, entorhinal cortex, frontal cortex, hippocampus, and inferior temporal cortex. Cochlear cell types revealed no significant association with SIN deficits. SIN deficits were associated with various health traits, including neuropsychiatric, sensory, cognitive, metabolic, cardiovascular, and inflammatory conditions. A replication analysis was conducted on 242 healthy young adults. Self-reported speech perception, hearing thresholds (0.25–16 kHz), and distortion product otoacoustic emissions (1–16 kHz) were utilized for the replication analysis. 73 SNPs were replicated with a self-reported speech perception measure. 211 SNPs were replicated with at least one and 66 with at least two audiological measures. 12 SNPs near or within *MAPT*, *GRM3*, and *HLA-DQA1* were replicated for all audiological measures. The present study highlighted a polygenic architecture underlying SIN deficits in individuals with self-reported normal hearing.

## Introduction

From ordering beverages in a noisy café to maintaining conversation at a cocktail party, we process speech-in-noise (SIN) to be efficient at routine tasks. SIN processing is challenging for individuals with audiometric hearing loss (e.g.,^1^). Notably, about 10–15% of individuals report SIN deficits despite normal audiograms^2,3^. Around 10% of patients seeking professional help for communication problems exhibit clinically normal audiograms^4–6^. SIN deficits are associated with an increased risk of dementia, Alzheimer's disease (AD), depressive symptoms, and impaired cognitive functioning^7,8^. Emerging evidence suggests that SIN deficits could be a valuable preclinical indicator of AD-related dementia^9^.

SIN processing requires a dynamic interaction between auditory and cognitive systems involving a series of interdependent biological processes (e.g.,^10^). Sensory cells must process acoustic signals to produce accurate neural codes during SIN processing, helping the higher-order neurons “group” the parts of the signal representing the target and background noise^11–13^. The auditory grouping facilitates unmasking target signals at the cortex^14^. Cognitive factors, such as working memory, selective attention, and language, play a critical role in SIN processing^15,16^. SIN performance varies among individuals with hearing loss and those using hearing aids and cochlear implants^17,18^. SIN performance varies substantially even in individuals with clinically normal hearing thresholds^12^. The biological basis of individual differences in SIN perception remains elusive. We hypothesized that genetic variability could explain individual differences in SIN deficits.

Suprathreshold spectral and temporal auditory processes required for SIN processing exhibit a polygenic inheritance, with heritability estimates ranging from 0.61 to 0.74^19^. Peripheral hearing sensitivity plays a critical role in SIN processing. SIN deficits often accompany age-related hearing loss^18^. A recent genome-wide association study (GWAS) identified several genomic loci associated with age-related hearing difficulty in quiet and noisy situations^20,21^. Genes involved in synaptic functioning, neural processing, inner ear functioning, and cognition were associated with hearing measures. Genes associated with Mendelian inheritance of cochlear hearing loss and those involved in gene regulation showed significant association with age-related hearing difficulty. A meta-analysis of GWAS for age-related hearing impairment identified 21 novel sequence variants^22^. The multi-omics analysis of the GWAS identified several putative genes expressed in mice cochlear tissues^23^. Cognitive traits associated with SIN processing, such as working memory, selective attention, and executive functions, exhibit polygenic inheritance^24^. The above literature indicates a polygenic architecture underlying auditory-cognitive processes involved in SIN deficits.

The present GWAS evaluated the polygenic architecture underlying SIN deficits in individuals with self-reported normal hearing. SIN deficits (i.e., hearing difficulty in noisy situations) and controls (i.e., no hearing difficulty in noisy situations) were defined among individuals reporting no hearing difficulty in quiet. A phenotype definition plays a central role in identifying the genetic architecture of complex traits. The phenotype of self-reported hearing difficulty in quiet could be highly influenced by audiometric hearing loss (e.g.,^25^). Excluding individuals with hearing difficulty in quiet could help uncover the genomic signals involved in suprathreshold auditory-cognitive processes. In addition, we conduct GWAS-based functional enrichment analysis to further understand the genomic processes underlying SIN deficits. We performed a replication analysis on 242 young adults with self-reported normal hearing. Speech performance was evaluated using the Speech, Spatial, and Quality of Hearing scale (12-item version)^26^. Subclinical differences in sensory functioning could contribute to SIN deficits in youth with self-reported normal hearing^27^. We utilized two clinical measures—puretone hearing thresholds (HTs) and distortion-product otoacoustic emissions (DPOAEs) to study the influence of genetic variants associated with SIN deficits on the cochlear processing of young adults. We reasoned that genetic variants involved in SIN deficits could explain individual differences in HTs and DPOAEs if they have subclinical effects on cochlear functioning.

## Methods

The project was approved by the UK Biobank (ID: 68779). The study was conducted according to the UK Biobank guidelines and regulations. Participant recruitment, informed consent, and data collection were conducted according to the UK Biobank guidelines and regulations. The UK Biobank database with demographic, questionnaire, and genome-wide single nucleotide polymorphism (SNP) markers were obtained. The database contains demographic and questionnaire data from 502,415 participants. The blood-derived DNA samples were used to obtain SNP genotypes across the genome. The methodological details of the blood sample collection procedure and questionnaire-based data collection are described earlier^28^. The University of Iowa institutional review board approved the UK Biobank data handling and analysis procedures (IRB: 202103221). The replication sample was collected at the University of Iowa campus. Informed consent was obtained from all participants. All procedures used for the replication analysis were approved by the University of Iowa Institutional Review Board (IRB: 202010165).

### Phenotype definition: SIN deficits

Participants responded to a hearing-health outcomes questionnaire at the UK Biobank assessment center. The questionnaire investigated SIN deficits with the following question: Data-field 2257, “Do you find it difficult to follow a conversation if there is background noise (such as TV, radio, children playing)?”. The answer choices included “Yes”, “No”, “Do not know”, and “Prefer not to answer”. The questionnaire inquired about hearing loss with the following question: Data-field 2247, “Do you have any difficulty with your hearing?”. The answer choices included “Yes,” “No,” “I am completely deaf,” “Do not know,” and “Prefer not to answer.” These questions were answered in multiple instances by some participants (Supplementary file S1). Participants reporting “Yes” for any instances were identified, and derived hearing difficulty and hearing difficulty in noise variables were calculated (Supplementary file S1—Supplementary Table 1 and Supplementary Table 2). The SIN deficits *phenotype* was obtained by identifying cases reporting “Yes” to hearing difficulty in background noise without self-reported hearing loss and controls reporting “No” to hearing difficulty in background noise and hearing difficulty in quiet. The questionnaire responses for demographic factors, such as age, sex, and ethnicity, were extracted.

### Genome-wide association study (GWAS)

The genotyping was conducted using two platforms: Affymetrix UK BiLEVE Axiom (N ~ 50,000 samples) and Affymetrix UK Biobank Axiom array (N ~ 450,000 samples). The genotypes were augmented using the imputation pipeline using the Haplotype Reference Consortium^29^. Genetic variants with high heterozygosity or missingness were excluded. Individuals reporting White British and Irish ancestry based on the genomic principal component were included. We identified 343,104 participants with valid responses to hearing difficulty (Data field: 2247) and hearing difficulty in noisy situations (Data field: 2257). 33,118 participants reporting non-British and Irish ethnicity were excluded. The kinship coefficients were calculated, and related individuals were filtered out by excluding one individual in each pair of related individuals with a kinship coefficient of > 0.0844 (i.e., > third-degree relatives), excluding 30,075 participants. The GWAS analysis was conducted on 279,911 participants (58,844 cases and 221,067 controls).

The imputed genotype database was subjected to filters: a minor allele frequency of > 0.005, a genotyping rate of > 99%, a minor allele count of > 890, and a Hardy–Weinberg equilibrium test *p* < 10^−15^. GWAS was conducted using REGENIE, which employs computationally efficient machine-learning algorithms for genome-wide association analysis^30^. The analysis was conducted using logistic regression under an additive genetic model. The genetic variants in low-complexity regions and inter-chromosome LD were removed using “—exclude” flag in REGENIE Step 1. LD pruning was applied to 471,734 directly genotyped SNPs (R^2^ = 0.9, window size = 1000, step size = 100), and Step 1 was conducted using a batch size of 1000. REGENIE Step 2 was conducted on 8,741,958 imputed genetic variants. The block size of 400, an approximation of Firth correction for p values < 0.01 was applied using “—firth” and “—approx.” flags. The following covariates were used in REGENIE Steps 1 and 2: age, age^2^, sex, age*sex, ethnicity, genetic batch, testing site, and the first 10 genomic principal components. The *p*-value threshold 5*10^–8^ was used to identify SNPs showing significant association with SIN deficits.

### Functional enrichment analysis, heritability estimates, and genomic correlations

Functional enrichment analysis was conducted with FUMA^31^, using the following settings: maximum *p*-value of lead SNPs of 10^−5^, maximum p-value cutoff of 10^−4^, r^2^ threshold to define independent significant SNPs of ≥ 0.8, 2nd r^2^ threshold to define lead SNPs of ≥ 0.1, reference panel population of UKB release2b 10k White British, maximum distance between LD blocks to merge into a locus of < 250 kb, distance to genes or functional consequences of SNPs on genes to map of 100 kb. The expressive quantitative trait loci (eQTL) mapping was conducted for brain tissues using BRAINEAC database using the following settings: eQTL FDR *p*-value threshold of < 0.05, and additional annotations were performed using Brain Open Chromatin Atlas. The gene mapping with 3D chromatin interaction mapping was conducted using chromatin interaction data of the adult cortex, and additional annotations were taken from Brain Open Chromatin Atlas. The gene-based analysis was conducted with MAGMA within FUMA using a window size of 50 kb upstream and 40 kb downstream. All other values were set to default. Linkage-disequilibrium score (LDSC) regression was conducted on the GWAS summary statistics to calculate SNP-based heritability estimates and genomic inflation factor (Lamda genomic control (GC))^32,33^. The GWAS summary statistics for SIN deficits were processed by the Complex Trait Genetics Virtual Lab (https://vl.genoma.io/), and cross-trait genomic correlation with LDSC was performed with available traits^34^.

### Cochlear cell line enrichment analysis

Cochlear cell lines likely involved in SIN deficits are not included in FUMA-based enrichment analysis. We performed cochlear cell-line enrichment analysis using single-cell transcriptomic profiles obtained from mice cochlear tissues^35^. The transcriptomic profiles were obtained at 10 months of age from 48 Carworth Farms White mice. The auditory phenotyping was conducted using auditory brainstem responses (ABR), and mice were categorized based on their hearing status, ranging from normal hearing to profound hearing loss. The transcriptomics-integrated cell-line enrichment analysis was conducted using the methods described elsewhere^36^. In brief, the transcriptomic databases were downloaded from gEAR (https://umgear.org/)^37^. Data quality was assessed using the number of nuclei per sample, the number of unique molecular identifiers (UMIs), the number of genes detected, and the number of mitochondrial genes per nuclei. Nuclei were removed if they had fewer than 1,000 UMIs and/or fewer than 500 detected genes. Nuclei that had the number of detected mitochondrial genes were removed. The databases were processed in RStudio with the Seurat package^38^. The following filters were applied: RNA count ranging from 1000 to 30,000; gene and non-coding RNA count ranging from 500 to 5000; percentage of mitochondrial genes < 2%. The filtering procedure resulted in 176,327 cells from 48 mice. The cell type-specific pseudobulk average gene expression levels were calculated and were normalized (log2). Mouse ensembl IDs were converted into human gene entrez IDs using a homolog map^39^. Genes with duplicate entrez IDs were excluded. The gene-level association statistics (Z-score) derived from MAGMA gene-set analyses were predicted from gene expression (transcriptomic data in mice) while controlling for gene length, linkage disequilibrium, and average gene expression across all cell types. The enrichment analysis was conducted in MAGMA using the –gene-covar flag^40^.

### Replication sample—audiological data collection

A sample of 242 young adults (81 males and 161 females) aged 18–35 years with self-reported normal hearing was recruited. All data collection procedures were conducted in a sound-treated booth meeting the ANSI standards. The otoscopic exam and immittance audiometry using Titan IMP440 (Interacostics, Middelfart, Denmark) were performed on all participants. Participants with normal otoscopic findings and type A tympanograms were tested further. The participants completed a questionnaire inquiring about age, sex, ethnicity, and history of health conditions. Self-reported SIN skills were assessed through the Speech, Spatial, and Quality of Hearing scale (SSQ12)^26^. SSQ12 includes 12 questions investigating the quality of speech perception in the following domains: speech-in-noise, multiple speech streams, localization distance and movement, listening effort, segregation, identification of sound, quality, and naturalness. The responses were elicited using a visual analog scale of 0–10, with high values indicating better speech perception abilities. SSQ12 evaluates the dimensions of speech perception in real life that could remain unassessed from performance-based SIN tests. SSQ12 served as the primary SIN measure for the replication analysis. We reasoned that healthy young adults carrying the risk alleles of genetic variants associated with SIN deficits in the GWAS would exhibit subclinical difficulties in speech perception in challenging listening conditions.

HTs were measured using AVANT Stealth Clinical Audiometer (MedRx Inc., Largo, FL) at 0.25, 0.5, 1, 2, 3, 4, 6, and 8 kHz with insert receivers ER-3A. Extended high-frequency HTs were measured at 9, 10, 11.2, 12.5, 14, and 16 kHz using circumaural DD450 headphones. DPOAEs were measured using a Mimosa HearID system (Mimosa Acoustics, Champaign, IL) connected to ER-10C (Etymotic Research, Elk Grove Village, IL). The DPOAE recording apparatus was calibrated following the manufacturer’s guidelines. DPOAEs were obtained at 2f1-f2 for f2 at 1, 2, 3, 4, 6, 8, 9, 10, 11.2, 12.5, 14, and 16 k Hz for a stimulus frequency ratio f2/f1 of 1.22 and a primary tones combination of 65/55 dB SPL. The recording was stopped until one of the following stopping logics was achieved: a signal-to-noise ratio (SNR) > 12 dB, a noise floor of ≤ 20 dB SPL, or a maximum signal duration of > 10 s. The data processing and missing data handling were conducted following the procedures described elsewhere^41^.

### Replication sample—genotyping

Saliva samples were collected from all participants with the Oragene OGR-600 DISCOVER kit (DNA Genotek, Ottawa, Canada). The saliva samples were stored at room temperature for < 1 year. Salivary DNA samples were extracted (PrepITL2P, DNA Genotek, Ottawa, Canada). DNA was fragmented, and fragmented DNA molecules were selected. The selected DNA fragments were subjected to end-repair, amplification, and clean-up. The double-stranded polymerase chain reaction products were heat denatured, single-stranded circle DNAs were obtained, and the library was quality controlled. Low-pass whole genome sequencing was conducted with DNBSEQ-G400. A genomic imputation pipeline obtained genotypes for > 10 million markers^42^.

### Replication sample—statistical analyses

A linear mixed model (LMM) was used to evaluate the effect of selected SNPs (*p* < 10^−6^ in GWAS) on SSQ12, HTs, and DPOAEs while controlling for the effects of between-subject factors of age, sex, and self-reported ethnicity (European/Non-European). Plink 2.0 (Cog-genomics.org) was used to run the principal component analysis on the genomic data. The first ten genomic principal components (PCs) were used as covariates to control for the genomic ethnicity. The following models were fit using lme4 package in RStudio to investigate the main effect of selected SNPs on SSQ12, HTs, and DPOAEs^43^.

$$SSQ12=Intercept+Age+Sex+Ethnicity+PC1\dots +PC10+(1|Question\#)+SNPi$$

$$HTs=Intercept+ Age+ Sex+ Ethnicity+PC1\dots +PC10+(1|RandomEffect)+SNPi$$

$$DPOAEs=Intercept+Age+Sex+Ethnicity+PC1\dots +PC10+(1|RandomEffect)+SNPi$$

Here, *i* is a vector of selected SNPs (*p* < 10^−6^ in GWAS). LMM was used to control random effects (i.e., question number for SSQ12 and frequency and ear for HTs and DPOAEs) and fixed effects (age, sex, ethnicity). Frequency (1–16 kHz) and ear (right and left) were nested to create a combined random effect variable. SNP-specific beta and *p*-values were derived from SSQ12, HTs, and DPOAEs. The *p*-value adjustment was performed while accounting for the linkage disequilibrium using the methods described elsewhere^44^. Briefly, we computed the correlation matrix of the genotype dosage data in poolR (RStudio). The method estimates the number of independent tests based on the eigenvalues of the correlation matrix. The effective *p*-value threshold was set to 0.05/(number of effective tests).

### SNP-specific replication score

SNPs achieving the adjusted *p* < 0.05 and the beta values showing a consistent direction of association with GWAS were identified. The SIN deficits phenotype was coded binary (0-control, 1-case) for GWAS. Lower values of SSQ12 indicate poorer physiology; hence, SNPs achieving positive beta values for GWAS should reveal negative beta values for SSQ12 and vice versa. A replication score of “1” was assigned to SNPs if replicated for SSQ12. Higher values of HTs indicate poorer physiology. SNPs achieving positive beta values for GWAS should reveal positive beta values for HTs and vice versa. A replication score of “1” was assigned to SNPs if replicated for HTs. Lower values of DPOAEs indicate poorer physiology. Hence, SNPs achieving positive beta values for GWAS should reveal negative beta values for DPOAEs and vice versa. A replication score of “1” was assigned to SNPs if replicated for DPOAEs. A replication score was calculated for each SNP, ranging from 0 to 3. A score of 3 indicates replication with SSQ12, HTs, and DPOAEs. SSQ12 was the primary metric for replication analysis, but it might not be sensitive enough to detect subclinical changes in sensory functions. HTs and DPOAEs, on the other hand, might be more sensitive for detecting subclinical changes in sensory functions. However, they are not sensitive enough to identify minor changes in suprathreshold auditory-cognitive processes underlying SIN deficits^45^. Therefore, the replication analysis followed a test battery approach, and the results should be interpreted under this framework, considering the relative strengths and weaknesses of the replication measures.

## Results

Table 1 presents the cross table for SIN deficits derived from hearing difficulty (Data field: 2247) and hearing difficulty in noise (Data field: 2257). 72,690 participants reported hearing difficulty in noisy situations without reporting hearing difficulty in quiet. Table 2 presents cross-table analysis and Chi-square statistics showing that age (older > younger), sex (male > female), and ethnicity (Irish > British) were significant predictors of SIN deficits in the sample included for GWAS.

**Table 1:** Speech-in-noise (SIN) deficits derived from hearing difficulty in noise and hearing difficulty variables.

		Derived hearing difficulty (data field: 2257)		Total
		Yes	No	
Derived hearing difficulty in noise (data field: 2247)	Yes	110,818	72,690 (Cases)	183,508
	No	19,260	270,414 (Controls)	289,674
Total		130,078	343,104	473,182

SIN phenotype was derived by identifying a group of participants (cases) reporting hearing difficulty in noise without hearing difficulty and a group of participants (controls) reporting no hearing difficulty in noise without hearing difficulty.

**Table 2:** Cross-tab analysis between speech-in-noise (SIN) deficits and demographic factors (N = 279,911).

		SIN deficits			
		No	Yes		
		Count (Layer %)	Count (Layer %)	Odds ratio	*p*-value
Sex	Female	130,813 (46.7%)	28,871 (10.3%)	290.2	< 10^−50^
	Male	90,254 (32.2%)	29,973 (10.7%)		
Age	< = 50 years	62,594 (22.4%)	14,694 (5.2%)	1938.5	< 10^−50^
	51–60 years	79,578 (28.4%)	21,524 (7.7%)		
	61+ years	78,895 (28.2%)	22,626 (8.1%)		
Ethnicity	British	214,871 (76.8%)	57,042 (20.4%)	11.2	< 0.001
	Irish	6196 (2.2%)	1802 (0.6%)		

### GWAS results for SIN deficits

**Figure 1:**
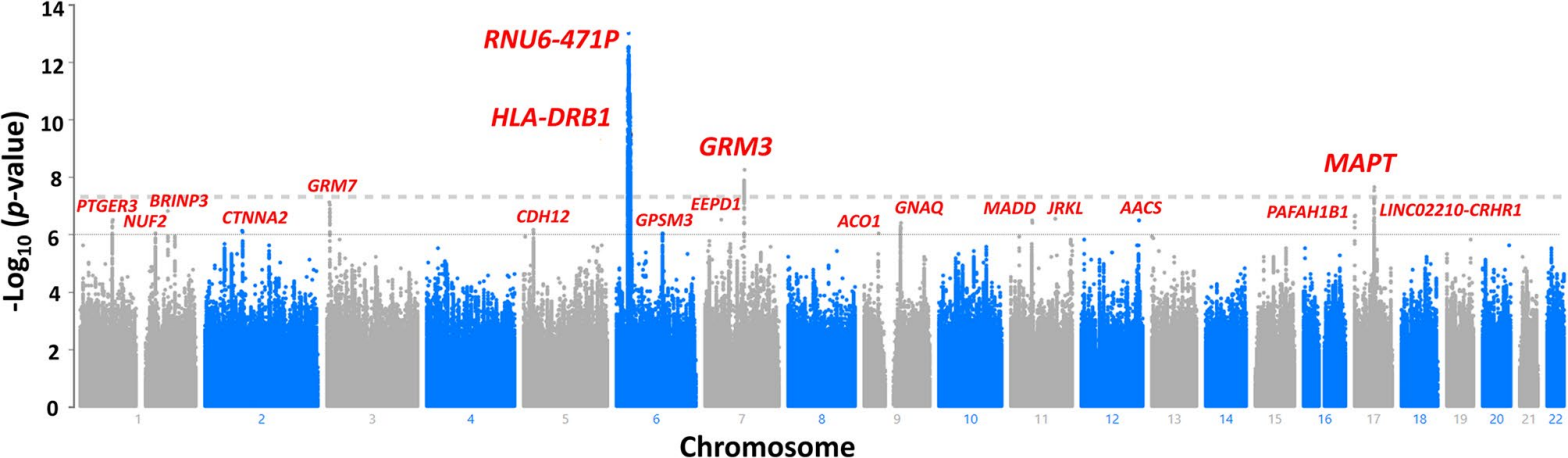
Manhattan plot of the genome-wide association study on SIN deficits in individuals with self-reported normal hearing. The gray dashed line presents the genome-wide significance threshold (*p* < 5E−8), and the dashed black line indicates the threshold of suggestive significance (*p* < E−6).

**Figure 2:**
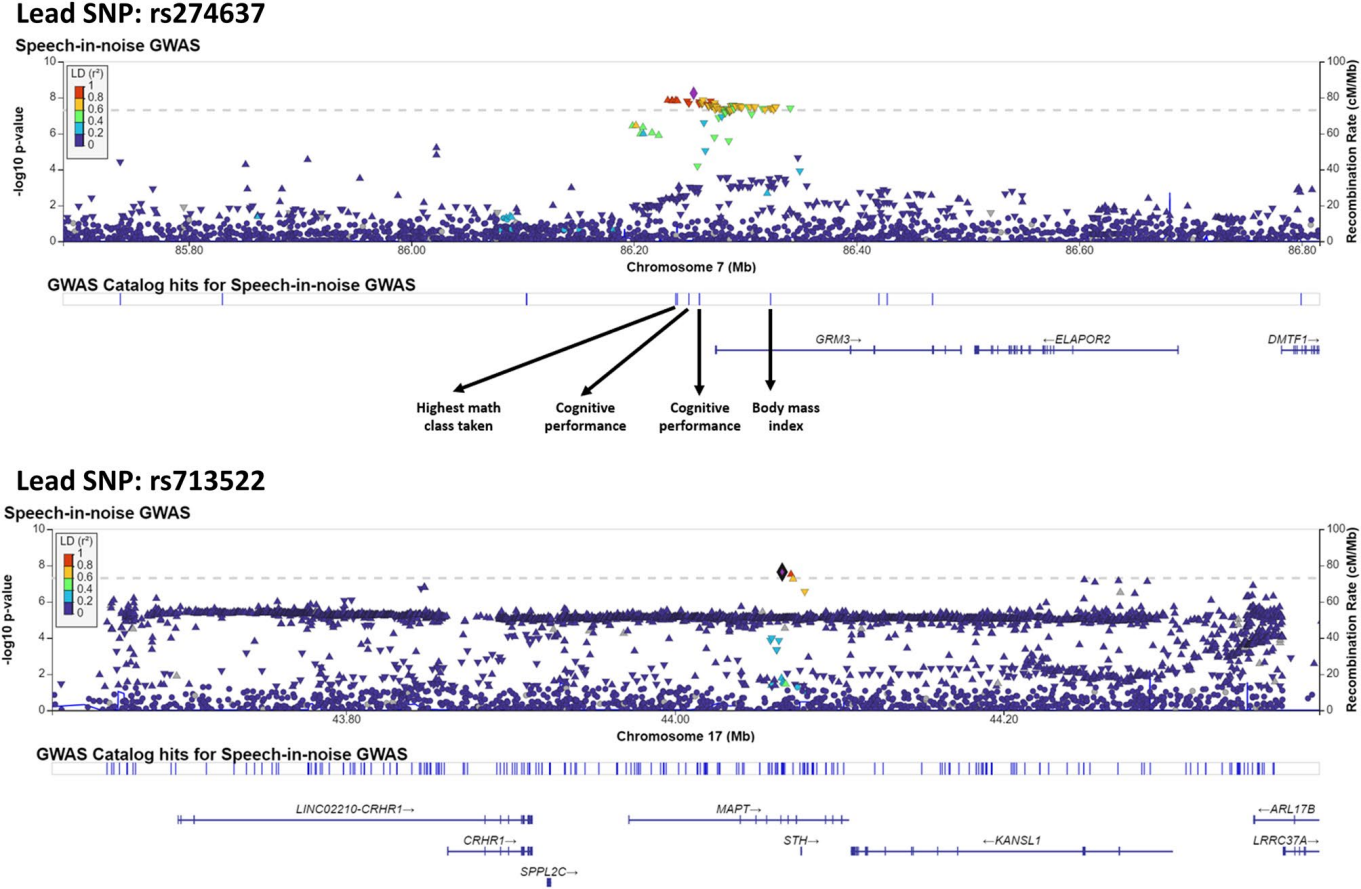
LocusZoom plots of the genomic loci near *GRM3* (top panel) and *MAPT* (bottom panel). The color of the dots indicates the degree of linkage disequilibrium. The blue line presents the recombination rate.

The results of GWAS are shown as a Manhattan plot in Fig. 1 (GC lambda = 1.155), Linkage disequilibrium score regression intercept (SE)=1.003 (0.0077). Table 3 presents the statistical summary of the lead SNPs for genomic loci achieving genome-wide significance at *p* < 5*10^−8^ and suggestive significance at *p* < 10^−6^. The GWAS identified four independent loci achieving GWAS significance (996 SNPs) and 21 loci achieving suggestive significance (720 SNPs). Among four loci achieving GWAS significance, 2 were on the major histocompatibility complex (MHC) region on chromosome 6. Table 3 provides GWAS summary statistics of the lead SNPs for loci achieving GWAS significance and suggestive significance. Figure 2 provides LocusZoom plots for the loci near *MAPT* and *GRM3,* achieving the GWAS significance. Supplementary file S1 presents the LocusZoom plots for loci achieving suggestive significance. SNPs within *GRM3* and *MAPT* showed significant association with SIN deficits.

**Table 3:** GWAS summary statistics of the lead SNPs for the loci associated with SIN deficits.

CHR	BP	SNP	A0	A1^Ref^	A1 Freq	Beta	SE	*p*-value	Replication
6	27,556,141	rs13201294	A	T	0.131	− 0.074	0.009	9.90E−14*	*CD83P1, RNU6-471P*
6	32,571,350	rs111631417	G	C	0.376	− 0.045	0.007	7.20E−10*	*HLA-DQA1, HLA-DRB1*
7	86,253,638	rs274637	C	T	0.642	− 0.040	0.006	5.71E−09*	*GRM3*
17	44,065,263	rs713522	T	C	0.420	0.0376	0.006	2.30E−08*	*MAPT*
3	6,189,009	rs116208485	T	C	0.139	− 0.051	0.009	7.74E−08	*GRM7*
6	32,161,366	rs204991	T	C	0.221	− 0.042	0.008	1.45E−07	*RNF5*
1	190,291,562	rs147935374	G	A	0.021	− 0.123	0.023	1.56E−07	*BRINP3*
17	43,847,569	rs570010559	TTT…	T	0.267	0.039	0.007	1.61E−07	*LINC02210-CRHR1*
17	2,555,592	rs7219015	C	T	0.215	0.041	0.008	2.27E−07	*PAFAH1B1*
11	97,179,965	rs146385063	G	A	0.010	0.165	0.031	2.90E−07	*JRKL*
7	36,189,045	rs117021143	T	C	0.022	0.121	0.023	3.13E−07	*EEPD1*
1	71,725,353	rs537548369	G	GTA…	0.443	− 0.034	0.006	3.18E−07	*NEGR1*
12	125,630,997	rs34615808	C	A	0.105	0.055	0.010	3.31E−07	*AACS*, *TMEM132B*
11	47,306,630	rs35233100	C	T	0.062	0.069	0.013	3.32E−07	*MADD*
17	42,779,633	rs138093101	G	GGC…	0.505	0.034	0.006	3.41E−07	DBF4B, CCDC43
11	47,828,404	rs3837374	C	T	0.507	− 0.038	0.007	4.03E−07	*NUP160*
9	80,661,256	rs11357406	TC	T	0.687	0.036	0.007	4.07E−07	*GNAQ*
6	25,191,039	rs76671369	G	C	0.087	− 0.059	0.011	4.75E−07	*CARMIL1*
9	80,319,769	rs34239497	C	CTT	0.724	0.037	0.007	6.33E−07	*GNAQ*, *GNA14*
5	22,309,022		AGA…	A	0.573	0.034	0.006	7.02E−07	*CDH12*
2	81,408,132	rs17020020	A	G	0.064	0.066	0.013	7.56E−07	*CTNNA2*
9	32,428,841	rs112618859	C	CA	0.188	− 0.043	0.008	9.37E−07	*ACO1*
1	163,763,429		GGT	G	0.493	− 0.034	0.006	9.44E−07	*NUF2*, *RGS5*
6	100,929,516	rs6903206	C	T	0.510	0.032	0.006	9.62E−07	*SIM1*
6	101,191,397	rs1039033	C	T	0.527	0.032	0.006	9.89E−07	*ASSC3*

*p < 5E−08.

### FUMA enrichment analysis for SIN deficits

The results of the enrichment analysis are presented in Supplementary file S2. FUMA-based MAGMA analysis revealed significant enrichment of brain tissue and G-protein signaling through tubby proteins. The enrichment analysis using curated databases identified about 100 gene sets, such as deacetylate histones, DNA methylation, activated PKN1 stimulates transcription of androgen receptor-regulated *KLK2* and *KLK3* genes, oxidative stress-induced senescence, epigenetic regulation of gene expression, notch signaling, neurotransmitter receptors and postsynaptic signal transmission, transmission across chemical synapses, meiotic synapses, and GABA receptor activation. Systemic Lupus erythematosus, melanogenesis, and pyruvate metabolism revealed significant enrichment. About 52 biological processes showed significant enrichment with SIN deficits, including chromatin assembly and disassembly, nucleosome organization, DNA packing, protein-DNA complex subunit organization, DNA conformational change, gene silencing, G protein-coupled receptor signaling pathway, and sialic acid transport. Four WikiPathways revealed significant enrichment, including histone modifications, effects of progeria on the involved genes in Hutchinson-Gilford Progeria syndrome, *FBXL10* enhancement of MAP/ERK singling in diffuse large B-cell lymphoma, and genotoxicity.

**Figure 3:**
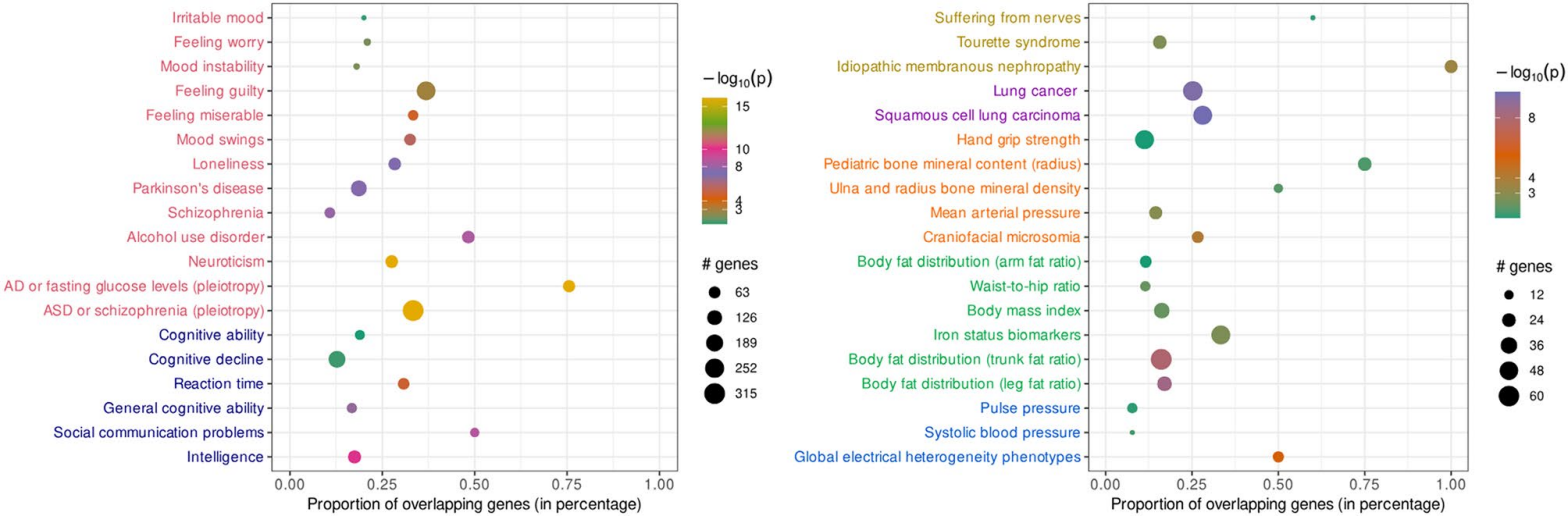
Results of the GWAS gene set enrichment analysis with FUMA. This figure presents a subset of the GWAS gene set associations obtained for the SIN phenotype (more details in Supplementary file S2). The phenotype category is delineated with distinct colors: Red—Neuropsychiatric; Navy Blue—Cognitive; Yellow—Neuropathy; Purple—Neoplasm; Orange—Musculoskeletal; Green—Metabolic; Blue—Cardiovascular. The X-axis represents the proportion of overlapping genes, and their colors represent the enrichment *p*-values. The circle size is determined by the number of genes tested for association.

Seventy-two GWAS catalog gene sets showed significant enrichment with SIN deficits (Fig. 3, more details in Supplementary file S2). Neuropsychiatric traits, such as autism spectrum disorder, schizophrenia, AD, neuroticism, Parkinson's disease, loneliness, feeling guilty, mood instability, and response to cognitive-behavioral therapy in major depressive disorder, showed significant association with SIN deficits. Cognitive traits, such as intelligence, general cognitive ability, reaction time, cognitive decline rate in late mild cognitive impairment, and social communication problems, showed significantly overlapping gene sets with SIN deficits. Metabolic traits, including body fat distribution, body mass index, trunk and leg fat distribution, and iron status, showed significant enrichment with SIN deficits. Sensory phenotypes, such as intraocular pressure, sense of smell, and myopia, showed significant association with SIN deficits. Sleep-related traits, such as daytime sleepiness, sleep duration, and insomnia symptoms, showed significant association with SIN deficits. Cardiovascular, inflammatory, musculoskeletal, and other miscellaneous revealed significant associations with SIN deficits. Similar results were obtained with genomic correlations, with 66 traits showing significant associations with SIN deficits (Supplementary file S2). The top results included neuropsychiatric traits, such as worrying too long after embarrassment, guilt feeling, neuroticism score, depression, isolation, irritability, and fed-up feelings.

### Partitioned heritability-based enrichment analysis

**Figure 4:**
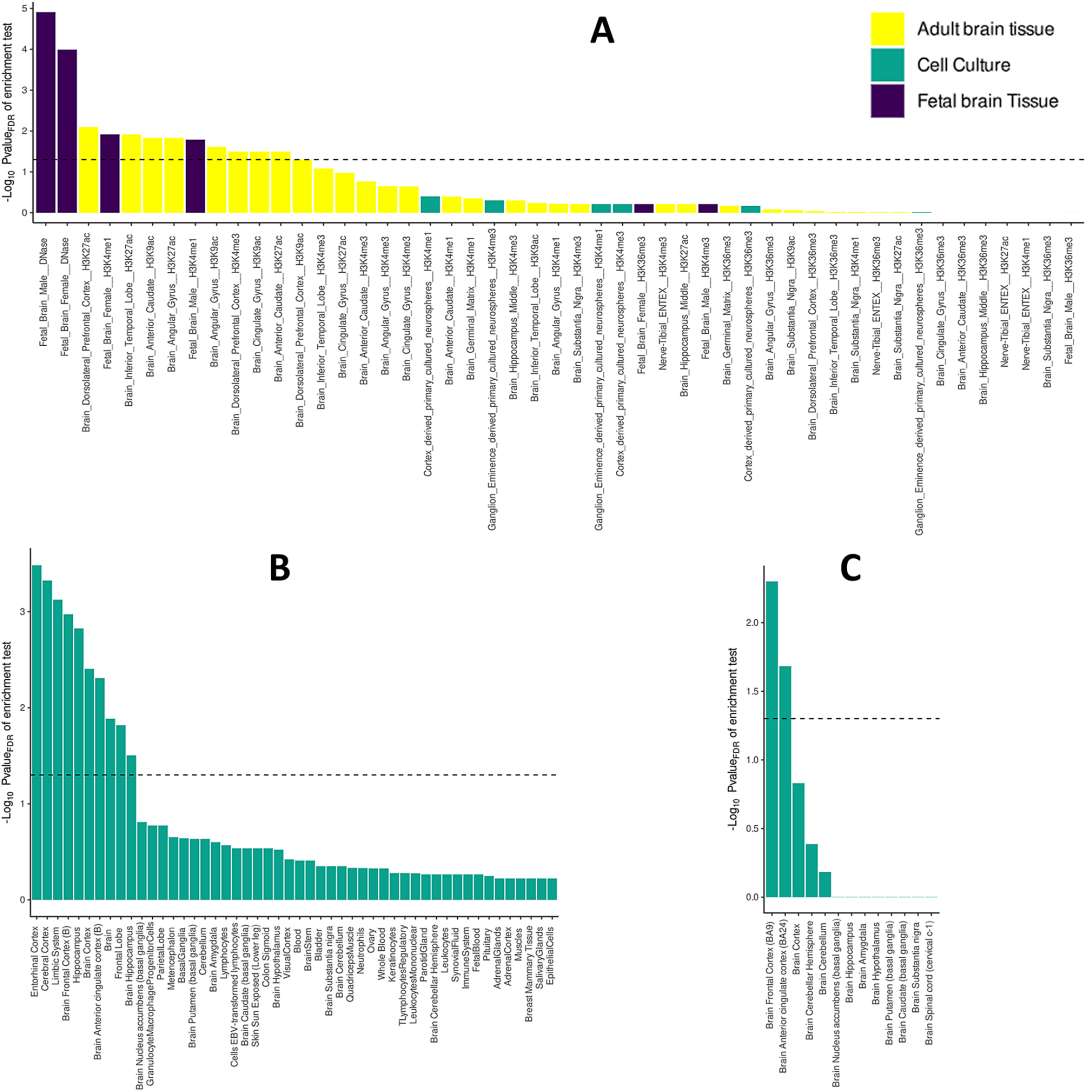
Cell- and tissue-specific partitioning heritability analysis of SIN deficits. (**A**) Partitioned heritability with LDSC regression shows enrichment in brain-specific regulatory regions of the genome. Tissue-specific regulatory elements are marked by histone 3 acetylation or DNase hypersensitivity (for open chromatin) and H3K4me1 (for enhancers). (**B**) Partitioned heritability-based enrichment analysis identified tissues involved in SIN deficits. (**C**) Brain-specific analysis of partitioned heritability shows distinct regions of the brain associated with SIN genetic architecture. The graph shows *p*-values (in − log10) of tissue and cell marker types. The dashed line shows the *p*-value threshold for significant enrichment after FDR correction for the number of gene sets tested.

SNP-based heritability was calculated with the LDSC regression (h^2^(SE) = 0.039 (0.0026), Lambda GC = 1.20, intercept (SE) = 1.003 (0.0077), mean Chi^2^ = 1.229). The regression intercept was 1.003, indicating that the inflation in Lambda GC was due to polygenic signals related to SIN deficits, and there was no indication of residual population stratification in GWAS. Partitioned heritability-based enrichment analysis revealed the enrichment of the GWAS signals into the frontal cortex and anterior cingulate cortex. Brain-tissue-specific gene expression-based enrichment analysis revealed that the GWAS signals were enriched in the entorhinal cortex, cerebral cortex, limbic system, frontal cortex, hippocampus, and anterior cingulate cortex. Cell-line enrichment analysis identified the dorsolateral prefrontal cortex (H3K27ac, H3K4me3, H3Kac), inferior temporal lobe (H3K27ac), anterior caudate (H3K9ac, H3K4me3), and angular gyrus (H3K9ac, H3K4me3) (Fig. 4).

### Results of the cochlear cell-line enrichment analysis

**Figure 5:**
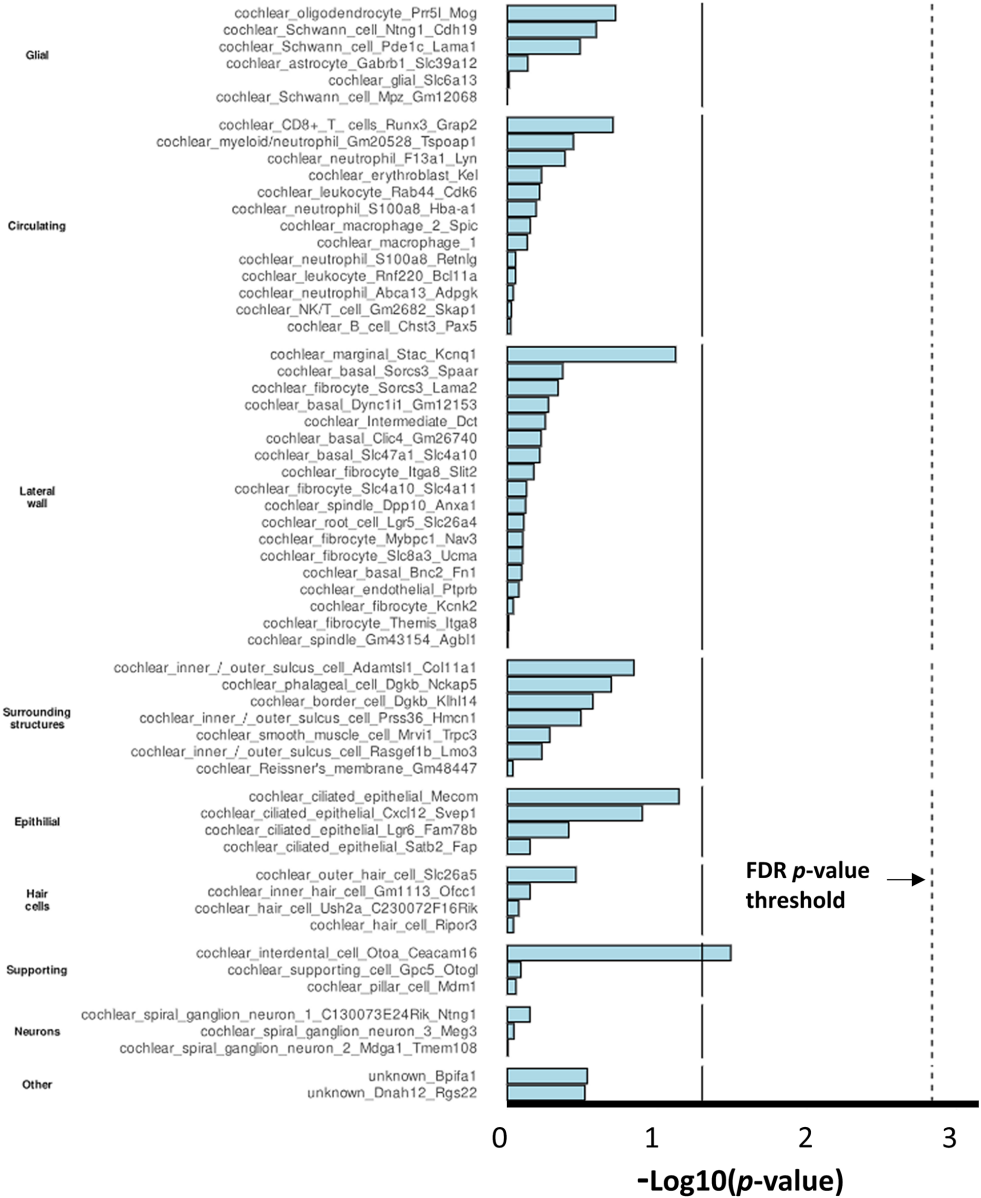
Cochlear cell line enrichment analysis based on single-cell transcriptomic data from mice^35^. The bar charts present *p*-values (in − log10) for the regression coefficients. The regression analysis was conducted with MAGMA.

The integrated analysis of GWAS findings and single-cell transcriptomic data could be a powerful tool to identify putative cell types involved in pathogenesis. Cochlear cell lines are likely involved in SIN deficits but are not included in FUMA. MAGMA-derived gene-specific effect size estimates obtained for SIN deficits were predicted from single-cell transcriptomic data to identify cochlear cell lines involved in SIN deficits. Cochlear interdental cells revealed marginally significant enrichment at *p* < 0.05. No cochlear cell types remained significant after applying the FDR correction (Fig. 5). These results suggest that cochlear cell lines are not likely involved in the pathogenesis of SIN deficits in individuals with self-reported normal hearing.

### Genetic comorbidities associated with SIN deficits

Genomic correlations between SIN deficits and health traits across the phenome were evaluated (e.g.,^46^). Sixty-five traits revealed a significant association with SIN deficits (adjusted *p* = 0.05/1458) (Supplementary file S2). Neuropsychiatric traits, such as worrying too long after embarrassment, guilt feelings, neuroticism score, miserableness, depression, schizophrenia, bipolar disorder, loneliness, and maternal history of severe depression, were significantly associated with SIN deficits. Sensory traits, such as hearing difficulty, tinnitus, and eye problems, revealed significant association with SIN deficits. Environmental factors, such as smoking and bread intake, were significantly correlated with SIN deficits.

### Results of the enrichment analysis after removing the HLA region

The HLA region is highly pleiotropic and associated with numerous health traits^47^, which might influence the enrichment analysis. The functional enrichment analysis was revised after removing the HLA region (Chr:6, Position: 25–34 GB) from the GWAS summary statistics (Supplementary file S3). The LDSC regression revealed no evidence of residual population stratification after removing the HLA region (h2(SE) = 0.0425 (0.0026), Lambda GC = 1.20, intercept(SE) = 1.0029 (0.0077), mean Chi2: 1.229). GWAS-based gene set enrichment analysis identified 60 health traits, including neuropsychiatric, metabolic, cardiovascular, inflammatory, sensory, and lifestyle traits. MAGMA analysis revealed that the GWAS signals were enriched in the brain cortex and anterior cingulate cortex. Partitioned heritability-based enrichment results remained significantly associated with SIN deficits even after removing the HLA region. No cochlear cell lines revealed significant enrichment with SIN deficits. The genomic correlation analysis showed 60 complex traits associated with SIN deficits. Several Reactome and GO biological processes previously associated showed no significant enrichment after removing the HLA region, suggesting those enrichment results were driven by the HLA region. Gene sets related to pyruvate metabolism and G alpha (i and q) signaling events remained associated with SIN deficits after removing the HLA region. Overall, the major results of genomic correlation analysis, tissue, cochlear cell type, and GWAS gene-set enrichment analysis remained significant even after excluding the HLA region from the enrichment analysis.

### Results of the replication analysis

**Figure 6:**
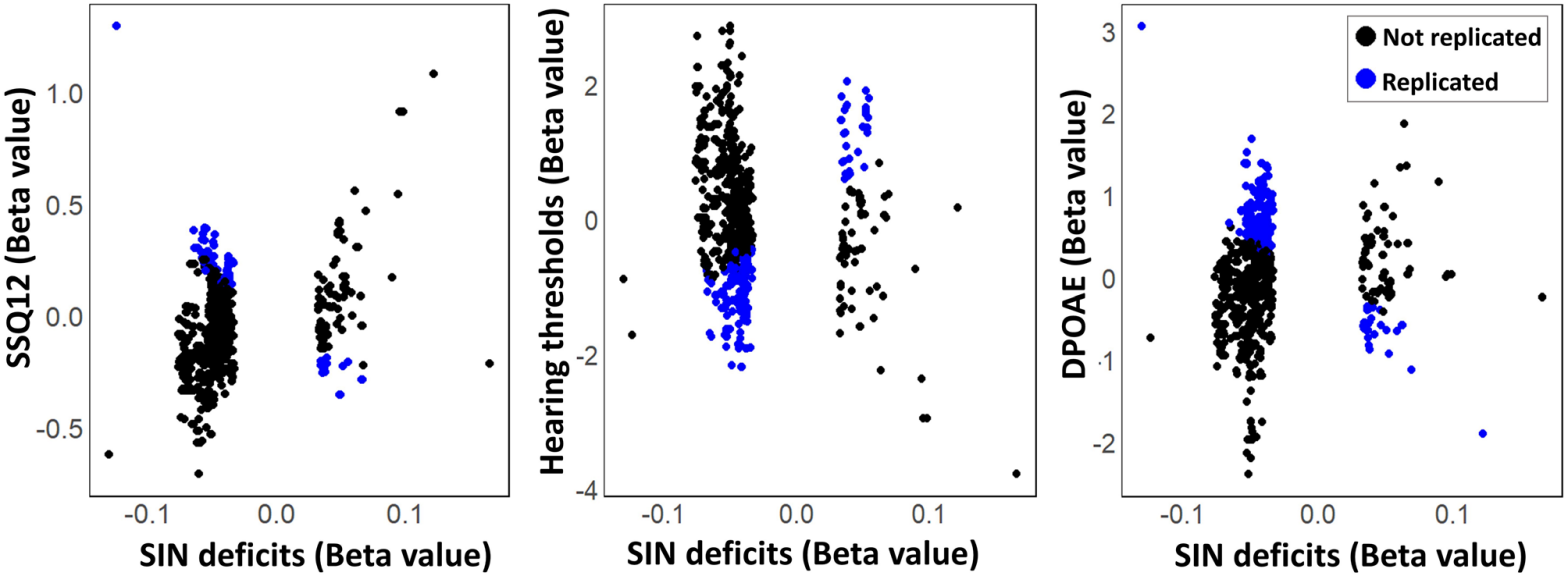
Scatter plots between the beta values of the SIN deficits GWAS versus HTs, DPOAEs, and SSQ12. SNPs achieving *p* < 0.05 and the beta values showing a consistent direction of association with GWAS were included. SIN deficits were coded as a binary phenotype for GWAS (0-control, 1-case). Low values of SSQ12 and DPOAEs and high values of HTs indicate poor physiological. Hence, to be replicated, SNPs achieving positive beta values for SIN deficits GWAS should exhibit negative beta values for SSQ12 and DPOAEs, and positive beta values for HTs, and vice versa. SNP-specific beta, *p*-values, and adjusted *p*-values for the audiological measures are presented in Supplementary file S5.

The replication analysis was conducted on 1549 SNPs (out of 1716 achieving *p* < 10^−6^ in GWAS) from 25 genomic loci associated with SIN deficits in GWAS. 167 SNPs with missing genotypes in the replication sample were excluded. We calculated the eigenvalues of the correlation matrix for 1549 SNPs to calculate the number of effective tests^44^. The *p*-value threshold was adjusted accordingly (adjusted *p*-value threshold = 0.05/182 = to 0.0002748; 182 is the number of effective tests after accounting for LD). 73 SNPs were replicated for SSQ12, with adjusted *p*-value < 0.05 and the direction of the beta consistent with the GWAS (Supplementary File S5). Among 73 SNPs replicating with SSQ12, 20 were near (or within) the loci involving *RNU6-471P* and *HLA-DRB1*, 48 were near *GRM3*, 4 were near *MAPT*, and one was in *BRINP3*. Among 119 SNPs replicating with HTs, 108 were near the loci involving *RNU6-471P* and *HLA-DRB1*, 4 were near *MAPT*, 3 were near *CDH12*, 2 were near *GRM3*, and 2 were near *NUF2*. Among 187 SNPs replicated with DPOAEs, 180 were within the loci involving *RNU6-471P* and *HLA-DRB1*, 4 were near *MAPT*, 2 were near *GRM3*, and one was near *NUF2*. 12 SNPs were replicated in all analyses, 66 in at least two analyses, and 211 in at least one analysis. Table 4 presents 13 SNPs replicated in all analyses. Top SNPs mapped to *MAPT*, *GRM3*, *HLA-DQA1*, *BRRINP3*, *U3*, and *RNA5SP63* revealed significant associations with SSQ12, HTs, and DPOAEs. For the locus near *GRM7* (Chromosome 3), 10 out of 16 SNPs revealed a promising pattern of association with SSQ12 (*p* < 0.05, adjusted *p* > 0.05). However, several SNPs revealed the reverse direction of association (adjusted* p* < 0.05) with DPOAEs and HTs, suggesting that SNPs associated with SIN deficits in GWAS revealed significantly better DPOAEs and HTs (Supplementary file S3). Figure 6 presents the scatter plots between beta values of GWAS and audiometric measures, allowing the visualization of the results for the replication analysis.

**Table 4:** SNPs associated with SSQ12, HTs, and DPOAEs in the replication sample.

SNP	Mapped genes	CHR	REF	ALT	ALT Freq	SIN deficits (GWAS)		SSQ12		HTs		DPOAEs	
						Beta	− Log10P	Beta	*p*-value	Beta	*p*-value	Beta	*p*-value
rs534654325	*HLA-DQA1*	6	AGGT	A	0.41	− 0.04	8.00*	0.21	3.1E−06	− 0.77	1.7E−07	0.36	0.0001
rs9271339	*HLA-DQA1*	6	T	C	0.46	− 0.04	7.96*	0.21	7.4E−06	− 0.76	3.4E−07	0.36	0.0001
rs9271336	*HLA-DQA1*	6	G	A	0.46	− 0.04	7.95*	0.21	7.4E−06	− 0.76	3.4E−07	0.36	0.0001
rs9271337	*HLA-DQA1*	6	C	T	0.46	− 0.04	7.95*	0.21	7.4E−06	− 0.76	3.4E−07	0.36	0.0001
rs9271338	*HLA-DQA1*	6	T	G	0.46	− 0.04	7.95*	0.21	7.4E−06	− 0.76	3.4E−07	0.36	0.0001
rs713522	*MAPT*	17	T	C	0.42	0.04	7.64*	− 0.27	5.4E−08	1.18	5.2E−14	− 0.93	7.0E−17
rs2471737	*MAPT*	17	A	G	0.42	0.04	7.51*	− 0.25	7.0E−07	1.02	1.4E−10	− 0.84	7.0E−17
rs2435200	*MAPT*	17	G	A	0.41	0.04	7.25	− 0.23	5.4E−06	0.96	4.1E−09	− 0.74	5.3E−13
rs6917212	*HLA-DQA1*	6	C	G	0.43	− 0.04	7.16	0.27	1.2E−08	− 0.79	1.1E−07	0.35	0.0002
rs2435201	*MAPT*	17	T	A	0.58	− 0.03	6.56	0.26	1.3E−08	− 0.87	3.6E−08	0.73	3.8E−13
rs10252502	*GRM3*	7	G	C	0.34	0.04	6.35	− 0.27	2.9E−06	0.75	0.00004	− 0.53	5.0E−06
rs6960188	*GRM3*	7	T	A	0.34	0.03	6.04	− 0.24	3.2E−05	0.70	0.0001	− 0.48	3.0E−05

*SIN deficits GWAS *p* < 5E−08.

## Discussion

The present GWAS was conducted on 279,911 participants from the UK Biobank database, with 58,847 cases reporting SIN deficits despite self-reported normal hearing in quiet and 221,067 controls without SIN deficits reporting self-reported normal hearing in quiet. The replication analysis was conducted on 242 healthy young adults with self-reported normal hearing. The major findings of the study were as follows: (1) The study identified 996 SNPs across four genomic loci. Two independent loci involving *MAPT* and *GRM3* were associated with SIN deficits. Twenty-one loci, including 720 SNPs, showed genome-wide suggestive association with SIN deficits (*p* < 10^−6^). (2) The replication analysis was performed using SSQ12, HTs, and DPOAEs. 73 SNPs were replicated for SSQ12, with adjusted *p*-value < 0.05, and the direction of the beta values consistent with GWAS. 13 SNPs were replicated for SSQ12, HTs, and DPOAEs. 66 were replicated for at least two measures and 211 (27.3%) in at least one analysis. (3) SNPs in *MAPT* (rs713522, rs2471737, rs2435200, rs2435201) and *GRM3* (rs10252502, rs6960188), were replicated for SSQ12, HTs, and DPOAEs. (4) GWAS signals were collectively enriched in brain tissues, including the entorhinal cortex, frontal cortex, hippocampus, and anterior cingulate cortex. Cell lines from the inferior temporal lobe, dorsolateral prefrontal cortex, anterior caudate, and angular gyrus were significantly enriched for SIN deficits. Cochlear cell lines revealed no significant association with SIN deficits. (5) GWAS signals were collectively enriched in biological processes, molecular functions, and pathways implemented in gene regulation, oxidative stress-induced senescence, genotoxicity, synaptic signal transduction, and metabolism. Gene sets related to pyruvate metabolism and G alpha (i and q) signaling events remained associated with SIN deficits after removing the HLA region. (6) GWAS gene set enrichment analysis identified neuropsychiatric, sensory, cognitive, metabolic, musculoskeletal, cardiovascular, inflammatory, neoplasm, and neuropathic traits sharing associated with SIN deficits. (7) Genetic comorbidities associated with SIN deficits were identified using genomic correlation. Several neuropsychiatric traits revealed significant association with SIN deficits.

### Association between SIN and a locus involving MAPT

The present study obtained a significant association between genetic variants in *MAPT* (Table 4). *MAPT* gene codes for microtubule-related protein tau, primarily expressed in neurons^48^. Accumulated and hyperphosphorylated tau forming neurofibrillary tangles (NFTs) is a critical mechanism associated with neurodegeneration and cognitive decline in AD^49^. Tau spreads to neural networks through synapses, causing synaptic and neuronal dysfunctions under pathological conditions^50,51^. Tau could modulate cellular response to oxidative stress^52^. Impulse noise exposure, a known risk factor of SIN deficits (e.g.,^53^), could trigger the accumulation of pathologic tau oligomers and neurofilaments in the auditory neurons^54^. Our results suggest that the *MAPT* locus lies at the crossroads between SIN deficits and AD-related dementia.

In addition to tauopathy, cognitive decline in AD-related dementia is associated with neuritic β-amyloid (Aβ) plaques resulting from the interplay between *APP* and *APOE*^55^. Aβ plaques are found in cortical and subcortical structures of AD patients^56^. NFTs and Aβ plaques, two interconnected pathological mechanisms associated with AD-related dementia and cognitive decline, could theoretically contribute to the association between SIN deficits and AD-related dementia observed in past epidemiological studies^57^. However, the results of the present GWAS showed no association between SIN deficits and genetic variants in *APOE* (e.g., rs429358 and rs7412, *p* < 0.05) and *APP*, indicating that the relationship between SIN deficits and AD-related dementia is likely driven by the *MAPT* locus.

**Figure 7:**
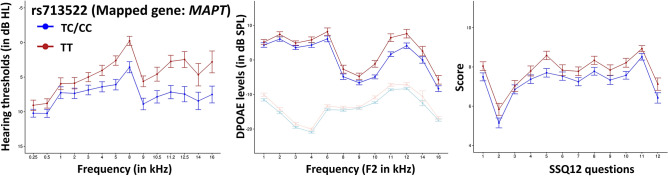
Line charts showing audiogram (left), DPgram (middle), and SSQ12 (right) results between individuals with rs713522 TT and TC/CC genotype. The error bar indicates a ± standard error. The light color lines in DPgrams indicate noise floor.

The present study observed associations between *MAPT* variants with SIN deficits, elevation in HTs, and reduction in DPOAEs. Suprathreshold auditory processing deficits are associated with AD-related dementia and cognitive impairment^58–61^. NFTs involved in AD-related cognitive declines are found in the central auditory nuclei and language areas responsible for SIN processing, and their concentration is correlated with aging in AD patients^62,63^. Phosphorylated tau protein levels in cochlear tissues gradually increase with aging in transgenic AD mice. The mice exhibit elevated thresholds of the auditory brainstem responses without a noticeable reduction in DPOAEs, indicating progressive spiral ganglion loss independent of cochlear hair cell functioning^64^. The present study observed a significantly lower SSQ12 in young adults carrying at least one risk allele for rs713522, consistent with the past animal studies associating the neurodegeneration of spiral ganglion neurons with AD-related dementia. Contrary to past animal studies, a significant reduction in DPOAEs and elevation in HTs were observed in young adults with at least one risk allele for rs713522 (Fig. 7). These results indicate that genetic variants in the *MAPT* locus could influence cochlear functioning in humans, potentially through non-autonomous mechanisms. Future research is necessary to investigate the pleotropic effects of the *MAPT* locus on auditory physiology.

### Evidence of glutamatergic mechanisms involved in SIN deficits

**Figure 8:**
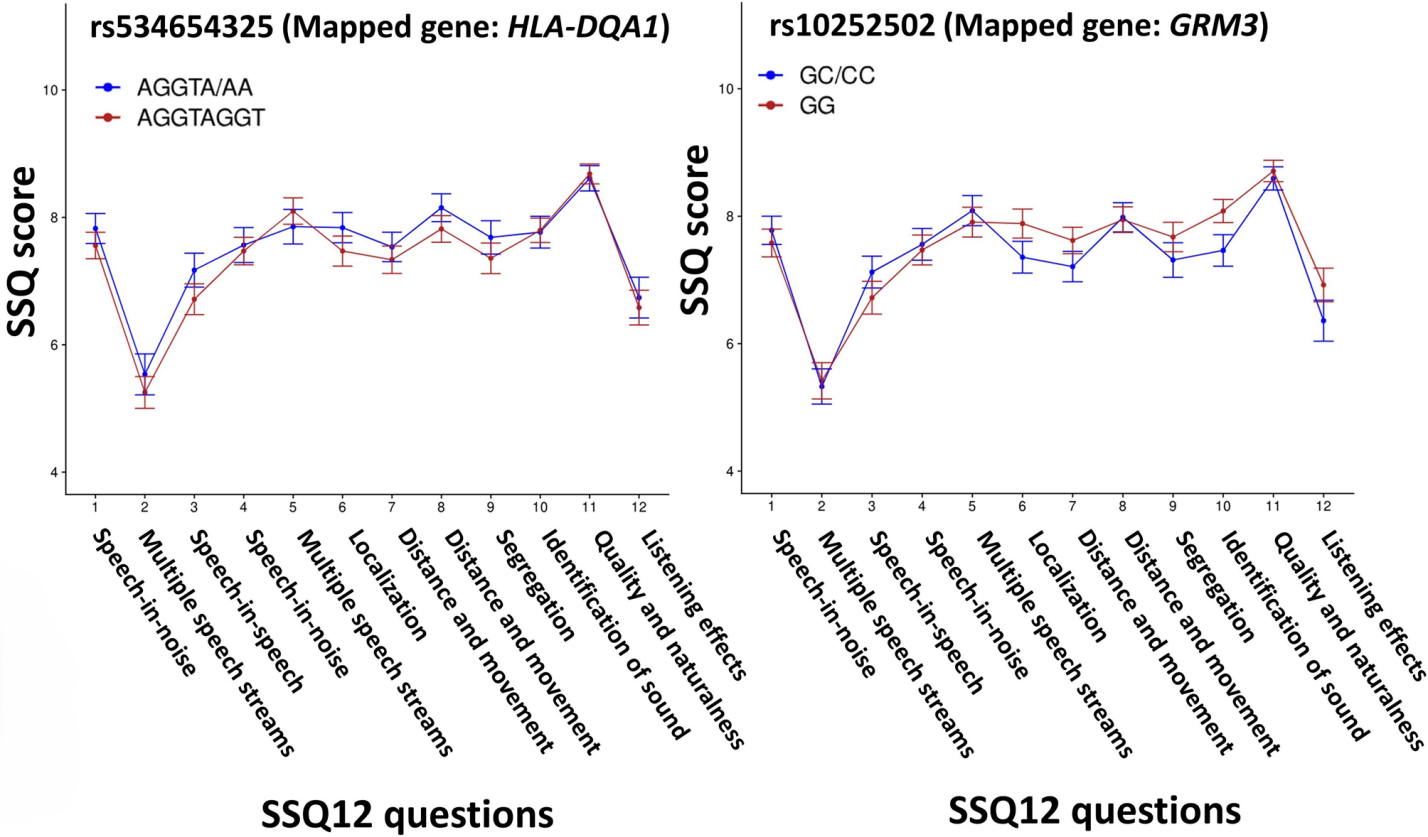
Line charts showing SSQ12 results for individuals carrying major alleles and at least minor alleles for SNPs achieving the highest replication scores close to *HLA-DQA1* and *GRM3*. These SNPs were significantly associated with SIN deficits in GWAS and were replicated with SSQ12, HTs, and DPOAEs. The error bar indicates a ± standard error.

SNPs in the locus near *GRM3* showed significant associations with SIN deficits (Fig. 8). 48 SNPs within the *GRM3* locus achieving GWAS suggestive significance at *p* < 10^−6^ were replicated with SSQ12, while two SNPs, rs10252502 and rs6960188, were replicated with SSQ12, HTs, and DPOAEs. SNPs in the *GRM3* locus have been associated with cognitive and metabolic traits^65,66^ (Fig. 2). Glutamate is the excitatory neurotransmitter expressed throughout the peripheral and central nervous systems. Mounting evidence links metabotropic glutamate receptor type 3 (mGluR3, encoded by *GRM3* gene) with cognitive traits and neuropsychiatric conditions, such as schizophrenia^67–69^. mGluR3s could be stimulated by glutamate, which is often co-released with N-acetylaspartyglutamate^70,71^. Reduced mGluR3 levels in the dorsolateral prefrontal cortex, a cortical area linked with working memory, selective attention, and cognitive controls^72^, are associated with compromised cognitive functions^73^. Impaired cognitive functioning could substantially influence SIN processing^10,74^. Furthermore, the association between DPOAEs and HTs suggested impaired cochlear functioning in carriers of *GRM3* variants. The functioning of the *GRM3* in human cochlear tissues remains elusive^75^. Single-cell transcriptomic studies in animals located *GRM3* in supporting cells^76^. *GRM3* activity in cochlear cells is downregulated following noise exposure^77^, a known risk factor for SIN deficits^78^. Therefore, the results indicate that the *GRM3* variants could influence cochlear physiology and cognitive processes involved in SIN deficits.

The locus near *GRM7* achieved suggestive significance in GWAS, and 10 SNPs within the locus showed a promising pattern of association with SSQ12 (*p* < 0.05, adjusted *p* > 0.05). Several SNPs within the locus revealed significant associations (adjusted *p* < 0.05) with HTs and DPOAEs, but they showed the opposite directions of effect than GWAS. Carriers of the lead SNP (rs116208485) with TT genotype showed higher odds of SIN deficits, and yet they revealed significantly better DPOAEs and HTs in both ears than those with CC and CT genotypes (Supplement File S1). This phenotypic pattern is associated with auditory neuropathy spectrum disorder, characterized by SIN deficits with normal or elevated DPOAEs^79–81^. *GRM7*, which encodes mGluR7, is critical in signal transduction through synaptic junctions to auditory neurons^82^. mGluR7 is localized in the efferent terminals from the medial olivocochlear system connecting to OHCs^83^. *GRM7* is expressed in the spiral ganglion cells, OHCs, IHCs, and supporting cells^84^. *GRM7* variants are associated with poorer speech perception and hearing sensitivity measures in candidate gene studies, with varying statistical support for their associations (for review,^85^). These variants were not replicated in the present study and in the past GWAS of age-related hearing difficulty^20,21^. Recent studies associated *GRM7* with cognitive and neuropsychiatric traits^86,87^. Taken together, our results indicate that the *GRM7* locus was associated with SIN deficits, possibly due to impaired cognitive functions; however, the involvement of the peripheral mechanisms could not be ruled out without further research.

### Enrichment analysis: tissues and cell lines involved in SIN deficits

The enrichment analysis was conducted to identify cell lines and tissues in which the genes associated with SIN deficits are robustly expressed. Cochlear cell lines did not reveal a strong association with SIN deficits. The inclusion of mice with hearing loss might contribute to the null results. We revised the analysis with the transcriptomic data derived from mice with normal hearing (Supplementary file S2). No cochlear cell lines revealed significant association with SIN deficits. Synaptic loss between IHCs and auditory nerve fibers is a putative mechanism underlying SIN deficits^88^. The synaptic gene expression profiles might not be robustly reflected in the cochlea transcriptomic profiles. The functional enrichment analysis identified gene ontology terms related to synaptic signal transduction for SIN deficits, but it remained unclear if the synaptic communication between IHCs and auditory nerve fibers was involved. The enrichment analysis using single-synaptosome transcriptome (e.g.,^89^), obtained from brain and cochlear tissues, could help identify synaptopathy mechanisms involved in SIN deficits.

Figure 4 presents the results of the heritability-based enrichment analysis. The dorsolateral prefrontal cortex, associated with executive control, working memory, theory of mind, mood regulation, predictive top-down mechanisms during speech processing, speaker-listener interaction, memory integration, and advanced linguistic processing, was significantly enriched for SIN deficits (for review,^90^). FUMA-based gene set analysis identified GW23 GABAergic neurons in the human prefrontal cortex. The inferior temporal lobe, an area associated with word recognition, revealed a significant association with SIN deficits^91^. Angular gyrus, associated with degraded speech comprehension^92^, and cingulate gyrus, involved in mediating the influence of cognitive load while SIN processing^93^, were associated with SIN deficits. The hippocampus and entorhinal cortex involved in suprathreshold auditory processing were significantly enriched^94^. In conclusion, the enrichment analysis identified brain tissues and cell lines involved in SIN deficits, complementing the past observational studies.

### Ontology annotations and genetic comorbidities associated with SIN deficits

SIN deficits showed association with neuropsychiatric, cognitive, neural, metabolic, cardiovascular, and cancer-related traits (Fig. 3). Cognitive traits, such as general cognitive abilities, cognitive decline, reaction time, and intelligence, were associated with SIN deficits^7,95,96^. SIN deficits have been associated with schizophrenia, AD, and autism spectrum disorder^57,97^. Metabolic traits revealed significant associations with SIN deficits^98,99^. The enrichment analysis identified novel associations with SIN deficits, such as hand grip strength, ulna and radius bone mineral density, and lung cancer, which should be investigated in future mechanistic and observational studies.

Functional enrichment analysis identified pathways, biological processes, and molecular functions involved in SIN deficits. Gene ontology terms related to synaptic signal transduction revealed a significant association with SIN deficits. These results are consistent with a growing body of literature showing the influence of age-related and noise-induced synaptopathy on suprathreshold auditory coding deficits^88^. Epigenetic gene regulation terms, such as deacetylate histones, DNA and histone methylations, epigenetic regulation of rRNA, and transcriptional regulation by small RNAs, highlighted the influence of epigenetic machinery on SIN deficits. Aging, occupational noise, and recreational noise/music exposures, known risk factors for SIN deficits, can influence the epigenetic landscape^100^. Canonical Wnt and notch pathways implemented in cell proliferation, differentiation, and cell death signaling during embryological development, maturation, and aging were significantly associated with SIN deficits^101^. DNA damage and repair canonical pathways, such as DNA repair, DNA double-strand break response and repair, and DNA damage telomere stress-induced senescence, implemented in acquired hearing loss, showed significant association with SIN deficits^102^. Notably, gene sets related to pyruvate metabolism and G alpha (i and q) signaling events remained associated with SIN deficits after removing the HLA region (Supplementary file S3), suggesting genes within the HLA region were driving several enrichment results. The HLA region is highly pleiotropic and imposes numerous methodological challenges for accurate genotyping^103^. Future mechanistic studies are required to elucidate their association with SIN deficits.

### Conclusions

We conducted a GWAS on 279,911 participants reporting normal hearing, 58,847 cases with deficits, and 221,067 controls without SIN deficits. We identified 996 SNPs across four genomic loci showing significant association with SIN deficits in participants with self-reported normal hearing. 720 SNPs across 21 loci achieved suggestive significance. The replication analysis on 242 healthy young adults was conducted, and 73 SNPs were replicated using a self-reported SIN perception measure of SSQ12. 12 SNPs close to *MAPT*, *GRM3*, and HLA-class genes in four loci associated with SIN deficits were replicated for SSQ12, HTs, and DPOAEs. SNPs near *GRM7* showed suggestive association with SIN deficits. GWAS signals were enriched in brain tissues, such as the dorsolateral prefrontal cortex, entorhinal cortex, frontal cortex, hippocampus, and inferior temporal cortex. Cochlear cell types revealed no significant enrichment with SIN deficits. GWAS identified shared gene sets between SIN deficits and neuropsychiatric, sensory, cognitive, metabolic, cardiovascular, inflammatory, and neoplasm traits. In summary, the present GWAS highlighted a polygenic architecture underlying SIN deficits in individuals with self-reported normal hearing.

### Supplementary Information

Supplementary Information 1.

Supplementary Information 2.

Supplementary Information 3.

Supplementary Information 4.

Supplementary Information 5.

Supplementary Information 6.

## Data Availability

The study used the UK Biobank database. The database is publicly available through the UK Biobank website: https://www.ukbiobank.ac.uk/. The data used for the replication analysis will be shared on dbGaP after the completion of the project: R21DC016704-01A1. The data used for cochlear cell type enrichment analysis were obtained from Boussaty et al. (2023) from the following: https://umgear.org//index.html?share_id=f526abfe&gene_symbol_exact_match=1. GWAS summary statistics can be found at: https://my.locuszoom.org/gwas/752608/?token=3b4cea1d3178406eaaf2ea2e92429332.

## Abbreviations

GWAS: Genome-wide association study
SNP: Single nucleotide polymorphism

## References

[1] Dubno JR, Dirks DD, Morgan DE (1984). Effects of age and mild hearing loss on speech recognition in noise. J. Acoust. Soc. Am..

[2] Spankovich C, Gonzalez VB, Su D, Bishop CE (2018). Self reported hearing difficulty, tinnitus, and normal audiometric thresholds, the national health and nutrition examination survey 1999–2002. Hear. Res..

[3] Tremblay KL, Pinto A, Fischer ME, Klein BE, Klein R, Levy S (2015). Self-reported hearing difficulties among adults with normal audiograms: The Beaver Dam offspring study. Ear Hear..

[4] Parthasarathy A, Hancock KE, Bennett K, DeGruttola V, Polley DB (2020). Bottom-up and top-down neural signatures of disordered multi-talker speech perception in adults with normal hearing. Elife.

[5] Pryce H, Wainwright D (2008). Help-seeking for medically unexplained hearing difficulties: A qualitative study. Int. J. Ther. Rehabil..

[6] Cooper JC, Gates GA (1991). Hearing in the elderly—The Framingham cohort, 1983–1985: Part II. Prevalence of central auditory processing disorders. Ear Hear..

[7] Jiang K, Armstrong NM, Agrawal Y, Gross AL, Schrack JA, Lin FR (2022). Associations of audiometric hearing and speech-in-noise performance with cognitive decline among older adults: The Baltimore longitudinal study of aging (BLSA). Front. Neurol..

[8] Lee SJ, Park KW, Kim LS, Kim H (2018). Association between frontal-executive dysfunction and speech-in-noise perception deficits in mild cognitive impairment. J. Clin. Neurol..

[9] Watson CJ, Waters S, Jacobs BM, Foote IF, Dey S, Noyce AJ (2022). Speech-in-noise perception is a marker of preclinical Alzheimer’s disease. J. Neurol. Neurosurg. Psychiatry.

[10] Anderson S, White-Schwoch T, Parbery-Clark A, Kraus N (2013). A dynamic auditory-cognitive system supports speech-in-noise perception in older adults. Hear. Res..

[11] Gervain J, Geffen MN (2019). Efficient neural coding in auditory and speech perception. Trends Neurosci..

[12] Holmes E, Griffiths TD (2019). 'Normal'hearing thresholds and fundamental auditory grouping processes predict difficulties with speech-in-noise perception. Sci. Rep..

[13] Darwin CJ (1997). Auditory grouping. Trends Cogn. Sci..

[14] Wang X, Xu L (2021). Speech perception in noise: Masking and unmasking. J. Otol..

[15] Yeend I, Beach EF, Sharma M (2019). Working memory and extended high-frequency hearing in adults: Diagnostic predictors of speech-in-noise perception. Ear Hear..

[16] Song, J., Martin, L. & Iverson, P. Native and non-native speech recognition in noise: Neural measures of auditory and lexical processing. In *International Congress of Phonetic Sciences* 5–9 (2019).

[17] Torkildsen JVK, Hitchins A, Myhrum M, Wie OB (2019). Speech-in-noise perception in children with cochlear implants, hearing aids, developmental language disorder and typical development: The effects of linguistic and cognitive abilities. Front. Psychol..

[18] Vermiglio AJ, Soli SD, Freed DJ, Fisher LM (2012). The relationship between high-frequency pure-tone hearing loss, hearing in noise test (HINT) thresholds, and the articulation index. J. Am. Acad. Audiol..

[19] Brewer CC, Zalewski CK, King KA, Zobay O, Riley A, Ferguson MA (2016). Heritability of non-speech auditory processing skills. Eur. J. Hum. Genet..

[20] Wells HR, Freidin MB, Abidin FNZ, Payton A, Dawes P, Munro KJ (2019). GWAS identifies 44 independent associated genomic loci for self-reported adult hearing difficulty in UK Biobank. Am. J. Hum. Genet..

[21] Trpchevska N, Freidin MB, Broer L, Oosterloo BC, Yao S, Zhou Y (2022). Genome-wide association meta-analysis identifies 48 risk variants and highlights the role of the stria vascularis in hearing loss. Am. J. Hum. Genet..

[22] Ivarsdottir EV, Holm H, Benonisdottir S, Olafsdottir T, Sveinbjornsson G, Thorleifsson G (2021). The genetic architecture of age-related hearing impairment revealed by genome-wide association analysis. Commun. Biol..

[23] Kalra G, Milon B, Casella AM, Herb BR, Humphries E, Song Y (2020). Biological insights from multi-omic analysis of 31 genomic risk loci for adult hearing difficulty. PLoS Genet..

[24] Croston R, Branch CL, Kozlovsky DY, Dukas R, Pravosudov VV (2015). Heritability and the evolution of cognitive traits. Behav. Ecol..

[25] Sindhusake D, Mitchell P, Smith W, Golding M, Newall P, Hartley D, Rubin G (2001). Validation of self-reported hearing loss. The Blue Mountains hearing study. Int. J. Epidemiol..

[26] Noble W, Jensen NS, Naylor G, Bhullar N, Akeroyd MA (2013). A short form of the speech, spatial and qualities of hearing scale suitable for clinical use: The SSQ12. Int. J. Audiol..

[27] Mishra SK, Saxena U, Rodrigo H (2022). Extended high-frequency hearing impairment despite a normal audiogram: Relation to early aging, speech-in-noise perception, cochlear function, and routine earphone use. Ear Hear..

[28] Welsh S, Peakman T, Sheard S, Almond R (2017). Comparison of DNA quantification methodology used in the DNA extraction protocol for the UK Biobank cohort. BMC Genom..

[29] Bycroft, C. *et al.* The UK Biobank resource with deep phenotyping and genomic data. *Nature* **562**(7726), 203–209 (2018). 10.1038/s41586-018-0579-z PMC6786975 30305743

[30] Mbatchou J, Barnard L, Backman J, Marcketta A, Kosmicki JA, Ziyatdinov A (2021). Computationally efficient whole-genome regression for quantitative and binary traits. Nat. Genet..

[31] Watanabe K, Taskesen E, Van Bochoven A, Posthuma D (2017). Functional mapping and annotation of genetic associations with FUMA. Nat. Commun..

[32] Bulik-Sullivan BK, Loh PR, Finucane HK, Ripke S, Yang J, Schizophrenia Working Group of the Psychiatric Genomics Consortium (2015). LD score regression distinguishes confounding from polygenicity in genome-wide association studies. Nat. Genet..

[33] Finucane HK, Bulik-Sullivan B, Gusev A, Trynka G, Reshef Y, Loh PR (2015). Partitioning heritability by functional annotation using genome-wide association summary statistics. Nat. Genet..

[34] Cuellar-Partida, G., Lundberg, M., Fang Kho, P., D’Urso, S., Gutiérrez-Mondragón, L. F., Thanh Ngo, T. & Hwang, L. D. Complex-traits genetics virtual lab: A community-driven web platform for post-GWAS analyses. *BioRxiv* 518027 (2019).

[35] Boussaty EC, Tedeschi N, Novotny M, Ninoyu Y, Du E, Draf C (2023). Cochlear transcriptome analysis of an outbred mouse population (CFW). Front. Cell. Neurosci..

[36] Clifford RE, Maihofer AX, Chatzinakos C, Coleman JR, Daskalakis NP, Gasperi M (2024). Genetic architecture distinguishes tinnitus from hearing loss. Nat. Commun..

[37] Orvis J, Gottfried B, Kancherla J, Adkins RS, Song Y, Dror AA (2021). gEAR: Gene expression analysis resource portal for community-driven, multi-omic data exploration. Nat. Methods.

[38] Satija R, Farrell JA, Gennert D, Schier AF, Regev A (2015). Spatial reconstruction of single-cell gene expression data. Nat. Biotechnol..

[39] Blake JA, Baldarelli R, Kadin JA, Richardson JE, Smith CL, Bult CJ (2021). Mouse genome database (MGD): Knowledgebase for mouse–human comparative biology. Nucleic Acids Res..

[40] de Leeuw CA, Mooij JM, Heskes T, Posthuma D (2015). MAGMA: Generalized gene-set analysis of GWAS data. PLoS Comput. Biol..

[41] Bhatt IS, Lichtenhan J, Tyler R, Goodman S (2023). Influence of tinnitus, lifetime noise exposure, and firearm use on hearing thresholds, distortion product otoacoustic emissions, and their relative metric. J. Acoust. Soc. Am..

[42] Rubinacci S, Ribeiro DM, Hofmeister RJ, Delaneau O (2021). Efficient phasing and imputation of low-coverage sequencing data using large reference panels. Nat. Genet..

[43] Bates, D., Mächler, M., Bolker, B. & Walker, S. *Fitting Linear Mixed-Effects Models Using lme4*. arXiv preprint arXiv:1406.5823 (2014).

[44] Li J, Ji L (2005). Adjusting multiple testing in multilocus analyses using the eigenvalues of a correlation matrix. Heredity.

[45] Valderrama JT, De la Torre A, McAlpine D (2022). The hunt for hidden hearing loss in humans: From preclinical studies to effective interventions. Front. Neurosci..

[46] Bulik-Sullivan B, Finucane HK, Anttila V, Gusev A, Day FR, Loh PR (2015). An atlas of genetic correlations across human diseases and traits. Nat. Genet..

[47] Dendrou CA, Petersen J, Rossjohn J, Fugger L (2018). HLA variation and disease. Nat. Rev. Immunol..

[48] Grundke-Iqbal I, Iqbal K, Quinlan M, Tung YC, Zaidi MS, Wisniewski HM (1986). Microtubule-associated protein tau. A component of Alzheimer paired helical filaments. J. Biol. Chem..

[49] Naseri NN, Wang H, Guo J, Sharma M, Luo W (2019). The complexity of tau in Alzheimer’s disease. Neurosci. Lett..

[50] DeVos SL, Corjuc BT, Oakley DH, Nobuhara CK, Bannon RN, Chase A (2018). Synaptic tau seeding precedes tau pathology in human Alzheimer’s disease brain. Front. Neurosci..

[51] Calafate S, Buist A, Miskiewicz K, Vijayan V, Daneels G, De Strooper B (2015). Synaptic contacts enhance cell-to-cell tau pathology propagation. Cell Rep..

[52] Ittner LM, Ke YD, Delerue F, Bi M, Gladbach A, van Eersel J (2010). Dendritic function of tau mediates amyloid-β toxicity in Alzheimer’s disease mouse models. Cell.

[53] Grant KW, Phatak SA, Myers JR, Jenkins KA, Kubli LR, Brungart DS (2023). Functional hearing difficulties in blast-exposed service members with normal to near-normal hearing thresholds. Ear Hear..

[54] Du X, West MB, Cai Q, Cheng W, Ewert DL, Li W (2017). Antioxidants reduce neurodegeneration and accumulation of pathologic Tau proteins in the auditory system after blast exposure. Free Radic. Biol. Med..

[55] Hoe HS, William Rebeck G (2008). Functional interactions of APP with the apoE receptor family. J. Neurochem..

[56] DeTure MA, Dickson DW (2019). The neuropathological diagnosis of Alzheimer’s disease. Mol. Neurodegener..

[57] Stevenson JS, Clifton L, Kuźma E, Littlejohns TJ (2022). Speech-in-noise hearing impairment is associated with an increased risk of incident dementia in 82,039 UK Biobank participants. Alzheimer’s Dement..

[58] Edwards JD, Xu H, Clark DO, Guey LT, Ross LA, Unverzagt FW (2017). Speed of processing training results in lower risk of dementia. Alzheimer’s Dement. Transl. Res. Clin. Interv..

[59] Idrizbegovic E, Hederstierna C, Dahlquist M, Kämpfe Nordström C, Jelic V, Rosenhall U (2011). Central auditory function in early Alzheimer’s disease and in mild cognitive impairment. Age Ageing.

[60] Gates GA, Anderson ML, Feeney MP, McCurry SM, Larson EB (2008). Central auditory dysfunction in older persons with memory impairment or Alzheimer dementia. Arch. Otolaryngol. Head Neck Surg..

[61] Gates GA, Beiser A, Rees TS, D'Agostino RB, Wolf PA (2002). Central auditory dysfunction may precede the onset of clinical dementia in people with probable Alzheimer’s disease. J. Am. Geriatr. Soc..

[62] Arnold SE, Hyman BT, Flory J, Damasio AR, Van Hoesen GW (1991). The topographical and neuroanatomical distribution of neurofibrillary tangles and neuritic plaques in the cerebral cortex of patients with Alzheimer’s disease. Cereb. Cortex.

[63] Esiri MM, Pearson RCA, Powell TPS (1986). The cortex of the primary auditory area in Alzheimer’s disease. Brain Res..

[64] Wang SE, Wu CH (2021). Tau phosphorylation and cochlear apoptosis cause hearing loss in 3× Tg-AD Mouse model of Alzheimer’s disease. Chin. J. Physiol..

[65] Kichaev G, Bhatia G, Loh PR, Gazal S, Burch K, Freund MK (2019). Leveraging polygenic functional enrichment to improve GWAS power. Am. J. Hum. Genet..

[66] Lee JJ, Wedow R, Okbay A, Kong E, Maghzian O, Zacher M (2018). Gene discovery and polygenic prediction from a 1.1-million-person GWAS of educational attainment. Nat. Genet..

[67] Pantelis C, Papadimitriou GN, Papiol S, Parkhomenko E, Pato MT, Paunio T (2014). Biological insights from 108 schizophrenia-associated genetic loci. Nature.

[68] Neale JH, Olszewski R (2019). A role for N-acetylaspartylglutamate (NAAG) and mGluR3 in cognition. Neurobiol. Learn. Mem..

[69] Egan MF, Straub RE, Goldberg TE, Yakub I, Callicott JH, Hariri AR (2004). Variation in GRM3 affects cognition, prefrontal glutamate, and risk for schizophrenia. Proc. Natl. Acad. Sci..

[70] Forloni G, Grzanna R, Blakely RD, Coyle JT (1987). Co-localization of N-acetyl-aspartyl-glutamate in central cholinergic, noradrenergic, and serotonergic neurons. Synapse.

[71] Tsai G, Stauch BL, Vornov JJ, Deshpande JK, Coyle JT (1990). Selective release ofN-acetylaspartylglutamate from rat optic nerve terminals in vivo. Brain Res..

[72] Szczepanski SM, Knight RT (2014). Insights into human behavior from lesions to the prefrontal cortex. Neuron.

[73] Ghose S, Gleason KA, Potts BW, Lewis-Amezcua K, Tamminga CA (2009). Differential expression of metabotropic glutamate receptors 2 and 3 in schizophrenia: A mechanism for antipsychotic drug action?. Am. J. Psychiatry.

[74] Senkowski D, Moran JK (2022). Early evoked brain activity underlies auditory and audiovisual speech recognition deficits in schizophrenia. NeuroImage Clin..

[75] Lu Y (2014). Metabotropic glutamate receptors in auditory processing. Neuroscience.

[76] Tuset MP, Wiefels MD, McKenna K, Mittal J, Gowda C, Mittal R, Eshraghi AA (2023). Single-cell sequencing: A powerful technique to understand the pathophysiology of auditory disorders. Front. Audiol. Otol..

[77] Maeda Y, Kariya S, Uraguchi K, Takahara J, Fujimoto S, Sugaya A, Nishizaki K (2021). Immediate changes in transcription factors and synaptic transmission in the cochlea following acoustic trauma: A gene transcriptome study. Neurosci. Res..

[78] Bhatt IS, Washnik N, Torkamani A (2022). Suprathreshold auditory measures for detecting early-stage noise-induced hearing loss in young adults. J. Am. Acad. Audiol..

[79] Starr A, Sininger Y, Nguyen T, Michalewski HJ, Oba S, Abdala C (2001). Cochlear receptor (microphonic and summating potentials, otoacoustic emissions) and auditory pathway (auditory brain stem potentials) activity in auditory neuropathy. Ear Hear..

[80] Gabr T, Elakkad MA (2023). Auditory neuropathy spectrum disorder (ANSD): A distortion product otoacoustic emissions (DPOAEs) study. Egypt. J. Otolaryngol..

[81] Hood LJ, Berlin CI, Bordelon J, Rose K (2003). Patients with auditory neuropathy/dys-synchrony lack efferent suppression of transient evoked otoacoustic emissions. J. Am. Acad. Audiol..

[82] Klotz L, Enz R (2021). MGluR7 is a presynaptic metabotropic glutamate receptor at ribbon synapses of inner hair cells. FASEB J..

[83] Fujikawa T, Petralia RS, Fitzgerald TS, Wang YX, Millis B, Morgado-Díaz JA (2014). Localization of kainate receptors in inner and outer hair cell synapses. Hear. Res..

[84] Friedman RA, Van Laer L, Huentelman MJ, Sheth SS, Van Eyken E, Corneveaux JJ (2009). GRM7 variants confer susceptibility to age-related hearing impairment. Hum. Mol. Genet..

[85] Wells HRR, Newman TA, Williams FM (2020). Genetics of age-related hearing loss. J. Neurosci. Res..

[86] Nygaard M, Dowsett J, McGue M, Christensen K, Christiansen L, Tan Q, Mengel-From J (2021). Genome-wide association analysis of cognitive function in Danish long-lived individuals. Mech. Ageing Dev..

[87] Squillario M, Abate G, Tomasi F, Tozzo V, Barla A, Uberti D (2020). A telescope GWAS analysis strategy, based on SNPs-genes-pathways ensamble and on multivariate algorithms, to characterize late onset Alzheimer’s disease. Sci. Rep..

[88] Liberman MC, Kujawa SG (2017). Cochlear synaptopathy in acquired sensorineural hearing loss: Manifestations and mechanisms. Hear. Res..

[89] Niu, M. *et al.* Droplet-based transcriptome profiling of individual synapses. *Nat. Biotechnol.* **41**(9), 1332–1344 (2023). 10.1038/s41587-022-01635-1 36646931

[90] Hertrich I, Dietrich S, Blum C, Ackermann H (2021). The role of the dorsolateral prefrontal cortex for speech and language processing. Front. Hum. Neurosci..

[91] Nobre AC, Allison T, McCarthy G (1994). Word recognition in the human inferior temporal lobe. Nature.

[92] Hartwigsen G, Golombek T, Obleser J (2015). Repetitive transcranial magnetic stimulation over left angular gyrus modulates the predictability gain in degraded speech comprehension. Cortex.

[93] Gennari SP, Millman RE, Hymers M, Mattys SL (2018). Anterior paracingulate and cingulate cortex mediates the effects of cognitive load on speech sound discrimination. NeuroImage.

[94] Armstrong NM, Croll PH, Oosterloo BC, Lin FR, Ikram MA, Goedegebure A, Vernooij MW (2020). Association of speech recognition thresholds with brain volumes and white matter microstructure: The Rotterdam study. Otol. Neurotol..

[95] Price CN, Bidelman GM (2021). Attention reinforces human corticofugal system to aid speech perception in noise. Neuroimage.

[96] Heinrich A, Henshaw H, Ferguson MA (2015). The relationship of speech intelligibility with hearing sensitivity, cognition, and perceived hearing difficulties varies for different speech perception tests. Front. Psychol..

[97] Dunlop WA, Enticott PG, Rajan R (2016). Speech discrimination difficulties in high-functioning autism spectrum disorder are likely independent of auditory hypersensitivity. Front. Hum. Neurosci..

[98] Bhatt, I. S. *et al.* Polygenic risk score-based association analysis of speech-in-noise and hearing threshold measures in healthy young adults with self-reported normal hearing. *J. Assoc. Res. Otolaryngol.* **24**(5), 513–525 (2023). 10.1007/s10162-023-00911-4 PMC10695896 37783963

[99] Morales EEG, Ting J, Gross AL, Betz JF, Jiang K, Du S (2022). Association of cigarette smoking patterns over 30 years with audiometric hearing impairment and speech-in-noise perception: The atherosclerosis risk in communities study. JAMA Otolaryngol. Head Neck Surg..

[100] Eze IC, Jeong A, Schaffner E, Rezwan FI, Ghantous A, Foraster M (2020). Genome-wide DNA methylation in peripheral blood and long-term exposure to source-specific transportation noise and air pollution: the SAPALDIA study. Environ. Health Perspect..

[101] Samarajeewa A, Jacques BE, Dabdoub A (2019). Therapeutic potential of Wnt and Notch signaling and epigenetic regulation in mammalian sensory hair cell regeneration. Mol. Ther..

[102] Wu J, Ye J, Kong W, Zhang S, Zheng Y (2020). Programmed cell death pathways in hearing loss: A review of apoptosis, autophagy and programmed necrosis. Cell Prolif..

[103] Uffelmann E, Huang QQ, Munung NS, De Vries J, Okada Y, Martin AR (2021). Genome-wide association studies. Nat. Rev. Methods Prim..

