# Supplementary material for: A genome-wide association study reveals a polygenic architecture of speech-in-noise deficits in individuals with self-reported normal hearing
Source: Sci Rep. 2024 Jun 7;14:13089. doi: 10.1038/s41598-024-63972-2 (PMC11161523; doi:10.1038/s41598-024-63972-2)
Supplement: Supplementary file 1 — Supplementary Information 1. [file 41598_2024_63972_MOESM1_ESM.docx]

Supplement File S1: Results of the genome-wide association study (GWAS) for the speech-in-noise (SIN) phenotype.

- Table 1: Prevalence of hearing difficulty (data field: 2247) across the UK Biobank sample. The hearing difficulty measure was derived using the highest category from instances 0-3.
- Table 2: Prevalence of hearing difficulty in noise (data field: 2257) across the UK Biobank sample. The hearing difficulty in noise measure was derived using the highest category from instances 0-3.
- Figure 1: A schematic diagram showing the subject selection criteria and their influence on the sample size.
- Figure 2: LocusZoom results of the genomic loci showing significant association with the SIN phenotype.
- Figure 3: Manhattan plot: Gene-based test (FUMA) – SIN phenotype
- Figure 4: MAGMA Tissue expression analysis
- Figure 5: Quantile-Quantile (Q-Q) plots of expected and observed *p*-values (converted on a -log10 (*p*-value) scale) for the genome-wide association study of the SIN phenotype. The plot on the right-hand side shows Q-Q plots for minor allele frequency-based categories and genomic inflation measurement (GC lambda).
- Figure 6: Results of the enrichment analysis: SIN phenotype
- Figure 7: Results of the phenome-wide association analysis for the lead SNP rs539509361 derived from Zhou et al. (2018)
- Figure 8: Results of the replication analysis.
- Figure 9: Hearing thresholds and distortion-product otoacoustic emissions (DPOAEs) between individuals with rs116208485 TT and TC/CC variants.
- Figure 10: The results of the cochlear cell-line enrichment analysis using single-cell transcriptomic data from mice cochlear tissues showing normal hearing thresholds.

Supplement File S2: The results of the enrichment analysis for the SIN phenotype

- Supplement File S2 – GS: The table presents the gene sets associated with the SIN phenotype.
- Supplement File S2 – gtex_v8_ts_DEG: The table presents the results of the tissue enrichment analysis for the SIN phenotype.
- Supplement File S2 – IndSigSNPs: The table presents the significant independent SNPs associated with the SIN phenotype.
- Supplement File S2 – MAGMA celltype GSE104276 Human Prefrontal cortex all ages.
- Supplement File S2 – Genomic correlations: Results of the genomic correlation analysis.

Supplement File S3: Results of the enrichment analysis for SIN deficits after removing the MHC region (Chr:6, BP:25-34 GB) from the genome-wide association study (GWAS) summary statistics.

- **Figure 1:** The GWAS Manhattan plot after removing the HLA region (Chr:6, BP:25-34 GB).
- **Figure 2:** Results of the enrichment analysis: the SIN phenotype after removing the HLA region (Chr:6, BP:25-34 GB)
- **Figure 3:** Manhattan plot: Gene-based test (FUMA) using the GWAS summary statistics after removing the HLA region (Chr:6, BP:25-34 GB).
- **Figure 4:** MAGMA Tissue expression analysis using the GWAS summary statistics after removing the HLA region (Chr:6, BP:25-34 GB).
- **Figure. 5**: Cell- and tissue-specific partitioning heritability analysis of SIN deficits after removing the HLA region from the GWAS summary statistics.
- **Figure. 6**: The results of the cochlear cell-line enrichment analysis using single-cell transcriptomic data from mice cochlear tissues after removing the HLA region from the GWAS summary statistics.

Supplement File S4: The results of the enrichment analysis for the SIN phenotype

- Supplement File S2 – GS: The table presents the gene sets associated with the SIN phenotype after removing the HLA region (Chr:6, BP:25-34 GB).
- Supplement File S2 – gtex_v8_ts_DEG: The table presents the results of the tissue enrichment analysis for the SIN phenotype after removing the HLA region (Chr:6, BP:25-34 GB).
- Supplement File S2 – IndSigSNPs: The table presents the significant independent SNPs associated with the SIN phenotype after removing the HLA region (Chr:6, BP:25-34 GB).
- Supplement File S2 – Genomic correlations: Results of the LDSC regression-based cell-type enrichment analysis after removing the HLA region (Chr:6, BP:25-34 GB).

Supplement File S5: The results of the replication analysis for SNPs achieving *p*<10^-6^ in GWAS. The table presents GWAS summary statistics and the results of the replication analysis for SNPs achieving *p*<10-6 in SIN deficits GWAS. Color shades represent chromosomes. SSQ12 was the primary replication measure. The SIN deficits phenotype was coded binary (0-control, 1-case) for GWAS. Lower values of SSQ12 indicate poorer physiology; hence, SNPs achieving positive beta values for GWAS should reveal negative beta values for SSQ12 and vice versa. Similarly, HTs and DPOAEs were assigned replication scores (more details in the main manuscript). The column filters allow user-defined visualization of the results.
