# Supplementary material for: A genome-wide association study reveals a polygenic architecture of speech-in-noise deficits in individuals with self-reported normal hearing
Source: Sci Rep. 2024 Jun 7;14:13089. doi: 10.1038/s41598-024-63972-2 (PMC11161523; doi:10.1038/s41598-024-63972-2)

**Supplement File S1:** Results of the genome-wide association study (GWAS) for the speech-in-noise (SIN) phenotype.

**Table 1**: Prevalence of hearing difficulty (data field: 2247) across the UK Biobank sample. The hearing difficulty measure was derived using the highest category from instances 0-3.

|  | Instance 0 (2006-10) | Instance 1 (2012-13) | Instance 2  (2014+) | Instance 3  (2019+) | Derived hearing difficulty |
| --- | --- | --- | --- | --- | --- |
| Yes | 122019 | 6503 | 16654 | 1729 | 131436 |
| No | 355302 | 12748 | 30037 | 3222 | 348558 |
| I am completely deaf | 130 | 9 | 5 | 1 | 130 |
| Do not know | 19761 | 1068 | 2664 | 279 | 18022 |
| Prefer not to answer | 514 | 6 | 80 | 1 | 498 |
| Total | 497726 | 20334 | 49440 | 5232 | 498644 |

Note: The derived hearing difficulty measure is not cumulative, as only a subset of the participants from Instance 0 was interviewed for Instances 1-3. 479,994 participants with valid responses (i.e., yes or no) for the derived variable were considered for further analysis.

**Table 2**: Prevalence of hearing difficulty in noise (data field: 2257) across the UK Biobank sample. The hearing difficulty in noise measure was derived using the highest category from instances 0-3.

|  | Instance 0 (2006-10) | Instance 1 (2012-13) | Instance 2  (2014+) | Instance 3  (2019+) | Derived hearing difficulty in noise |
| --- | --- | --- | --- | --- | --- |
| Yes | 184195 | 8676 | 22007 | 2286 | 193960 |
| No | 306543 | 11215 | 26221 | 2827 | 297717 |
| Do not know | 9953 | 428 | 1116 | 116 | 9059 |
| Prefer not to answer | 674 | 6 | 91 | 2 | 646 |
| Total | 501365 | 20325 | 49435 | 5231 | 501382 |

Note: The derived hearing difficulty in noise measure is not cumulative, as only a subset of the participants from Instance 0 was interviewed for Instances 1-3. 491677 participants with valid responses (i.e., yes or no) for the derived variable were considered for further analysis.

Tables 1 and 2 - Please note that we have noticed some discrepancies between the responses for hearing difficulty (data field: 2247) and hearing difficulty in noise (data field: 2257) across instances 0-3. For example, there were 2166, 7377, and 870 new occurrences of hearing difficulty in instances 1, 2, and 3, respectively, compared to instance 0. Additionally, about 1378, 3210, and 314 participants who reported hearing difficulty in instance 0 did not reveal any hearing difficulty during instances 1, 2, and 3. We believe that age, acute pathologies (such as acute otitis media), and inaccurate responses could be the factors underlying the discrepancies in the responses across the instances. We did not exclude any participants based on the variability of responses across instances 0-3. Audiological data does not have a high enough resolution to differentiate between inaccurate (random) versus accurate (driven by aging and acute pathologies) responses.

**Figure 1:** A schematic diagram showing the subject selection criteria and their influence on the sample size.


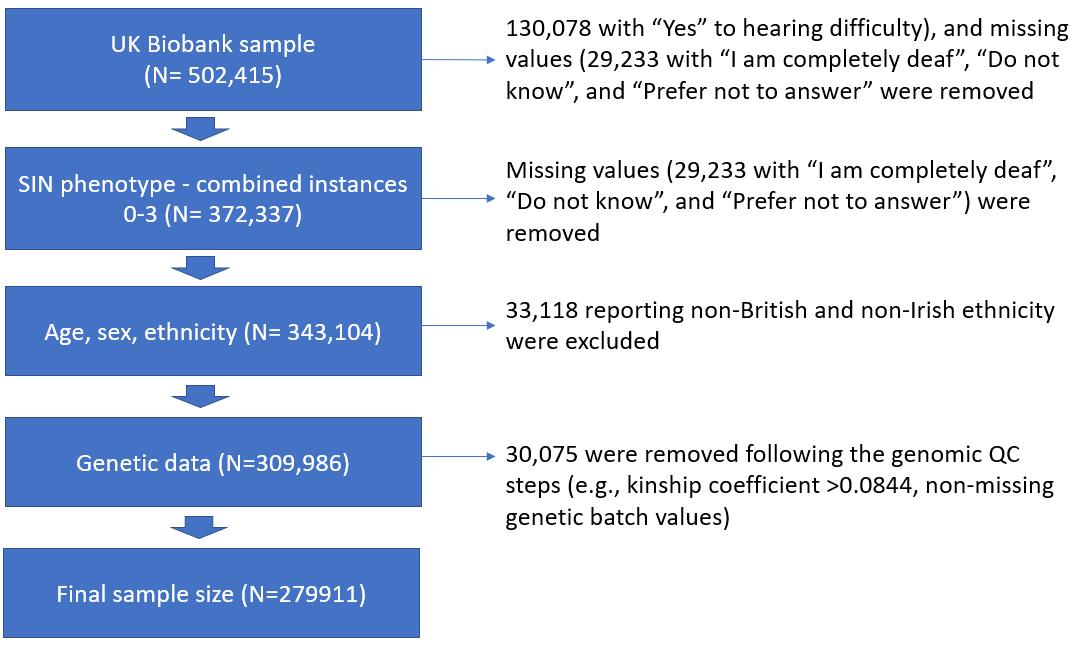


**Figure 2:** LocusZoom results

Figure 2.1: [6: 27,556,141 A/T](https://my.locuszoom.org/gwas/774370/region/?chrom=6&start=27306141&end=27806141)


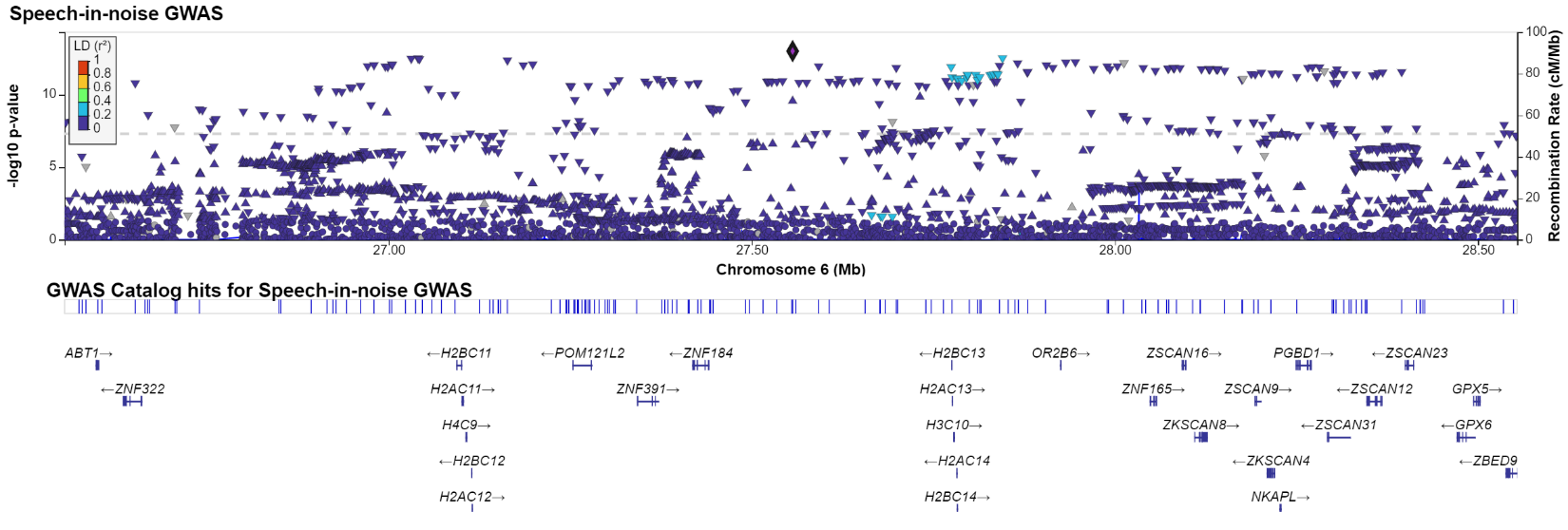


Figure 2.2: [6: 32,571,350 G/C](https://my.locuszoom.org/gwas/774370/region/?chrom=6&start=32321350&end=32821350)
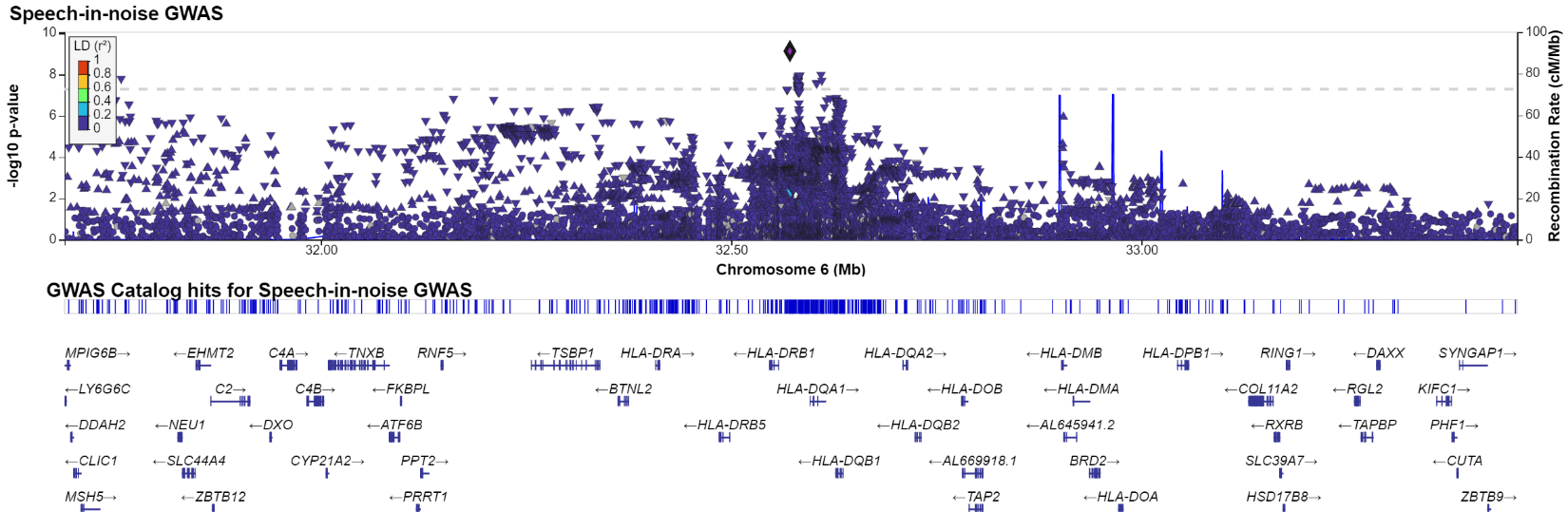


Figure 2.3: [7: 86,253,638 C/T](https://my.locuszoom.org/gwas/774370/region/?chrom=7&start=86003638&end=86503638)
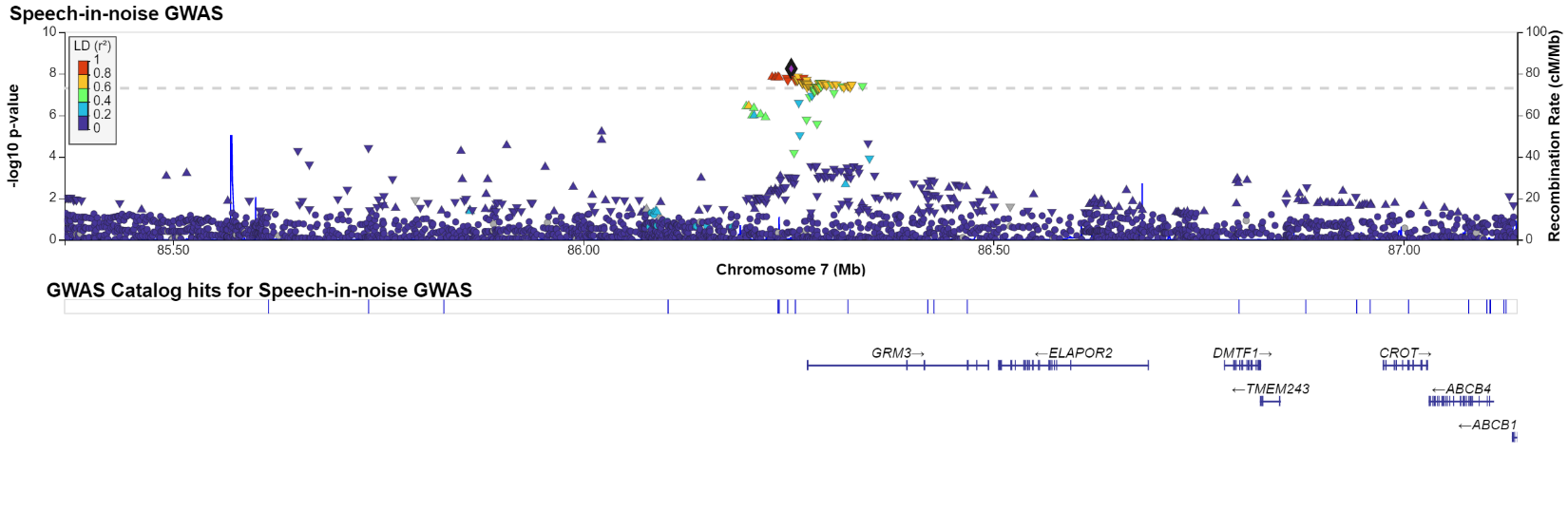


Figure 2.4: [17: 44,065,263 T/C](https://my.locuszoom.org/gwas/774370/region/?chrom=17&start=43815263&end=44315263)
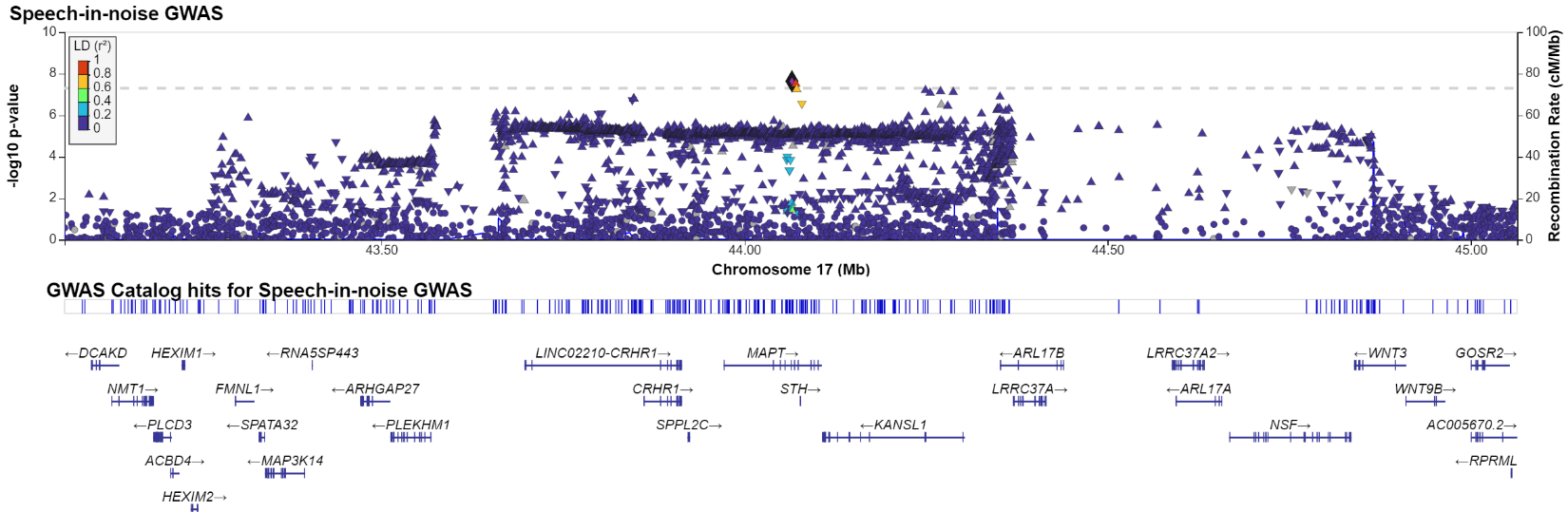


Figure 2.5: [3: 6,189,009 T/C](https://my.locuszoom.org/gwas/774370/region/?chrom=3&start=5939009&end=6439009)
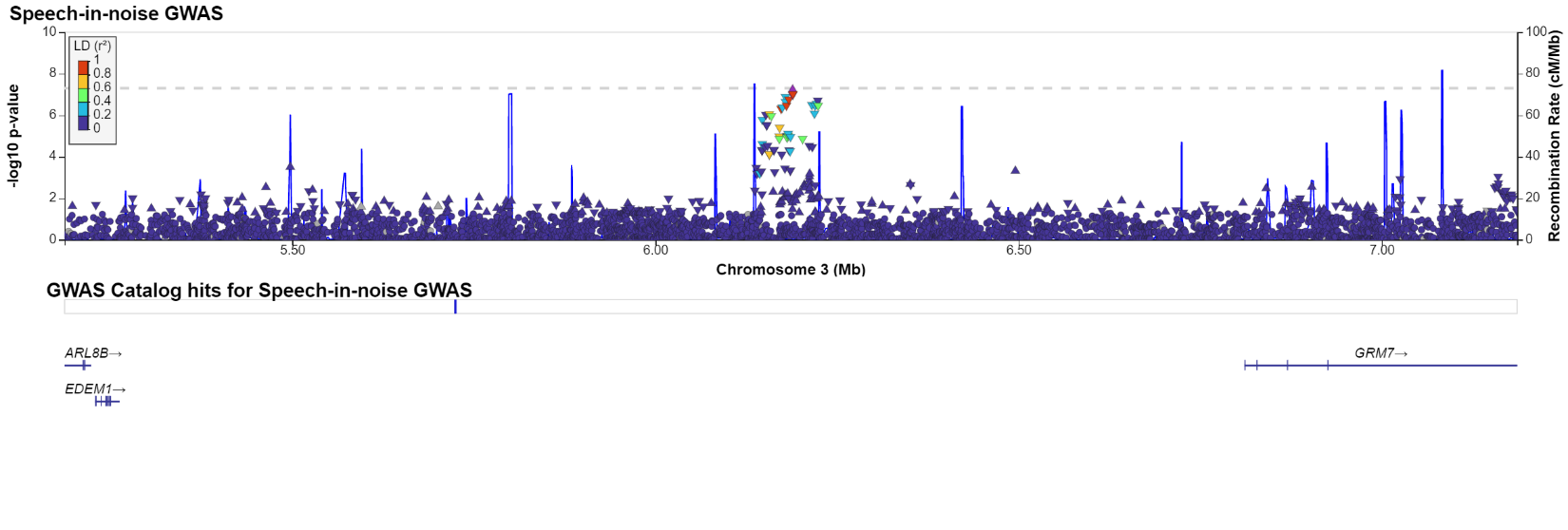


Figure 2.6: [6: 32,161,366 T/C](https://my.locuszoom.org/gwas/774370/region/?chrom=6&start=31911366&end=32411366)
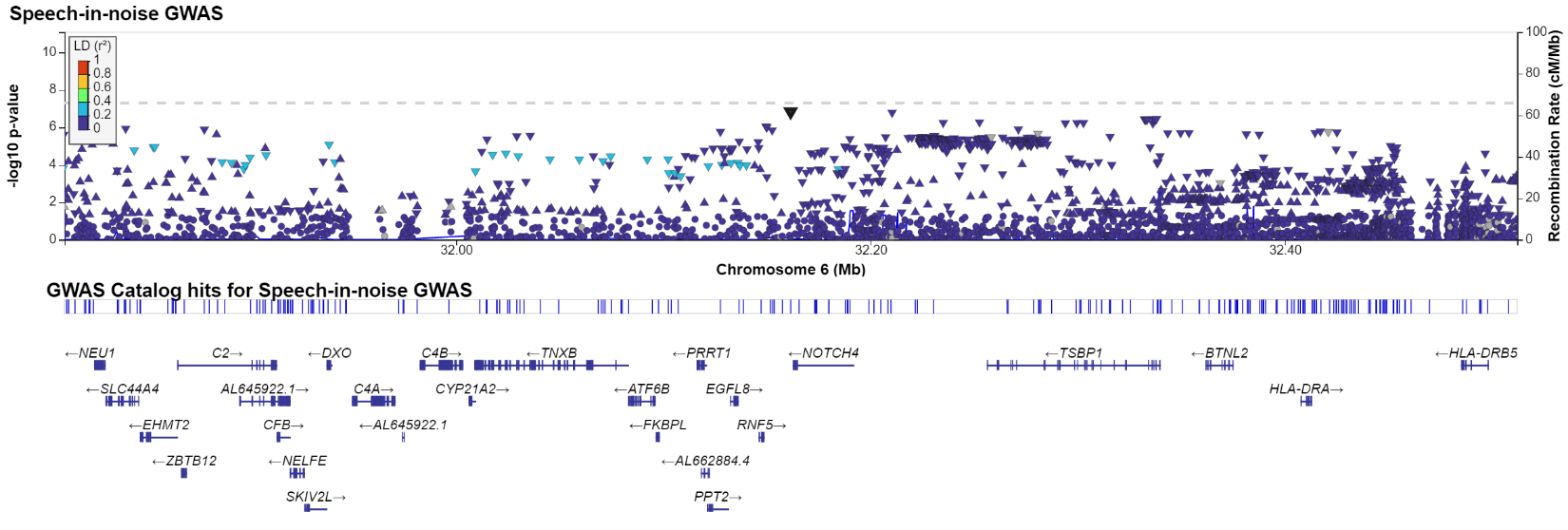


Figure 2.7: [1: 190,291,562 G/A](https://my.locuszoom.org/gwas/774370/region/?chrom=1&start=190041562&end=190541562)
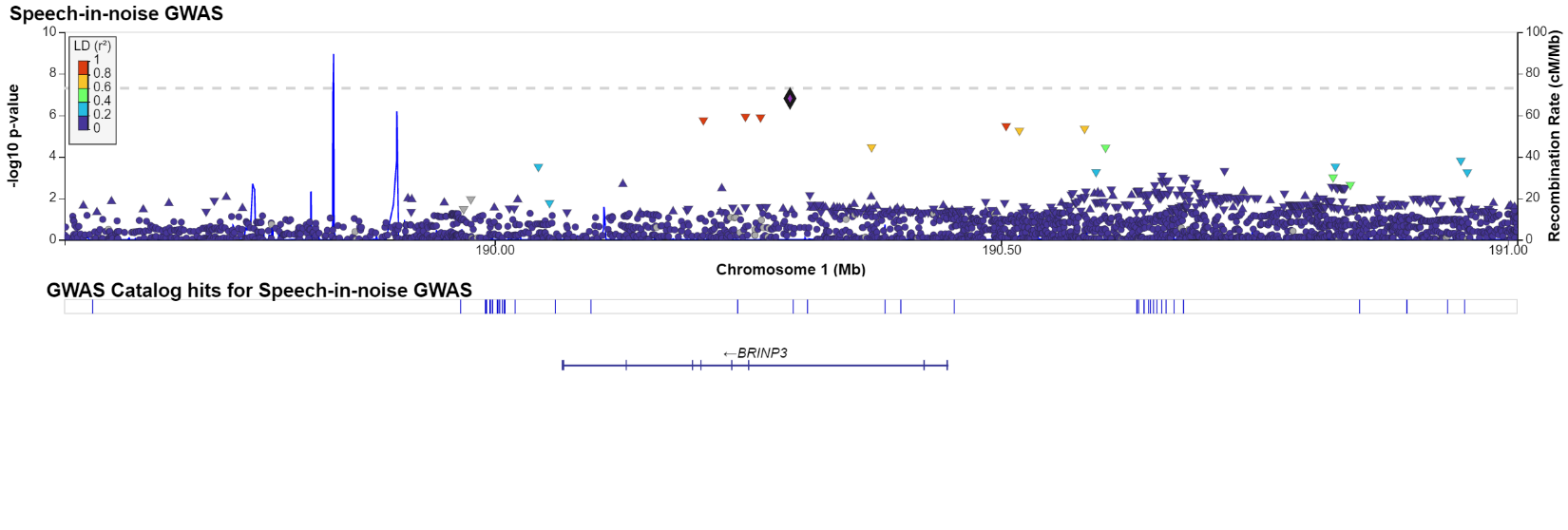


Figure 2.8: [17: 43,847,569 TTTTGTTTG/T](https://my.locuszoom.org/gwas/774370/region/?chrom=17&start=43597569&end=44097569)


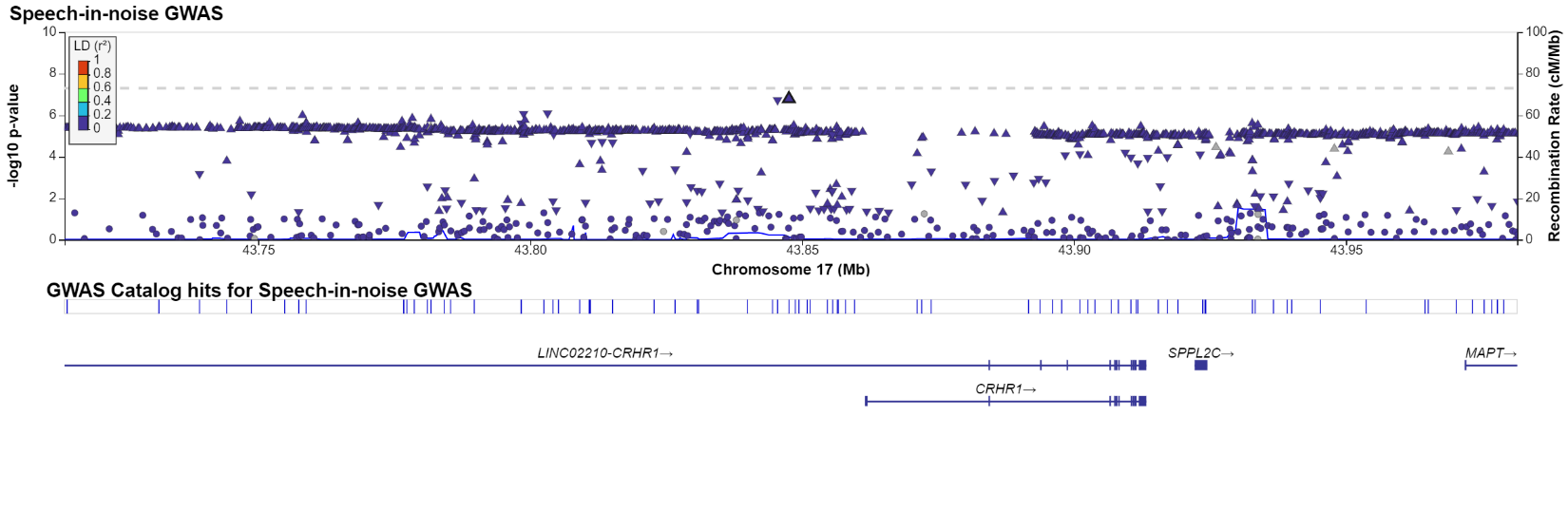


Figure 2.9: [17: 2,555,592 C/T](https://my.locuszoom.org/gwas/774370/region/?chrom=17&start=2305592&end=2805592)


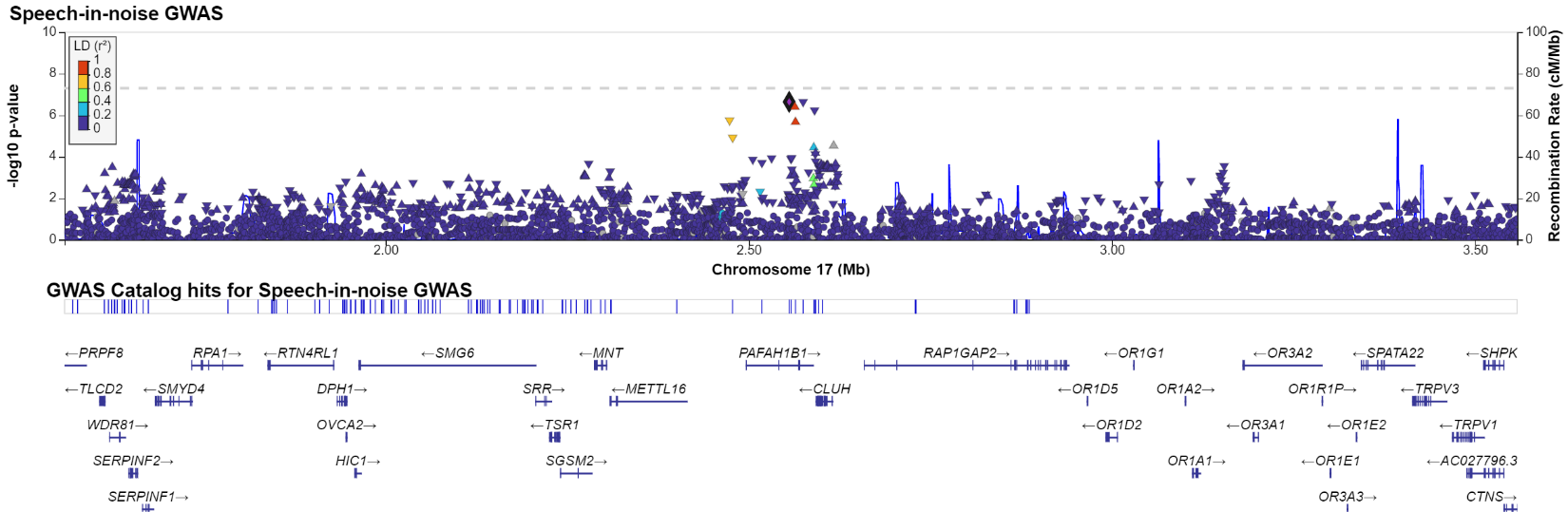


Figure 2.10: [11: 97,179,965 G/A](https://my.locuszoom.org/gwas/774370/region/?chrom=11&start=96929965&end=97429965)


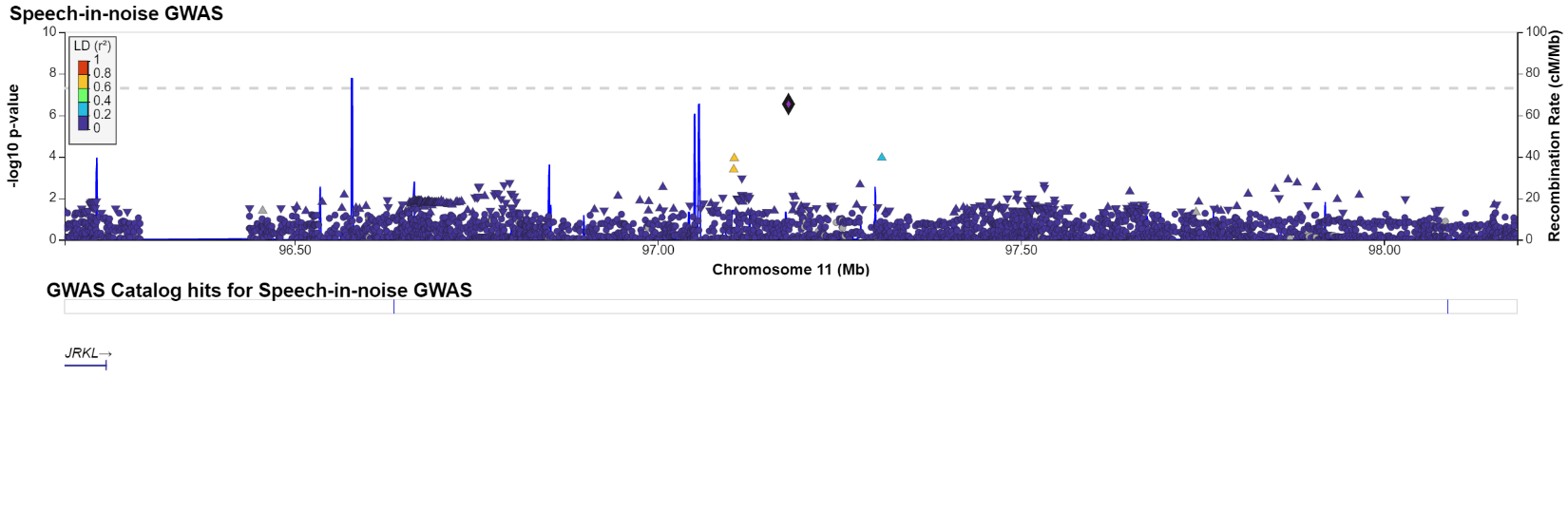


Figure 2.11: [7: 36,189,045 T/C](https://my.locuszoom.org/gwas/774370/region/?chrom=7&start=35939045&end=36439045)


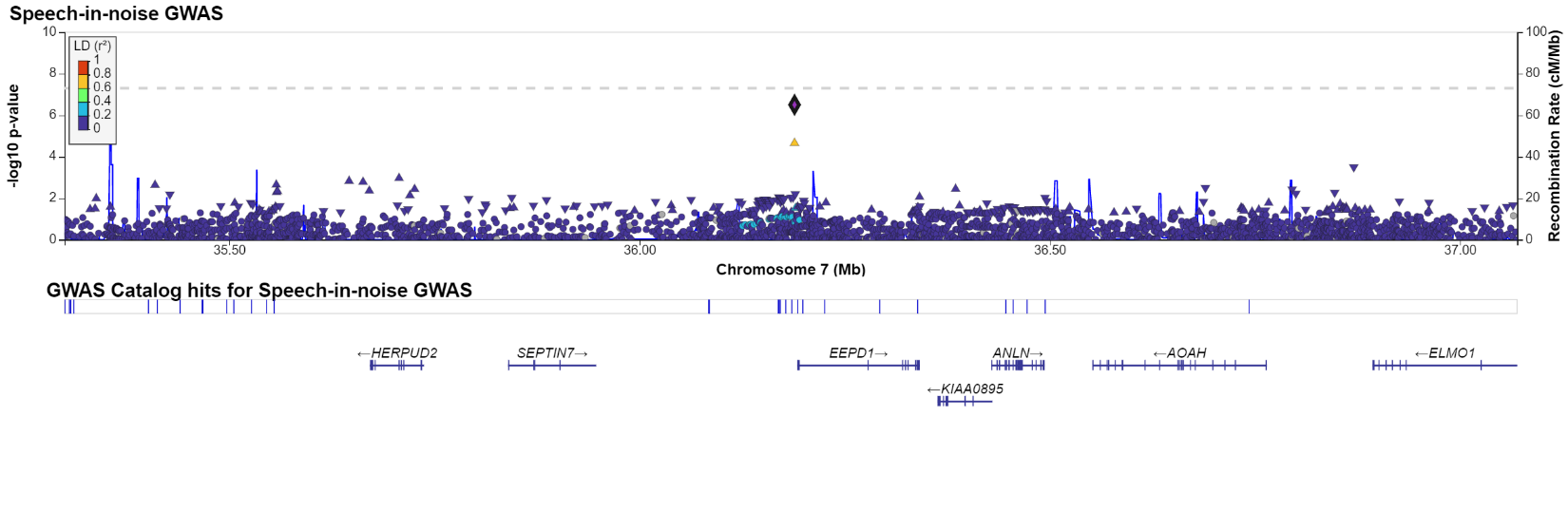


Figure 2.12: [1: 71,725,353 G/GTATATTATATATAATATATATAATA](https://my.locuszoom.org/gwas/774370/region/?chrom=1&start=71475353&end=71975353)


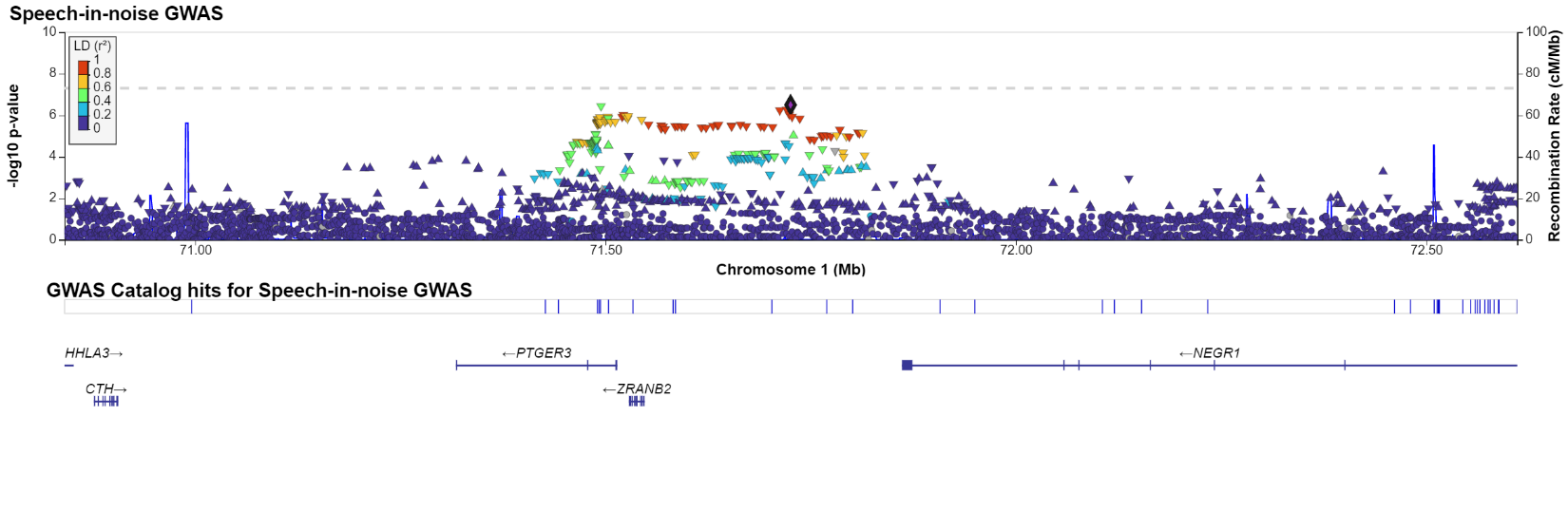


Figure 2.13: [12: 125,630,997 C/A](https://my.locuszoom.org/gwas/774370/region/?chrom=12&start=125380997&end=125880997)


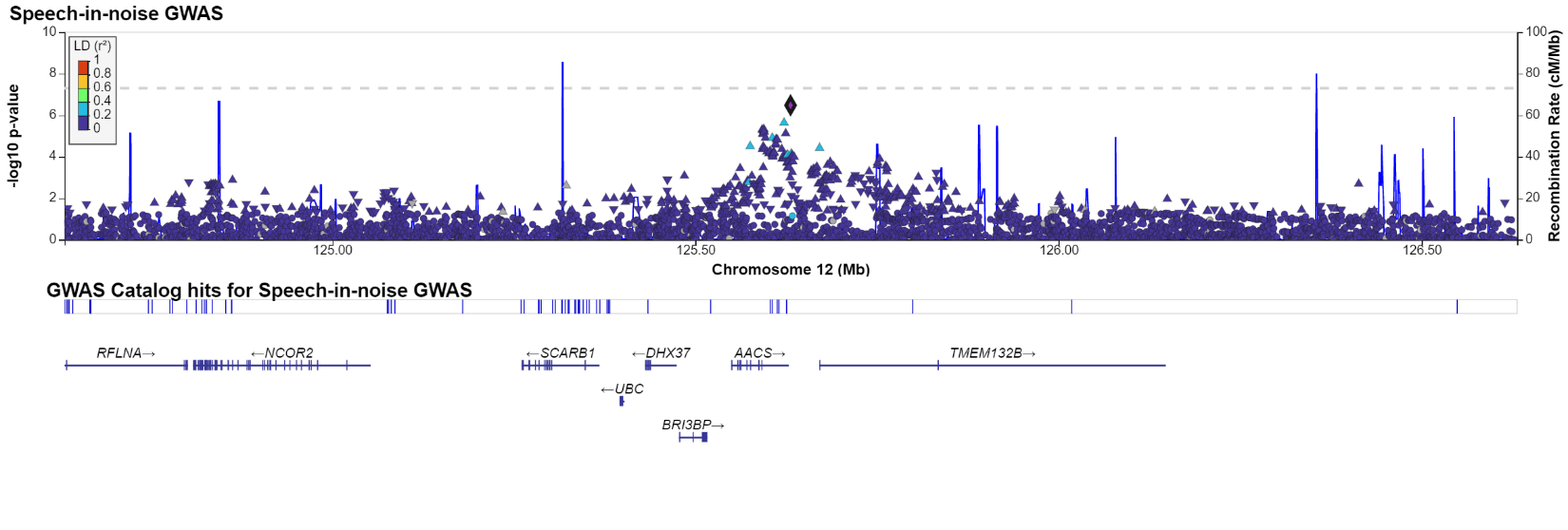


Figure 2.14: [11: 47,306,630 C/T](https://my.locuszoom.org/gwas/774370/region/?chrom=11&start=47056630&end=47556630)


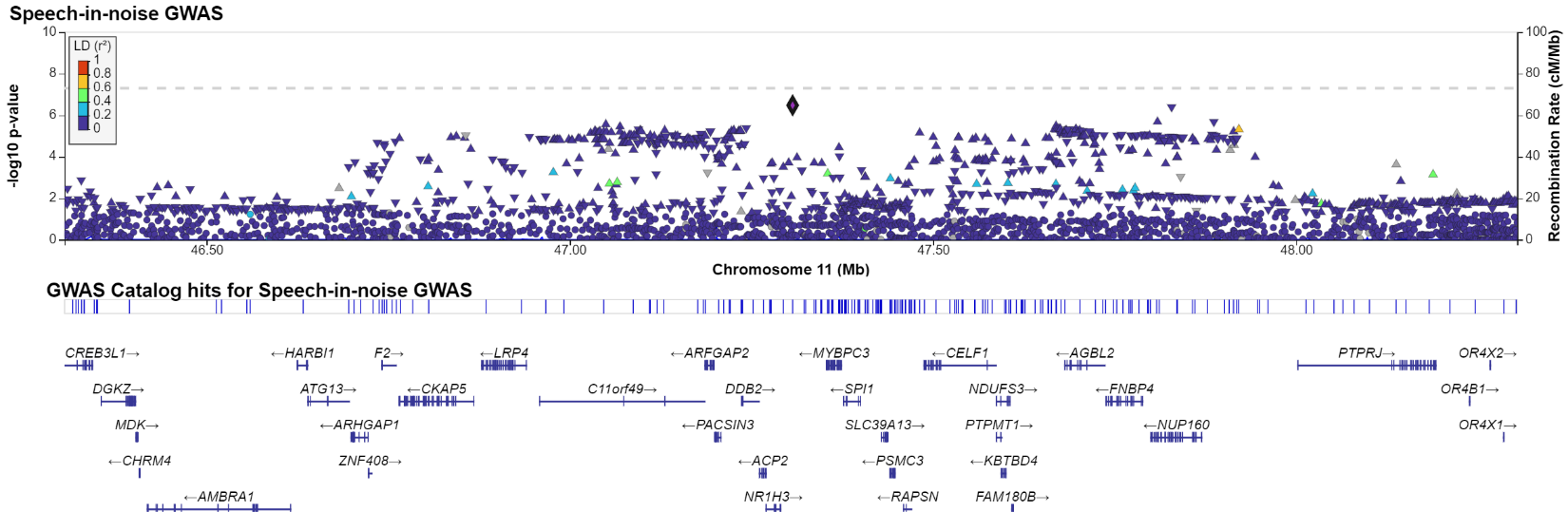


Figure 2.15: [17: 42,779,633 G/GGCCATCTCA](https://my.locuszoom.org/gwas/774370/region/?chrom=17&start=42529633&end=43029633)


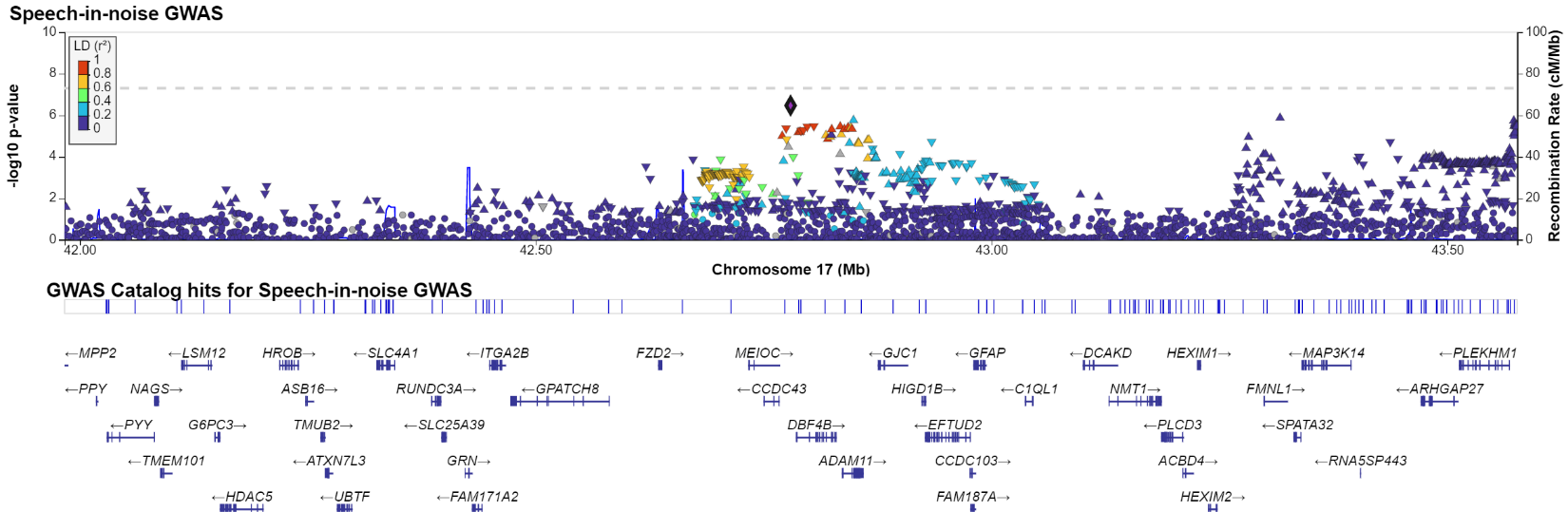


Figure 2.16: 1[1: 47,828,404 C/T](https://my.locuszoom.org/gwas/774370/region/?chrom=11&start=47578404&end=48078404)


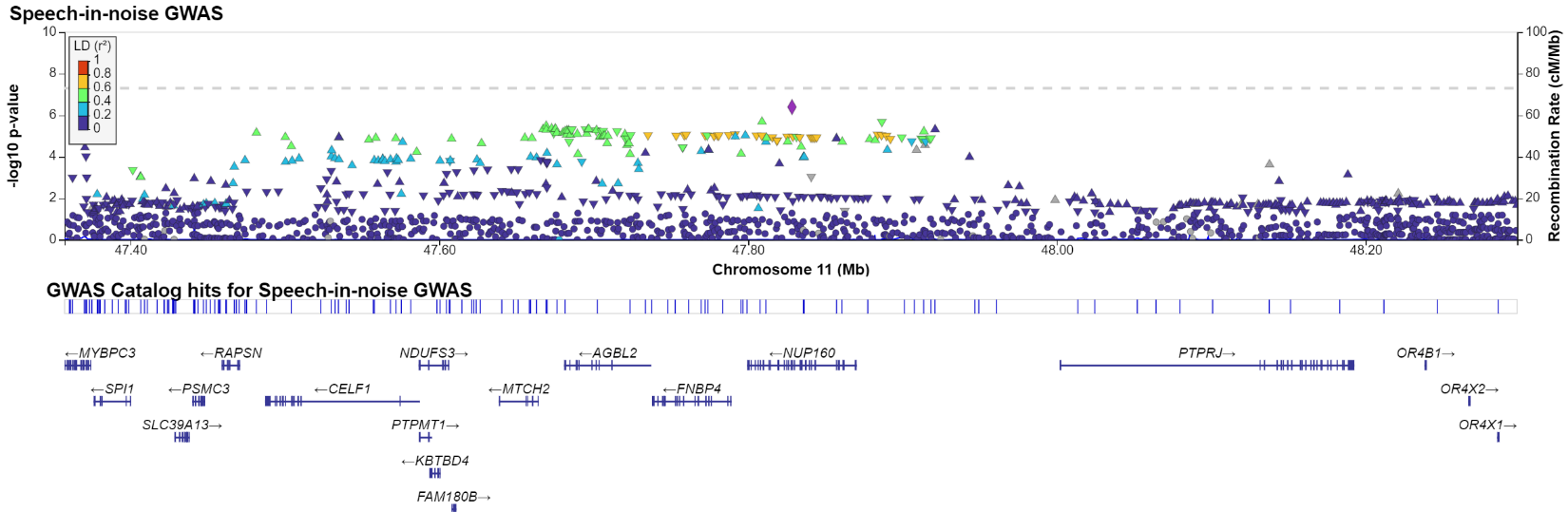


Figure 2.17: [9: 80,661,256 TC/T](https://my.locuszoom.org/gwas/774370/region/?chrom=9&start=80411256&end=80911256)


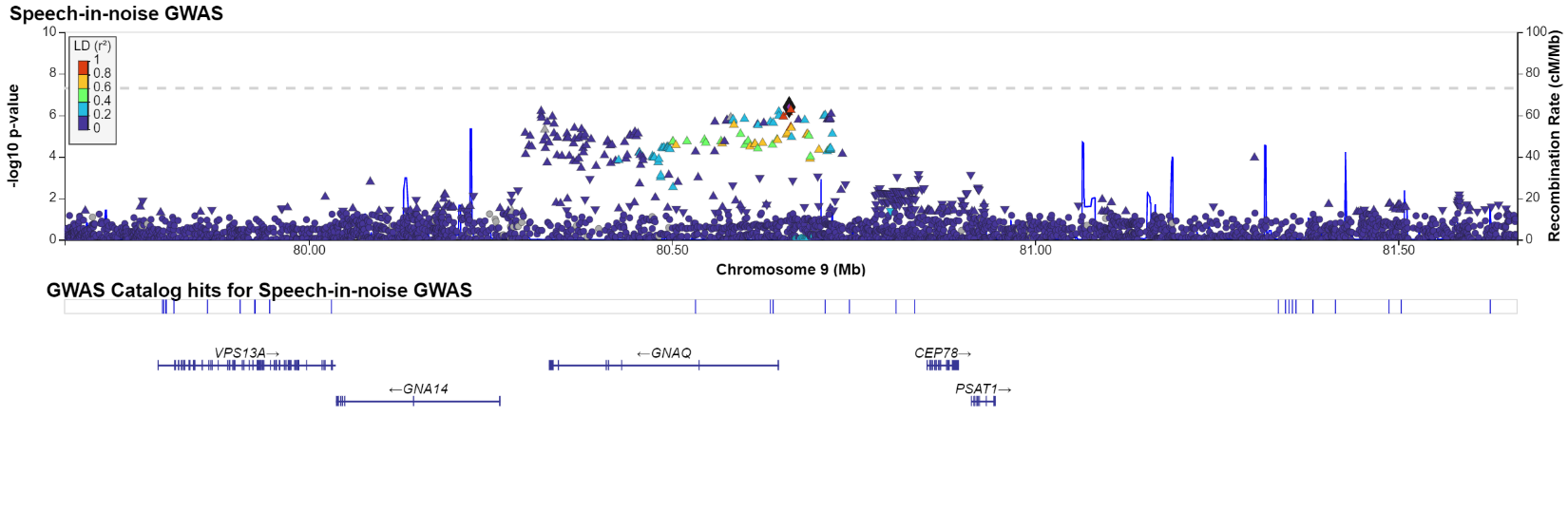


Figure 2.18: [6: 25,191,039 G/C](https://my.locuszoom.org/gwas/774370/region/?chrom=6&start=24941039&end=25441039)


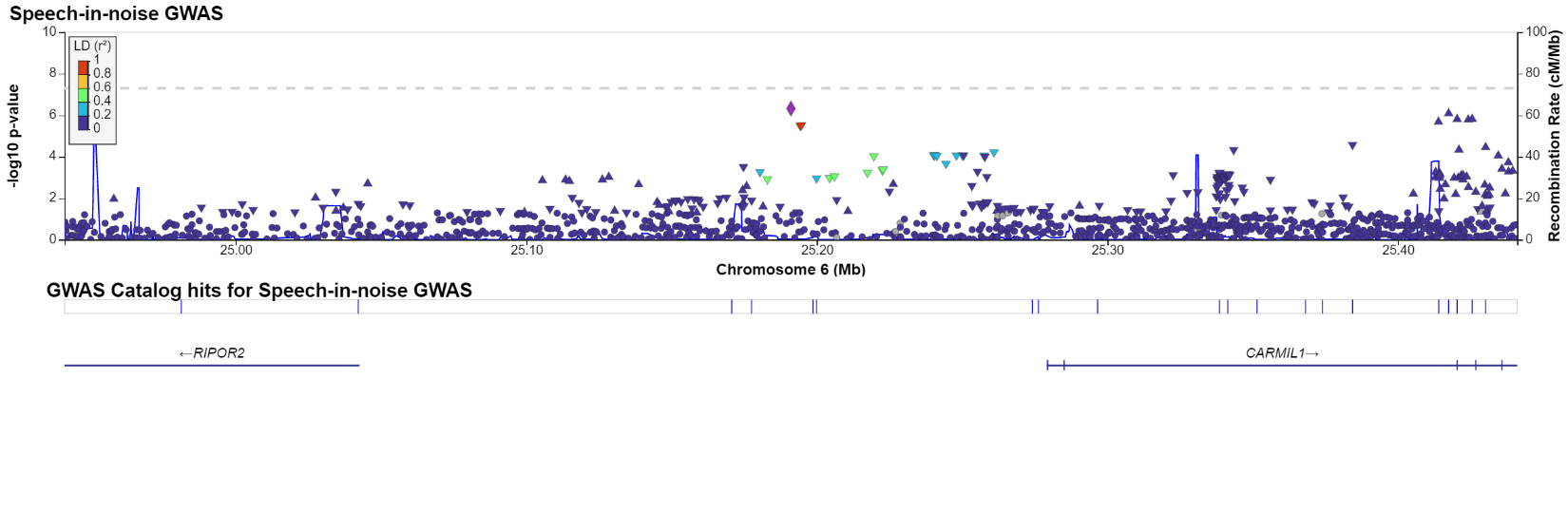


Figure 2.19: [9: 80,319,769 C/CTT](https://my.locuszoom.org/gwas/774370/region/?chrom=9&start=80069769&end=80569769)


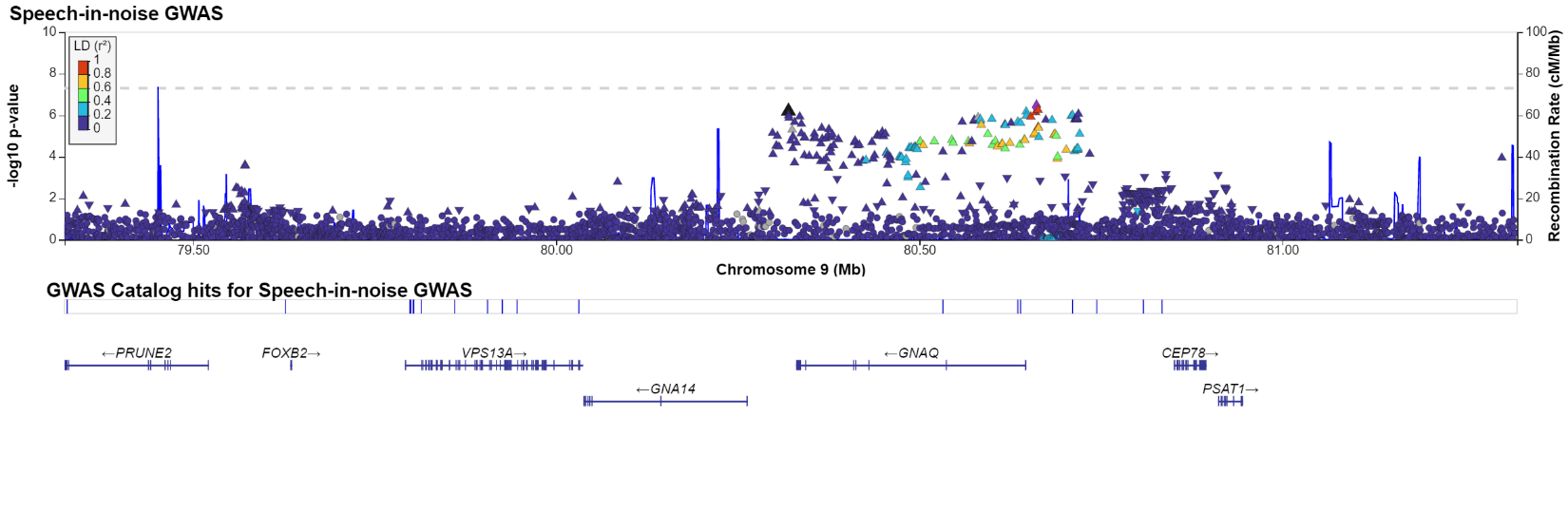


Figure 2.20: [5: 22,309,022 AGAGAGAG/A](https://my.locuszoom.org/gwas/774370/region/?chrom=5&start=22059022&end=22559022)


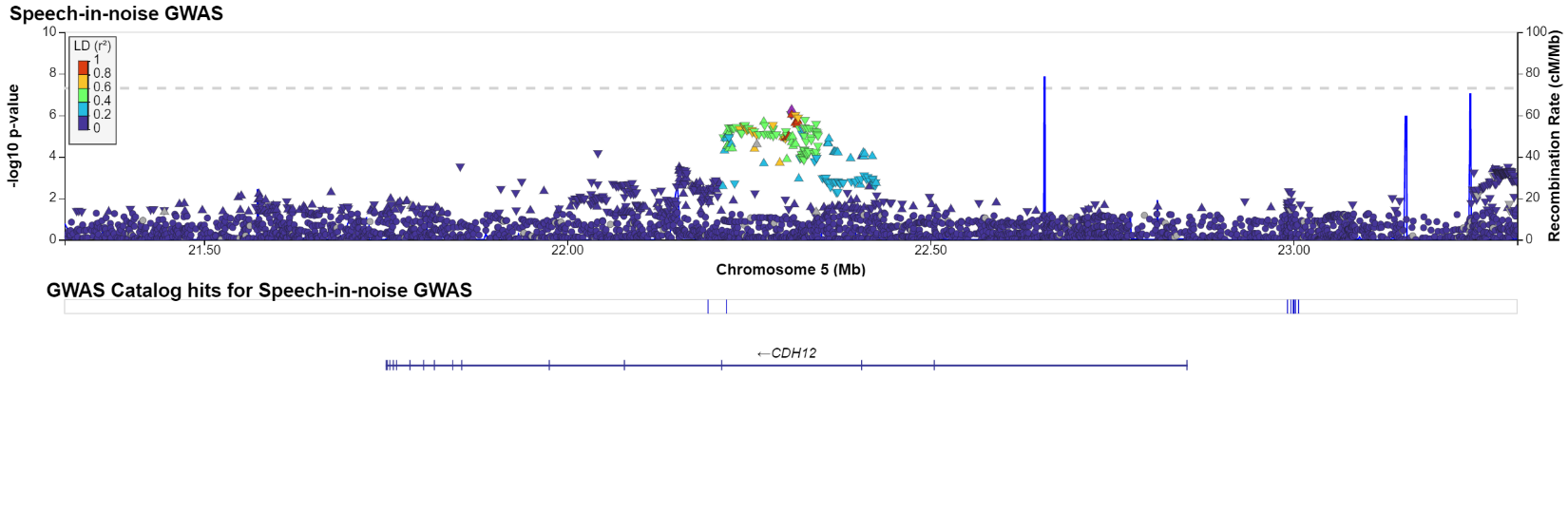


Figure 2.21: [2: 81,408,132 A/G](https://my.locuszoom.org/gwas/774370/region/?chrom=2&start=81158132&end=81658132)


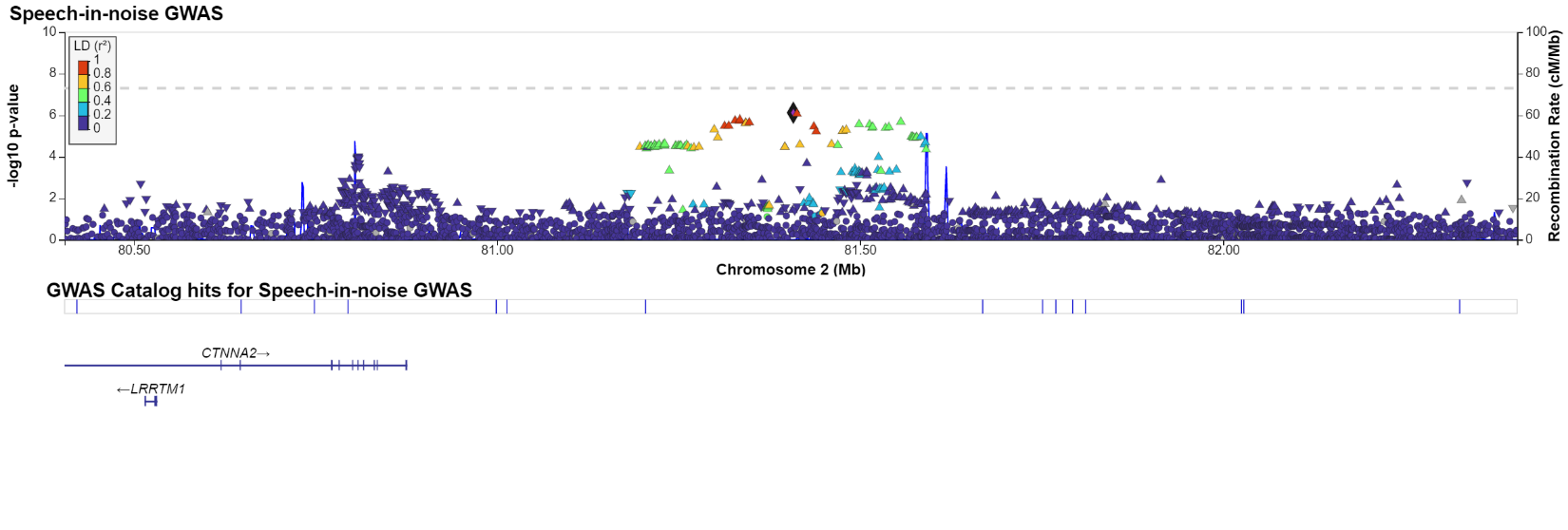


Figure 2.22: [9: 32,428,841 C/CA](https://my.locuszoom.org/gwas/774370/region/?chrom=9&start=32178841&end=32678841)


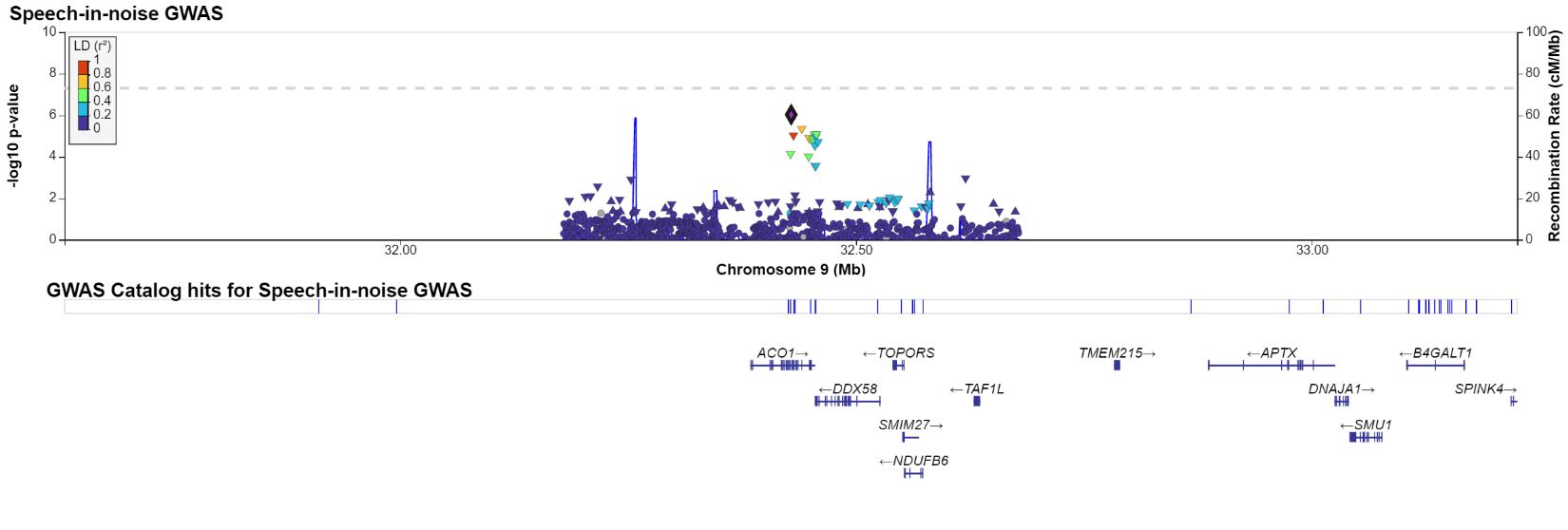


Figure 2.23: [1: 163,763,429 GGT/G](https://my.locuszoom.org/gwas/774370/region/?chrom=1&start=163513429&end=164013429)


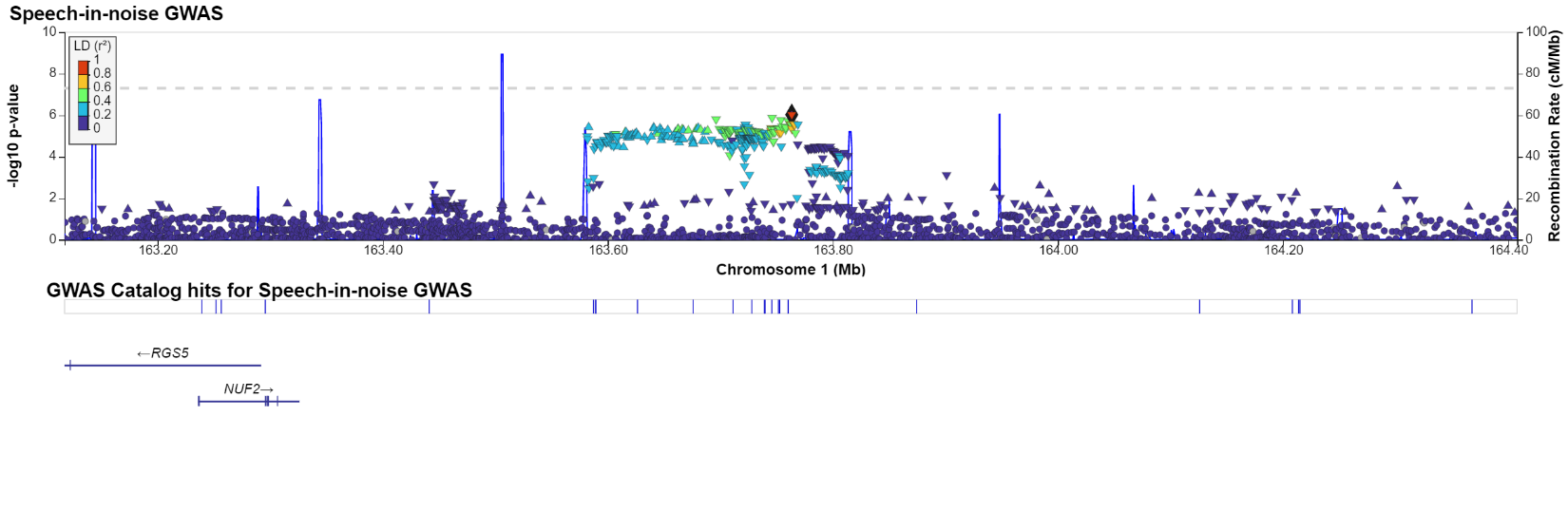


Figure 2.24: [6: 100,929,516 C/T](https://my.locuszoom.org/gwas/774370/region/?chrom=6&start=100679516&end=101179516)


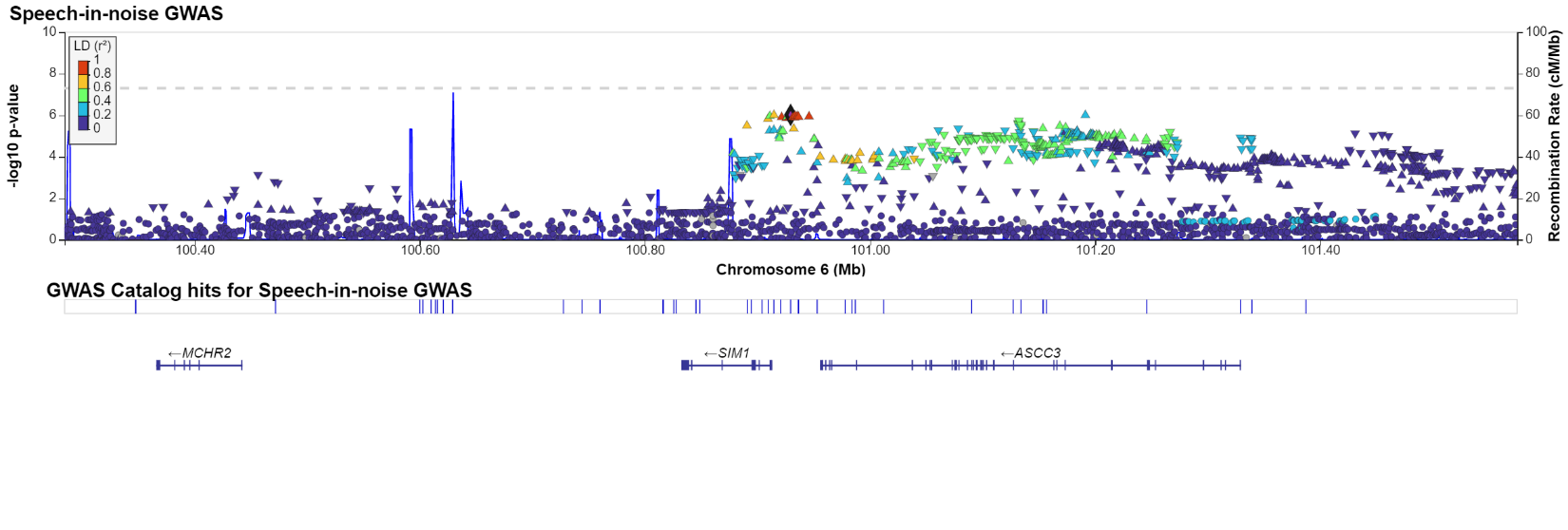


Figure 2.25: [6: 101,191,397 C/T](https://my.locuszoom.org/gwas/774370/region/?chrom=6&start=100941397&end=101441397)


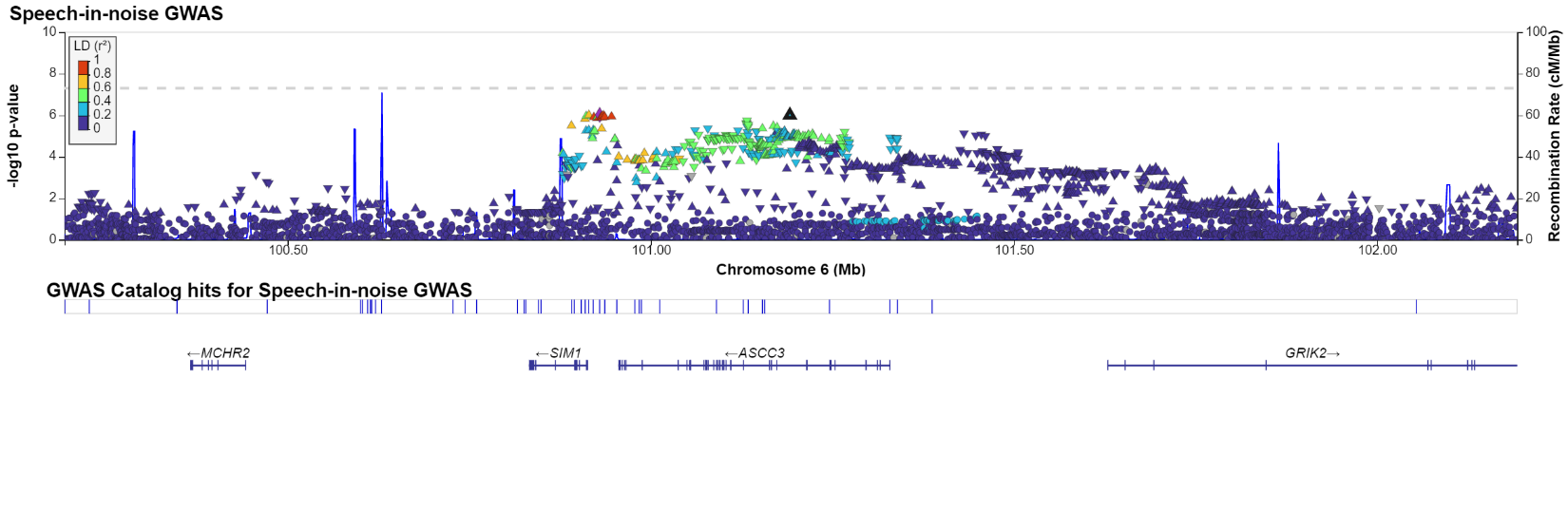


**Figure 3: Manhattan plot: Gene-based test (FUMA) – SIN phenotype**


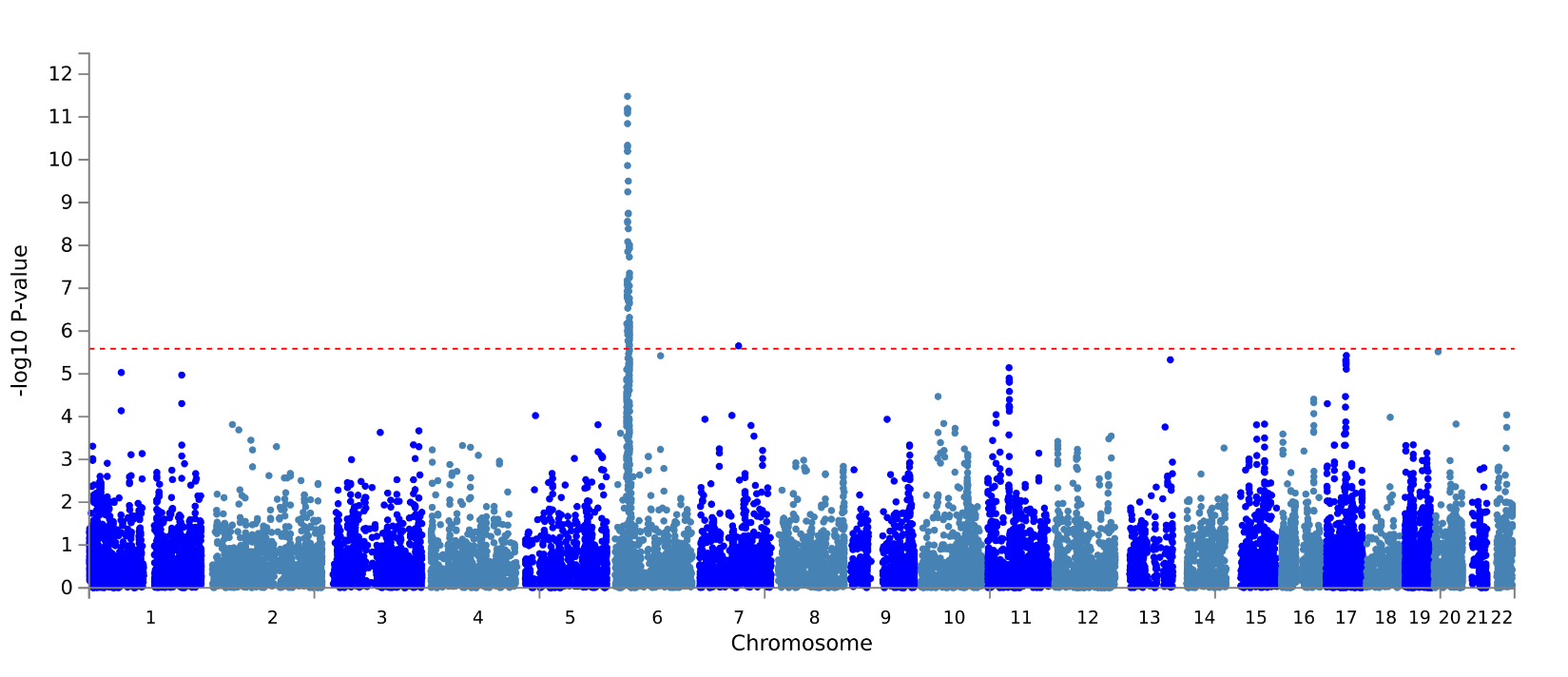


**Figure 4: MAGMA Tissue expression analysis**

**Figure 4.1: GTEx v8 30 general tissue types**


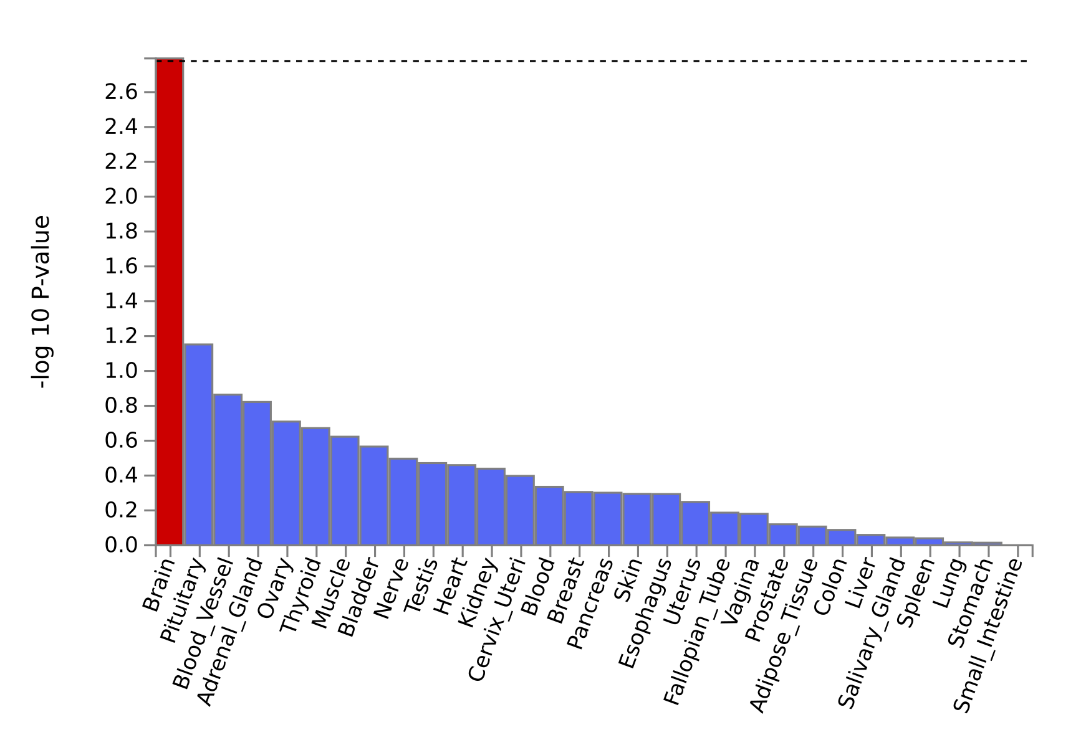


**Figure 4.2: GTEx v8 53 tissue types**


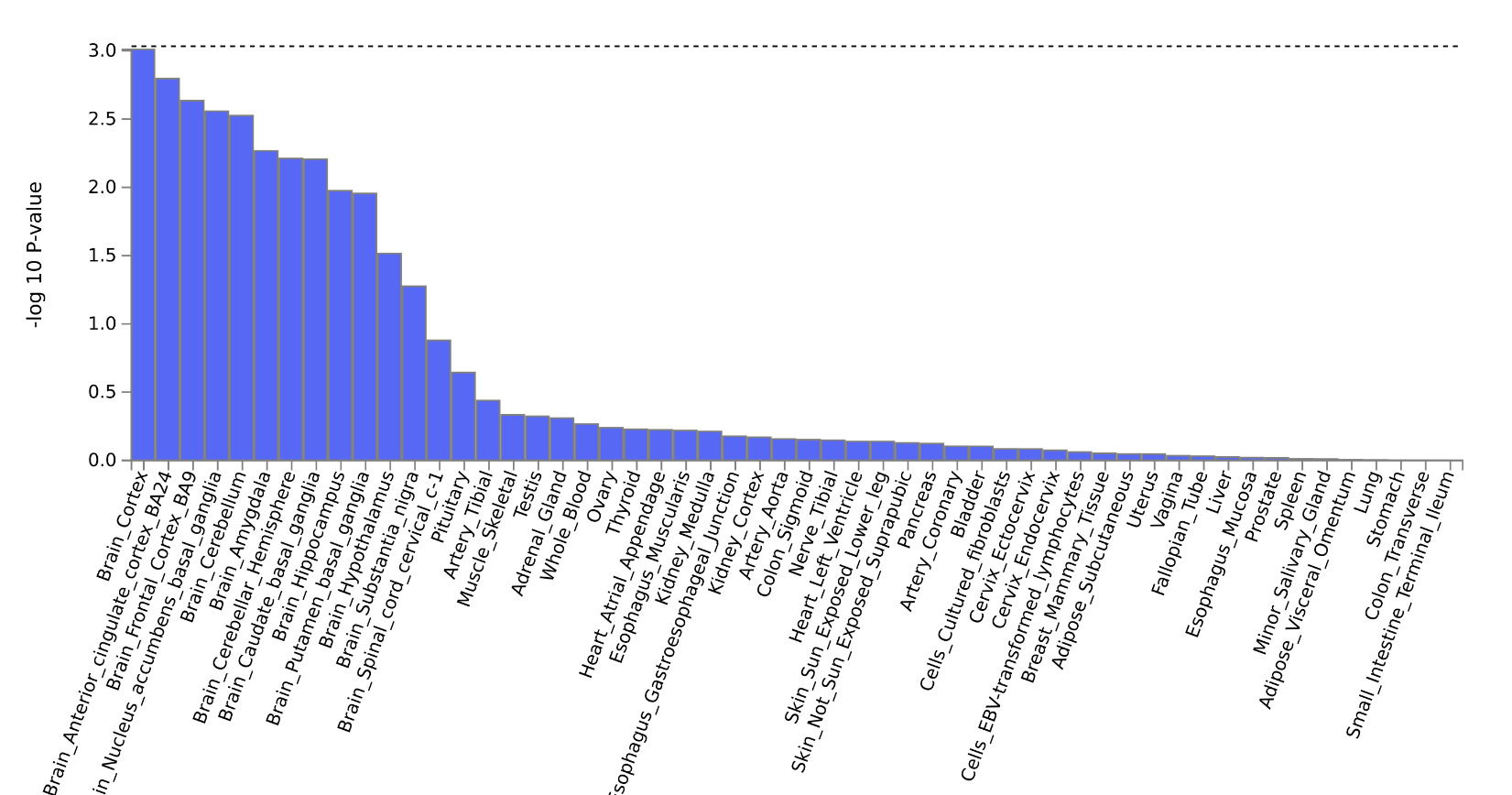


**Figure 5:** Quantile-Quantile (Q-Q) plots of expected and observed *p*-values (converted on a -log10 (*p*-value) scale) for the genome-wide association study of the SIN phenotype. The plot on the right-hand side shows Q-Q plots for minor allele frequency-based categories and genomic inflation measurement (GC lambda).


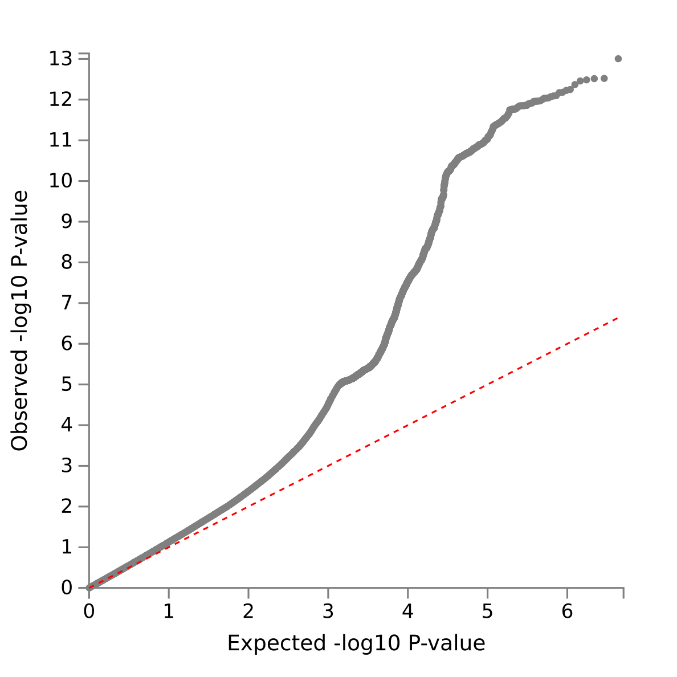

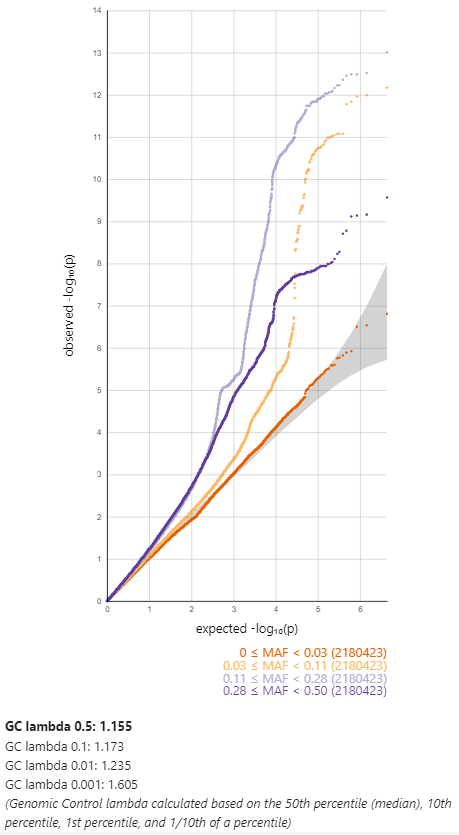


**Figure 6:** Results of the enrichment analysis: SIN phenotype

**Figure 6.1**: Top results of the MAGMA gene-set analysis

| **Gene Set** | **N genes** | **Beta** | **Beta STD** | **SE** | **P** | **Pbon** |
| --- | --- | --- | --- | --- | --- | --- |
| Curated_gene_sets:biocarta_tubby_pathway | 4 | 2.3935 | 0.034496 | 0.48352 | 3.75E-07 | 0.005* |
| GO_bp:go_neurological_system_process_involved_in_regulation_of_systemic_arterial_blood_pressure | 9 | 0.92954 | 0.020093 | 0.25055 | 0.000104 | 1 |
| Curated_gene_sets:kommagani_tp63_gamma_targets | 9 | 0.94923 | 0.020518 | 0.25745 | 0.000114 | 1 |
| GO_bp:go_positive_regulation_of_myoblast_differentiation | 22 | 0.7941 | 0.026828 | 0.21792 | 0.000135 | 1 |
| GO_cc:go_synapse | 1119 | 0.095567 | 0.02236 | 0.026427 | 0.00015 | 1 |
| GO_bp:go_genetic_imprinting | 26 | 0.59459 | 0.021835 | 0.16682 | 0.000183 | 1 |
| Curated_gene_sets:white_neuroblastoma_with_1p36.3_deletion | 19 | 1.0902 | 0.03423 | 0.31674 | 0.00029 | 1 |
| GO_cc:go_extrinsic_component_of_postsynaptic_specialization_membrane | 5 | 1.334 | 0.021495 | 0.38866 | 0.0003 | 1 |
| GO_cc:go_synapse_part | 892 | 0.10035 | 0.021093 | 0.029467 | 0.000331 | 1 |
| GO_bp:go_regulation_of_locomotion_involved_in_locomotory_behavior | 5 | 1.2354 | 0.019906 | 0.36683 | 0.00038 | 1 |

**Figure 6.2**: Positional gene-set analysis


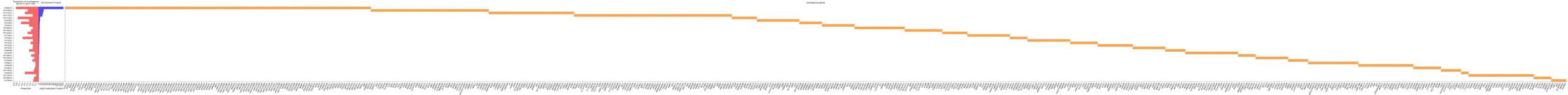


**Figure 6.3**: Curated gene-set analysis


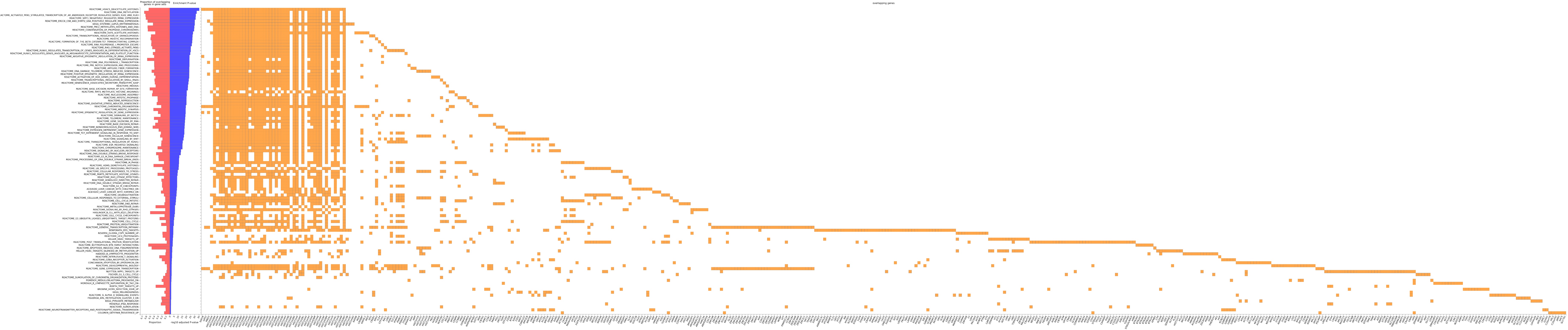


**Figure 6.4**: Chemical and genetic perturbation gene sets (MsigDB c2)


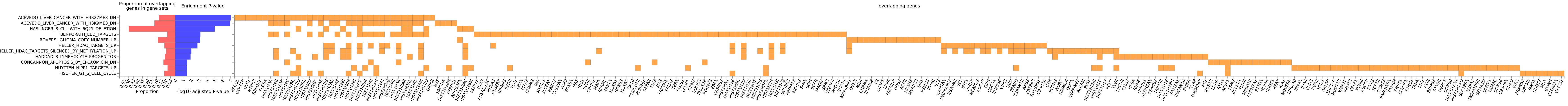


**Figure 6.5**: All Canonical Pathways (MsigDB c2)


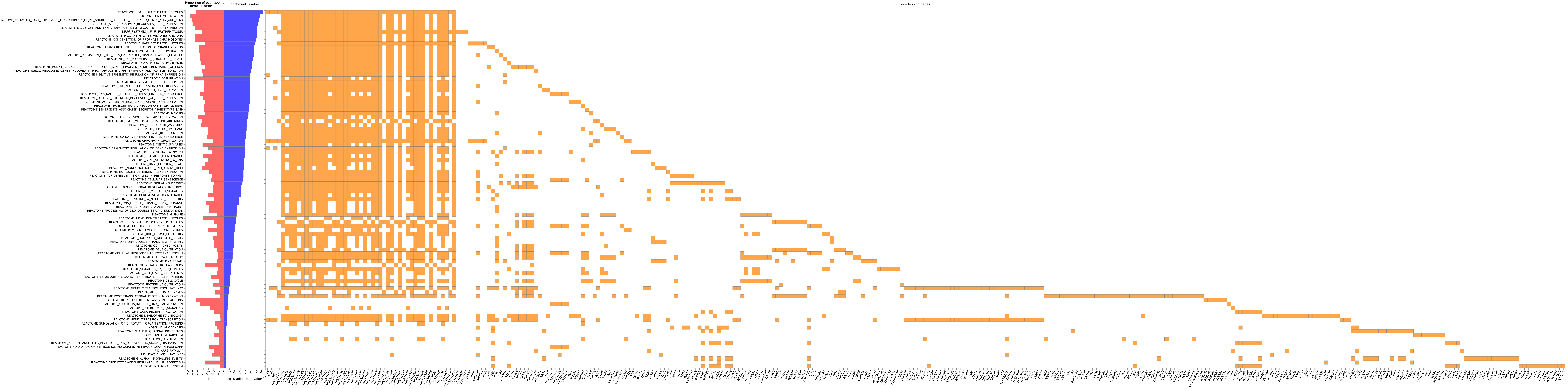


**Figure 6.6**: KEGG gene-set analysis (MsigDB c2)


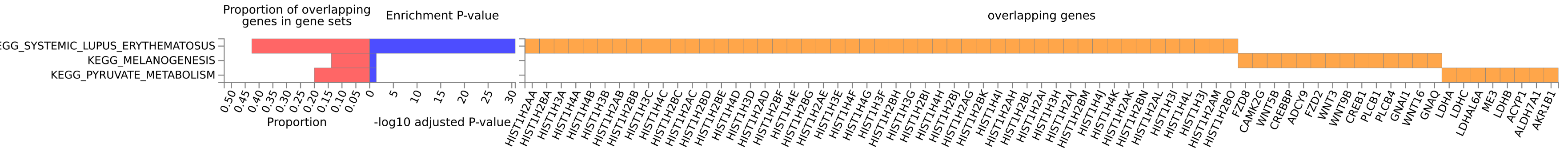


**Figure 6.7: TF-targets (MsigDB c4)**


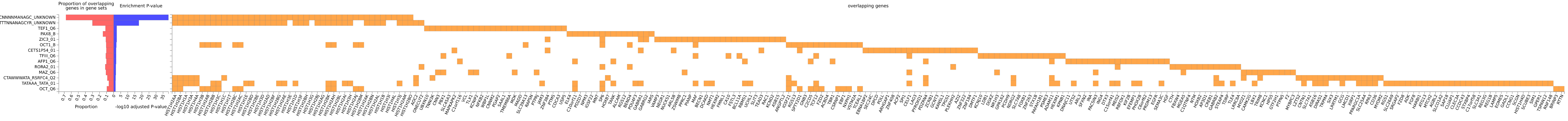


**Figure 6.8:** All computational gene sets (MsigDB c4)


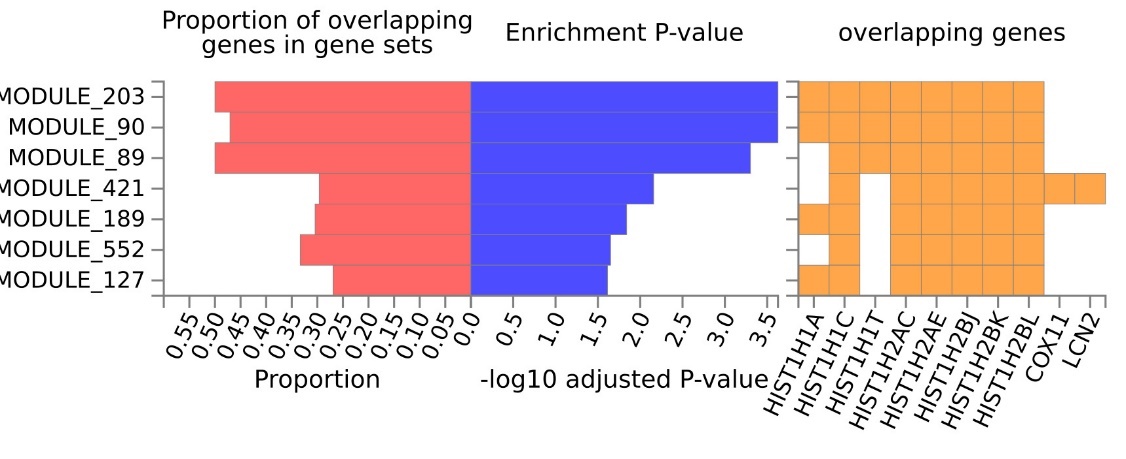


**Figure 6.9**: Cancer gene modules (MsigDB c4)


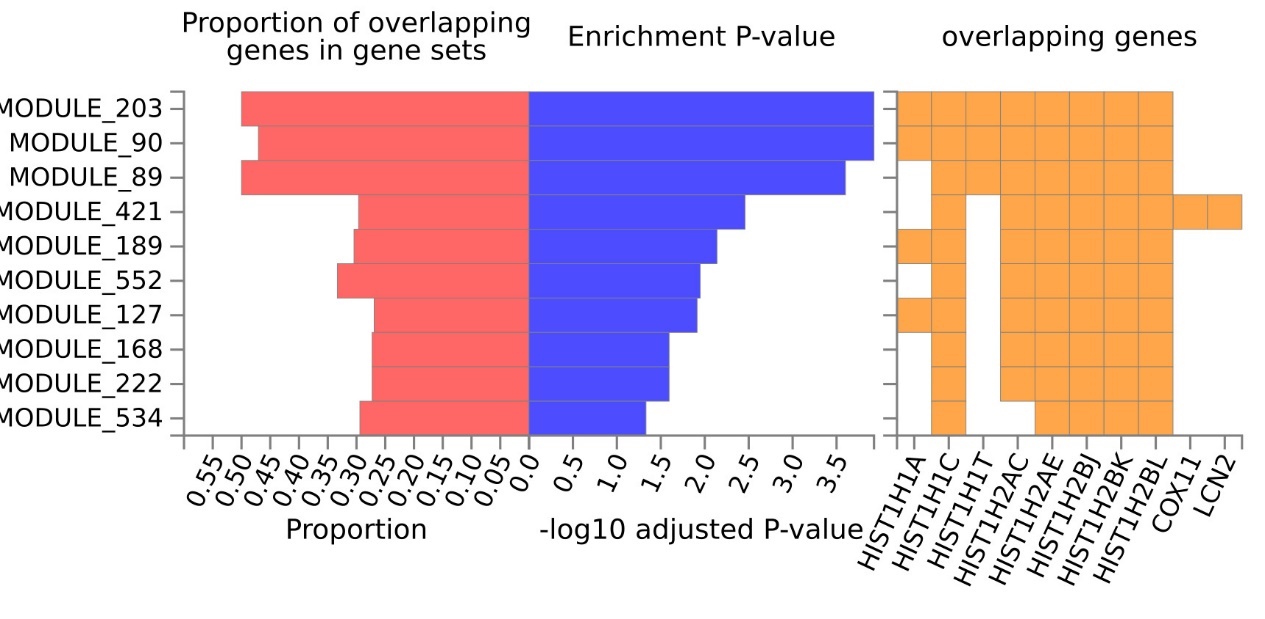


**Figure 6.10**: Reactome gene-set analysis (MsigDB c2)


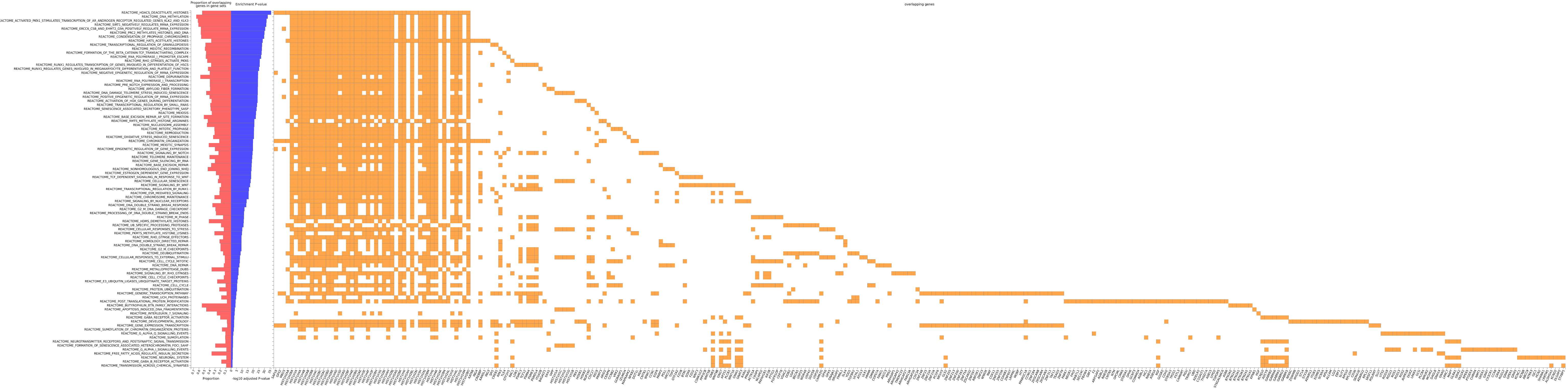


**Figure 6.11**: GO biological processes (MsigDB c5)


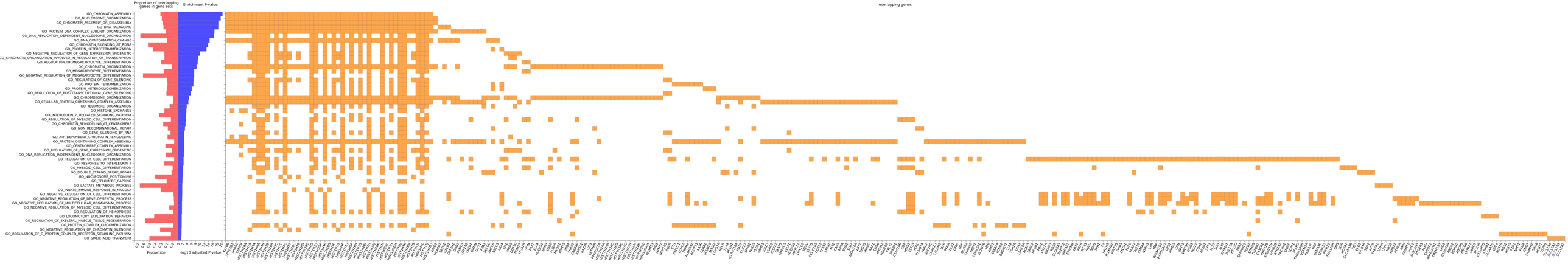


**Figure 6.12**: GO cellular components (MsigDB c5)


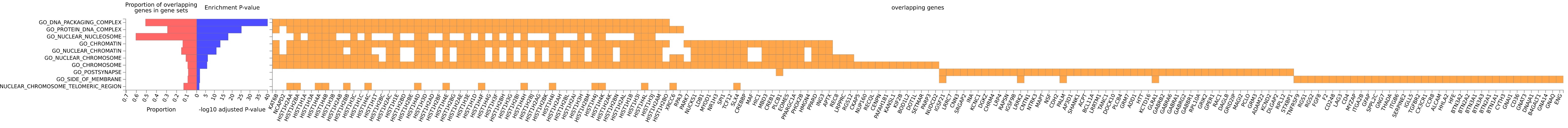


**Figure 6.13**: GO molecular functions (MsigDB c5)


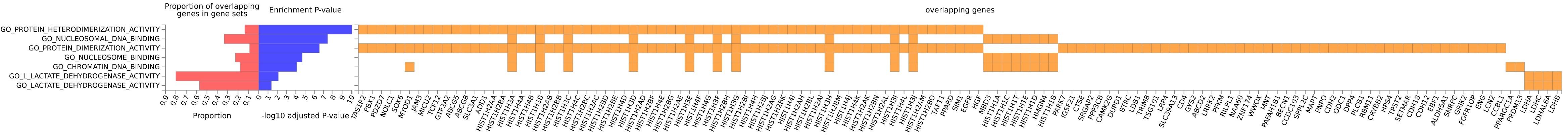


**Figure 6.14**: Oncogenic signatures (MsigDB c6)


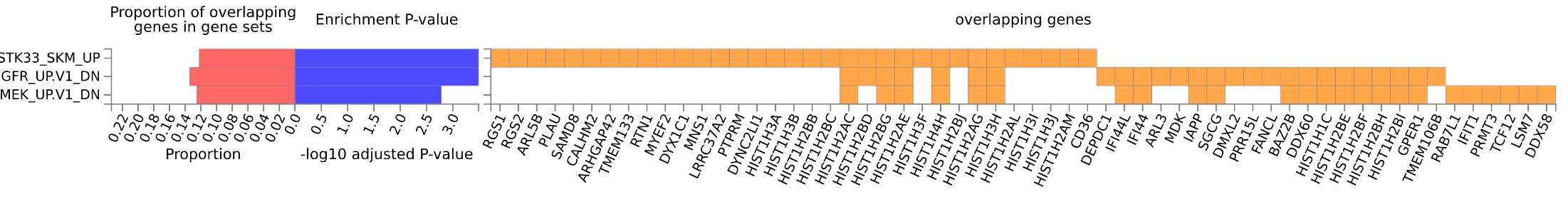


**Figure 6.15**: Immunologic signatures (MsigDB c6)


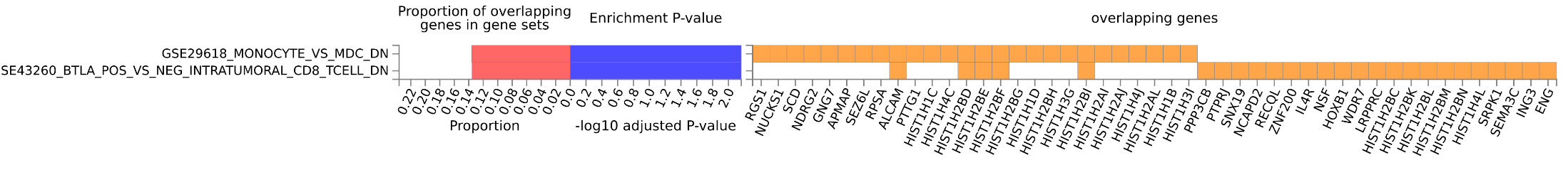


**Figure 6.16**: WikiPathways


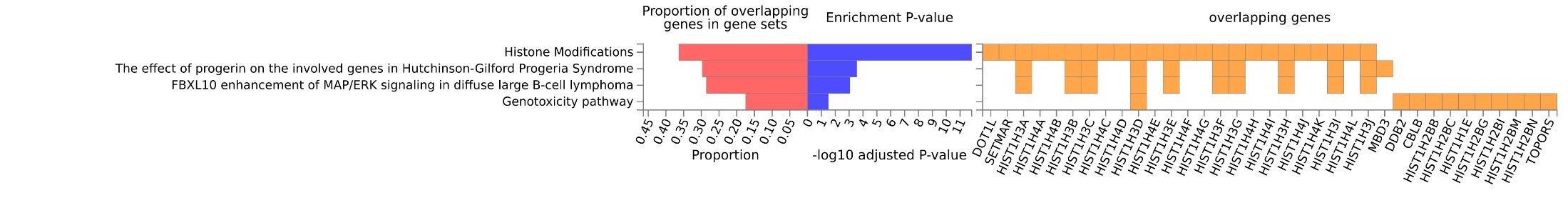


**Figure 6.17:** GWAS catalog reported gene set analysis


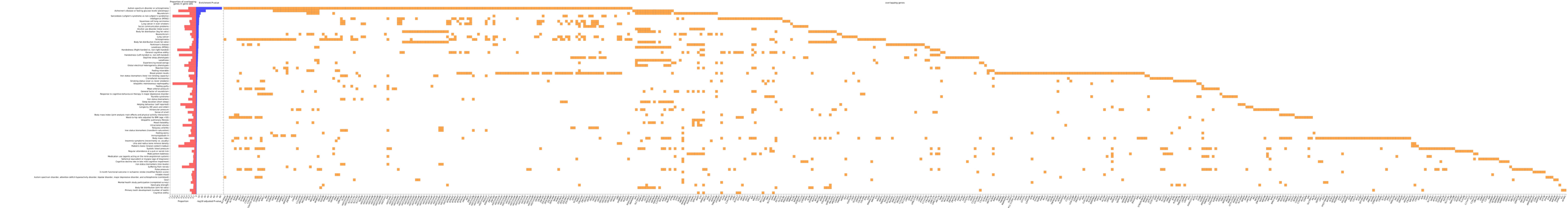


**Figure 6.18:** Cell-type specific gene set enrichment analysis for human prefrontal cortex (Fuma cell type 7672)


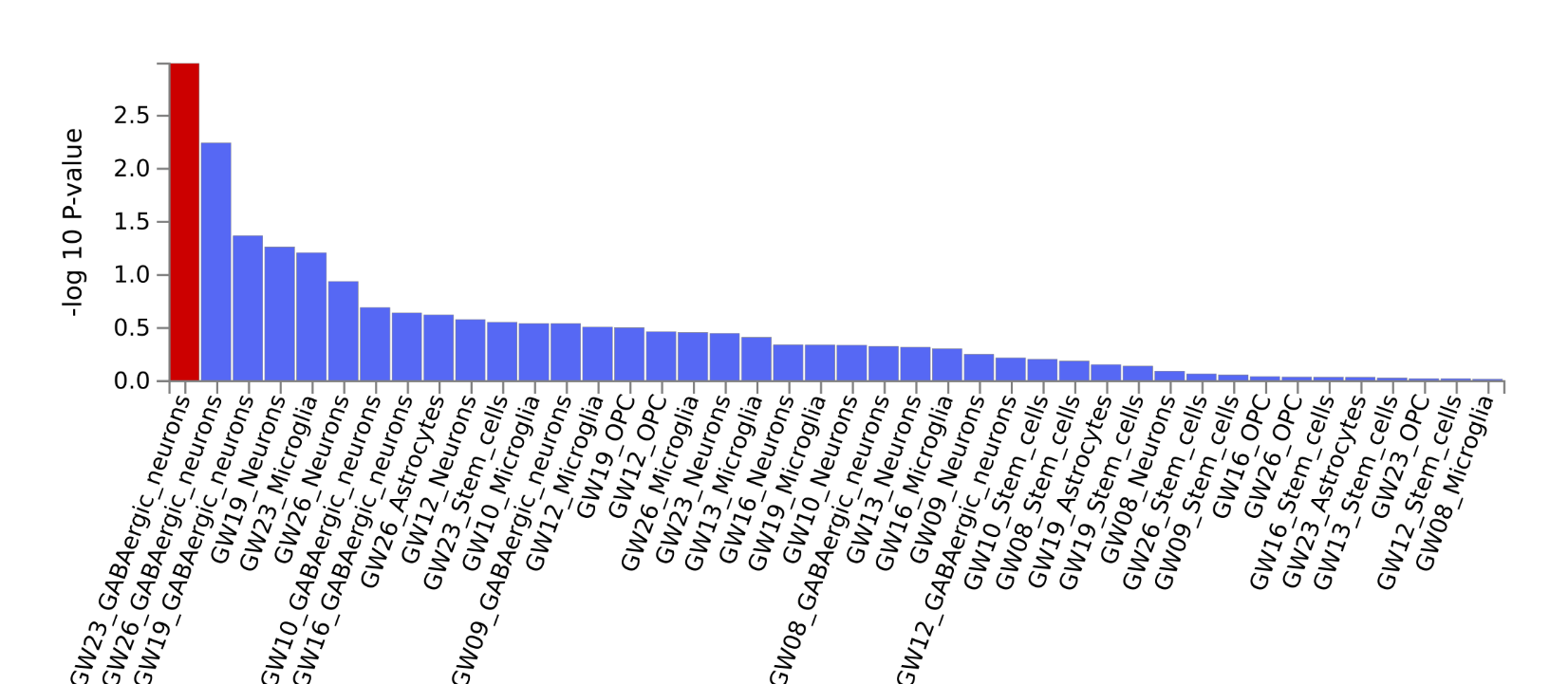


**Figure 7**: Results of the phenome-wide association analysis for the lead SNPs using LocusZoom.


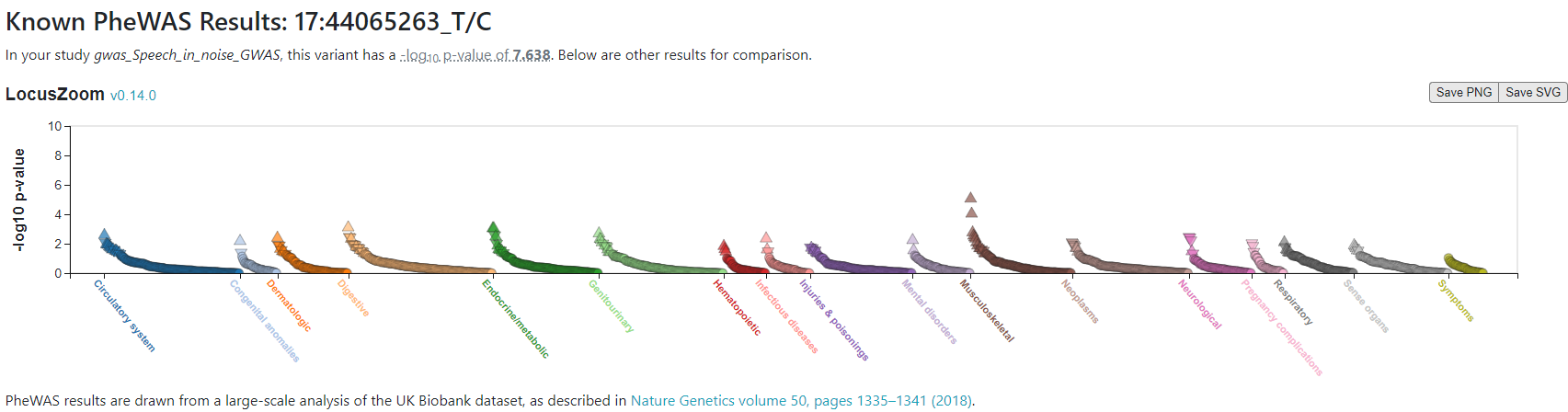


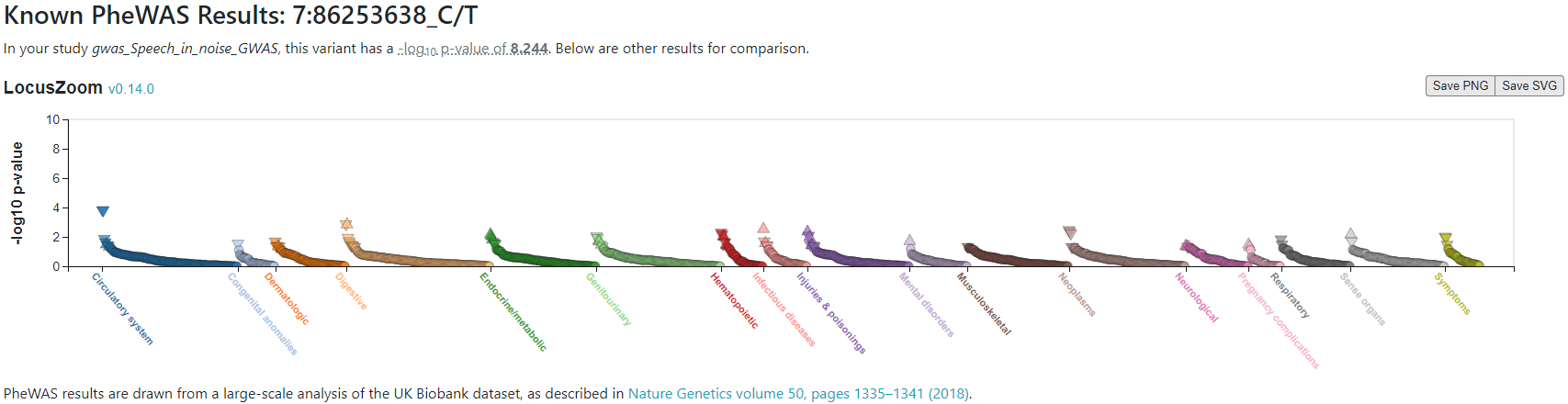


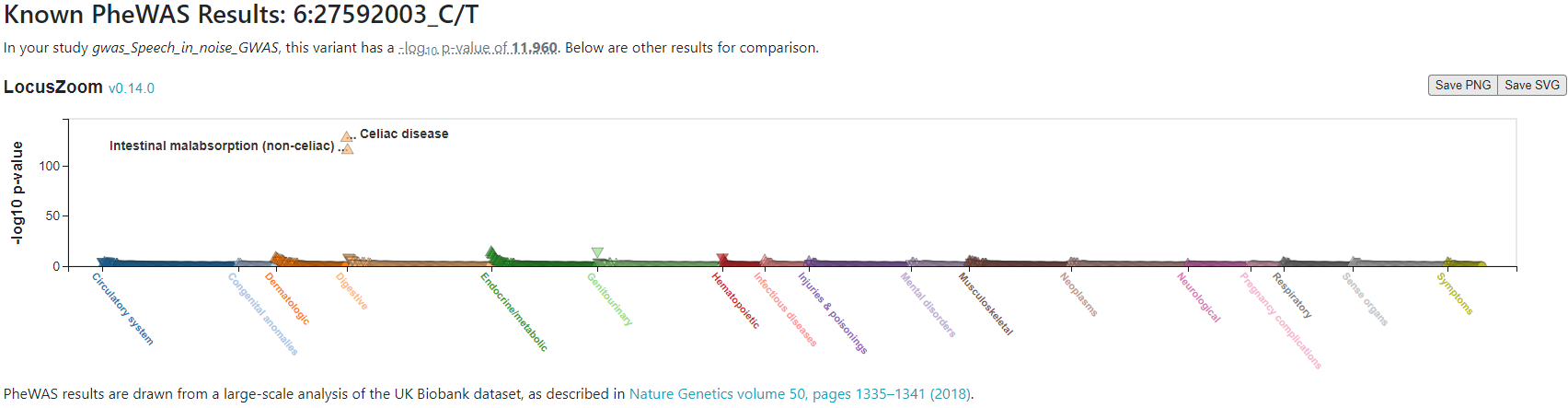


**Figure 8**: Results of the replication analysis. Scatter plots present the effect size estimates derived from the linear mixed model for SSQ12, hearing thresholds, DPOAEs for SNPs included in the replication analysis (with SIN GWAS *p*<10^-6^). The beta values between hearing thresholds and SSQ12 did not reveal a statistically significant association (*p*>0.05). The beta values between SSQ12 and DPOAEs, and hearing thresholds and DPOAEs showed significant negative correlation coefficients (*p*<0.05). The scatter plot allows visualization of the effect size estimates across audiological measures.

**
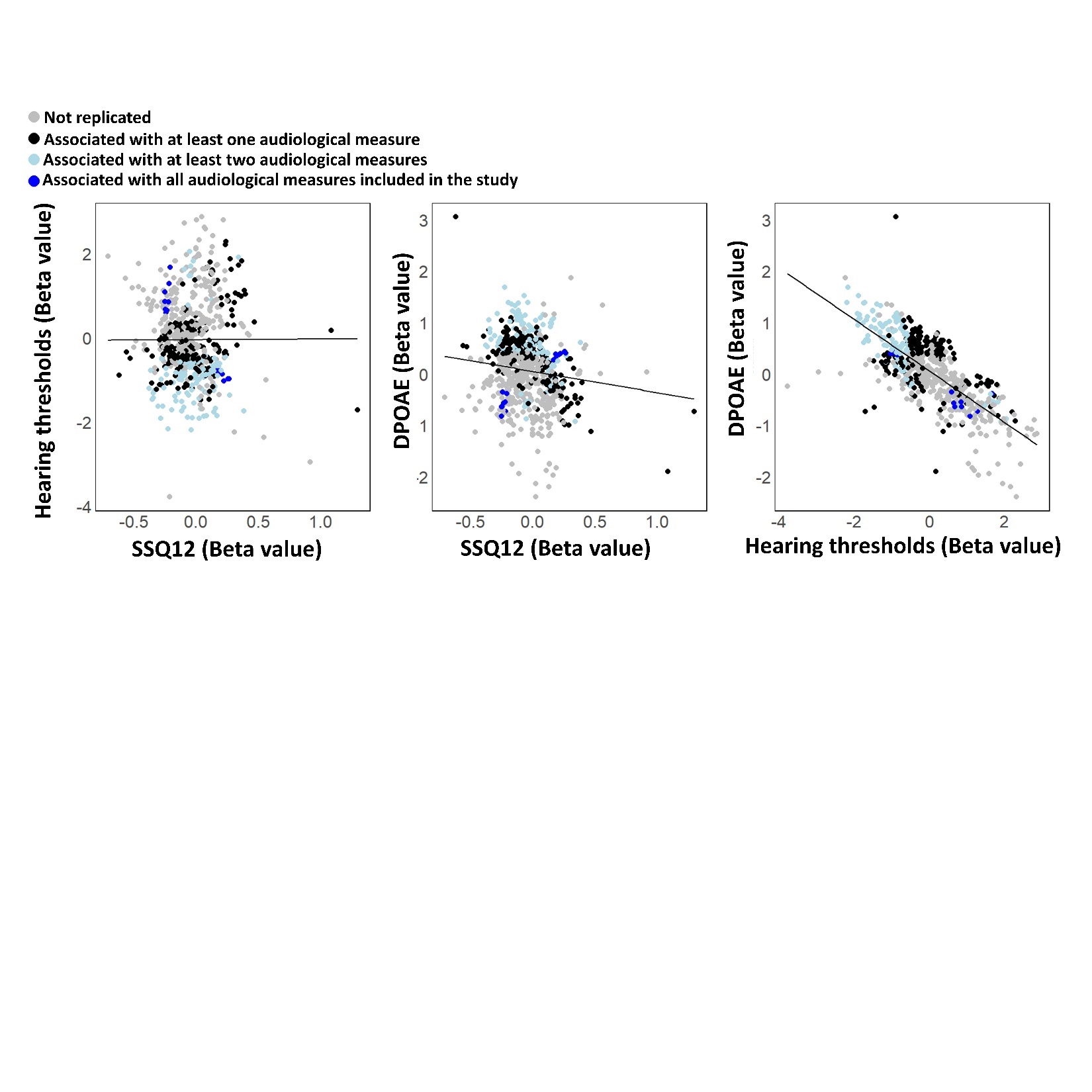
**

**Figure 9**: Hearing thresholds and distortion-product otoacoustic emissions (DPOAEs) between individuals with rs116208485 TT and TC/CC variants. Individuals with TT variants revealed poorer SSQ12 results, and yet, they showed significantly better hearing thresholds and DPOAEs in both ears.


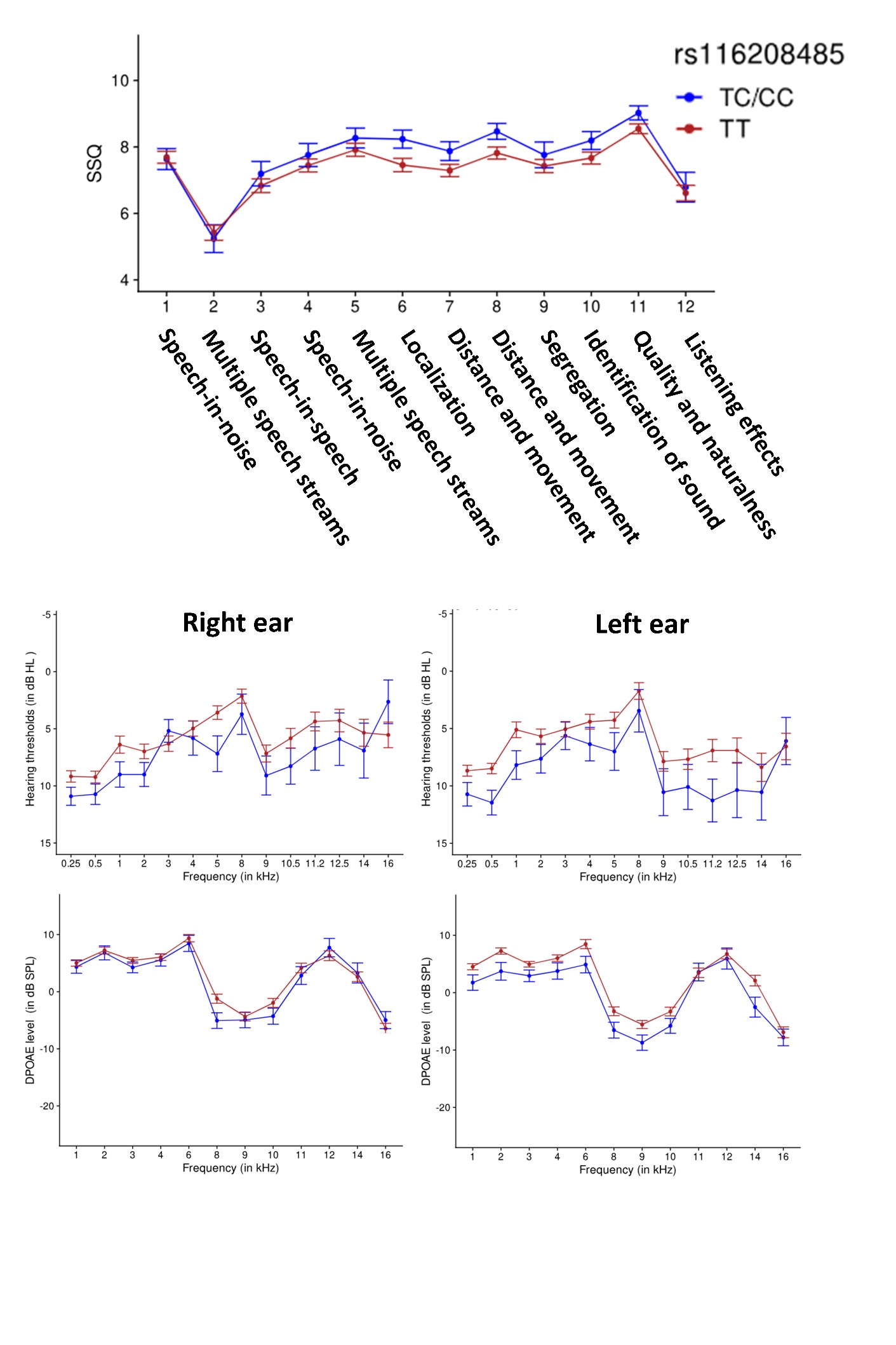


**Figure 10**: The results of the cochlear cell-line enrichment analysis using single-cell transcriptomic data from mice cochlear tissues showing normal hearing thresholds (for details, Boussaty et al., 2023). After the quality control and filtering steps, transcriptomic data from 12805 cells from mice with normal hearing thresholds were used for the analysis.


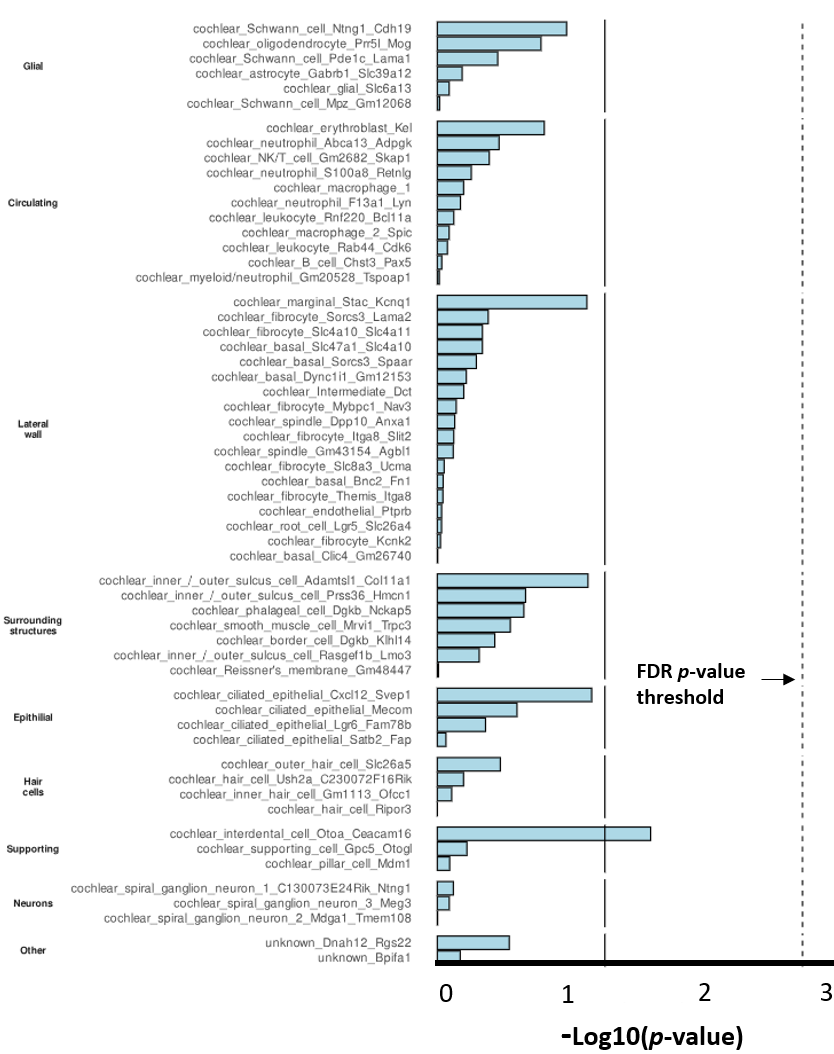

Supplement: Supplementary file 2 — Supplementary Information 2. [file 41598_2024_63972_MOESM2_ESM.docx]
