# Supplementary material for: A genome-wide association study reveals a polygenic architecture of speech-in-noise deficits in individuals with self-reported normal hearing
Source: Sci Rep. 2024 Jun 7;14:13089. doi: 10.1038/s41598-024-63972-2 (PMC11161523; doi:10.1038/s41598-024-63972-2)

**Supplement File S3:** Results of the enrichment analysis for SIN deficits after removing the MHC region (Chr:6, BP:25-34 GB) from the genome-wide association study (GWAS) summary statistics.

**Figure 1:** The GWAS Manhattan plot after removing the HLA region (Chr:6, BP:25-34 GB).


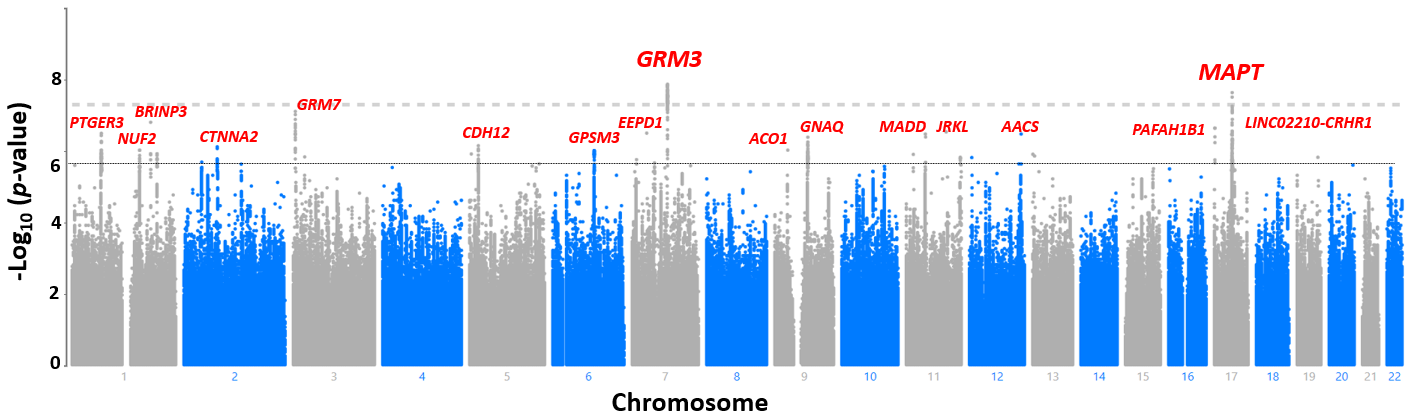


**Figure 2:** Quantile-Quantile (Q-Q) plots of expected and observed *p*-values (converted on a -log10 (*p*-value) scale) for the genome-wide association study of the SIN phenotype after removing the HLA region (Chr:6, BP:25-34 GB). The plot on the right-hand side shows Q-Q plots for minor allele frequency-based categories and genomic inflation measurement (GC lambda).


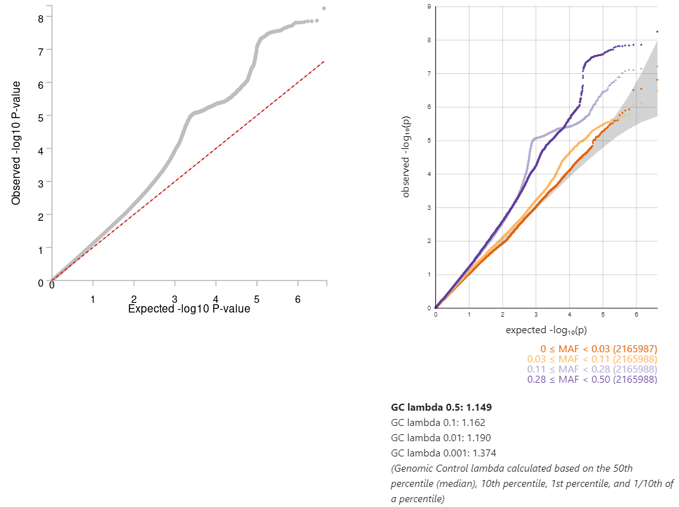


**Figure 2:** Results of the enrichment analysis: the SIN phenotype after removing the HLA region (Chr:6, BP:25-34 GB)

**Figure 2.1**: Top results of the MAGMA gene-set analysis: the SIN phenotype after removing the HLA region (Chr:6, BP:25-34 GB)

| **Gene Set** | **N genes** | **Beta** | **Beta STD** | **SE** | **P** | **Pbon** |
| --- | --- | --- | --- | --- | --- | --- |
| BIOCARTA_TUBBY_PATHWAY | 4 | 2.3974 | 0.0348 | 0.48111 | 3.169E-07 | 0.005 |
| GOBP_POSITIVE_REGULATION_OF_CYCLASE_ACTIVITY | 36 | 0.5808 | 0.02528 | 0.12587 | 1.99E-06 | 0.03 |
| GOBP_ACTIVATION_OF_ADENYLATE_CYCLASE_ACTIVITY | 23 | 0.6783 | 0.0236 | 0.1558 | 6.74E-06 | 0.11 |
| GOBP_POSITIVE_REGULATION_OF_ADENYLATE_CYCLASE_ACTIVITY | 29 | 0.5882 | 0.02298 | 0.13757 | 9.58E-06 | 0.16 |
| GOBP_POSITIVE_REGULATION_OF_LYASE_ACTIVITY | 37 | 0.5034 | 0.02221 | 0.12129 | 1.67E-05 | 0.28 |
| GOBP_REGULATION_OF_ADENYLATE_CYCLASE_ACTIVITY | 48 | 0.4524 | 0.02272 | 0.11279 | 3.04E-05 | 0.51 |
| GOBP_REGULATION_OF_LYASE_ACTIVITY | 60 | 0.4003 | 0.02247 | 0.10184 | 4.26E-05 | 0.72 |
| GOCC_PRESYNAPSE | 531 | 0.1411 | 0.02327 | 0.03783 | 9.60E-05 | 1 |
| KOMMAGANI_TP63_GAMMA_TARGETS | 9 | 0.9531 | 0.02075 | 0.25618 | 9.98E-05 | 1 |
| GOBP_ADENYLATE_CYCLASE_ACTIVATING_G_PROTEIN_COUPLED_RECEPTOR_SIGNALING_PATHWAY | 133 | 0.2802 | 0.02338 | 0.07695 | 0.00013612 | 1 |

**Figure 2.2**: Positional gene-set analysis


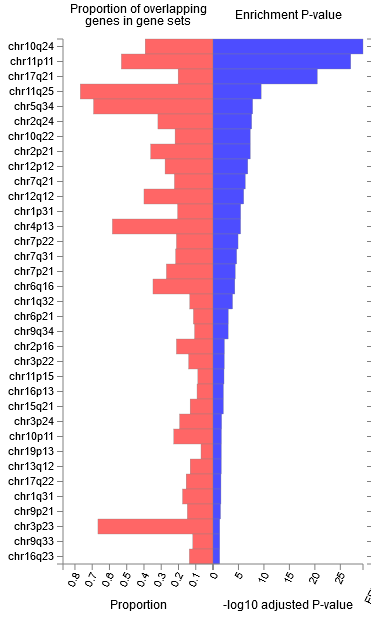


**Figure 2.3**: Curated gene-set analysis


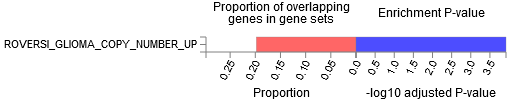


**Figure 2.4**: Chemical and genetic perturbation gene sets (MsigDB c2)


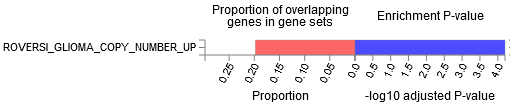


**Figure 2.5**: KEGG gene-set analysis (MsigDB c2)


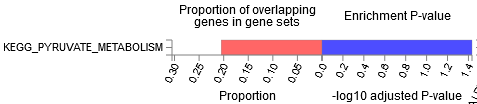


**Figure 2.6**: Reactome (MsigDB c2)


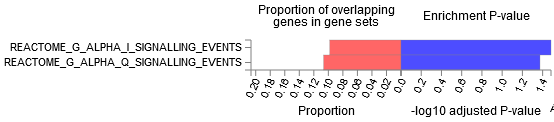


**Figure 2.7**: TF-targets (MsigDB c4)


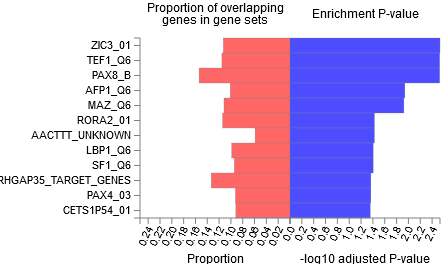


**Figure 2.7**: Cell-type signatures (MsigDB c4)


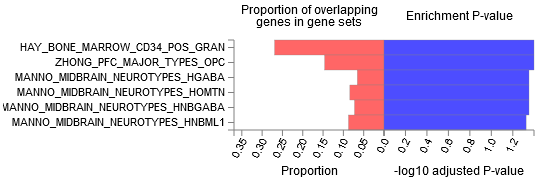


**Figure 2.8:** GWAS catalog reported gene set analysis


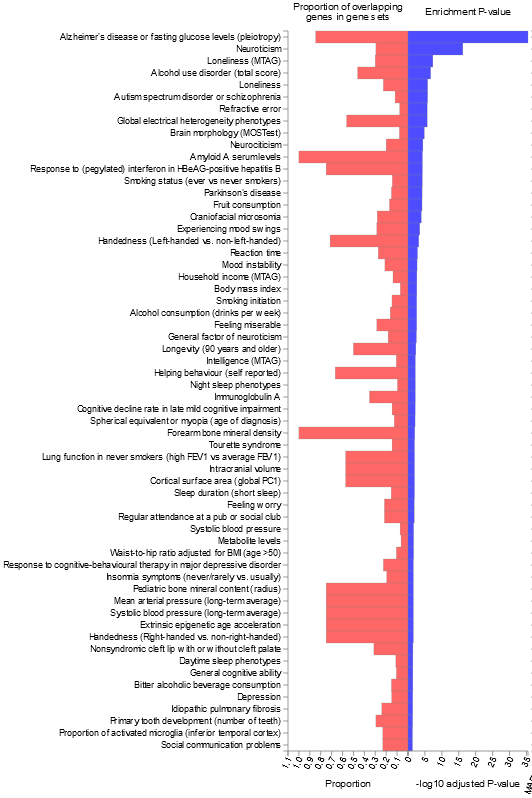


**Figure 2.8:** Results of the GWAS gene set enrichment analysis with FUMA for the SIN phenotypes after removing the HLA region (Chr:6, BP:25-34 GB). This figure presents a subset of the GWAS gene set associations obtained for the SIN phenotype (more details in Supplementary file S2). The phenotype category is delineated with distinct colors (from top-to-bottom, left-to-right): Pink - Cardiovascular; Blue - Inflammatory; Green - Metabolic; Purple - Musculoskeletal; Cyan - Neural; Orange - Pulmonary; Navy Blue - Cognitive; Dark Green - Sensory; Gray - Sleep; Black - Other; Light Orange - Neuropsychiatric; Red - Lifestyle;  The X-axis represents the proportion of overlapping genes, and their colors represent the enrichment p-values. The circle size is determined by the number of genes tested for association.

**
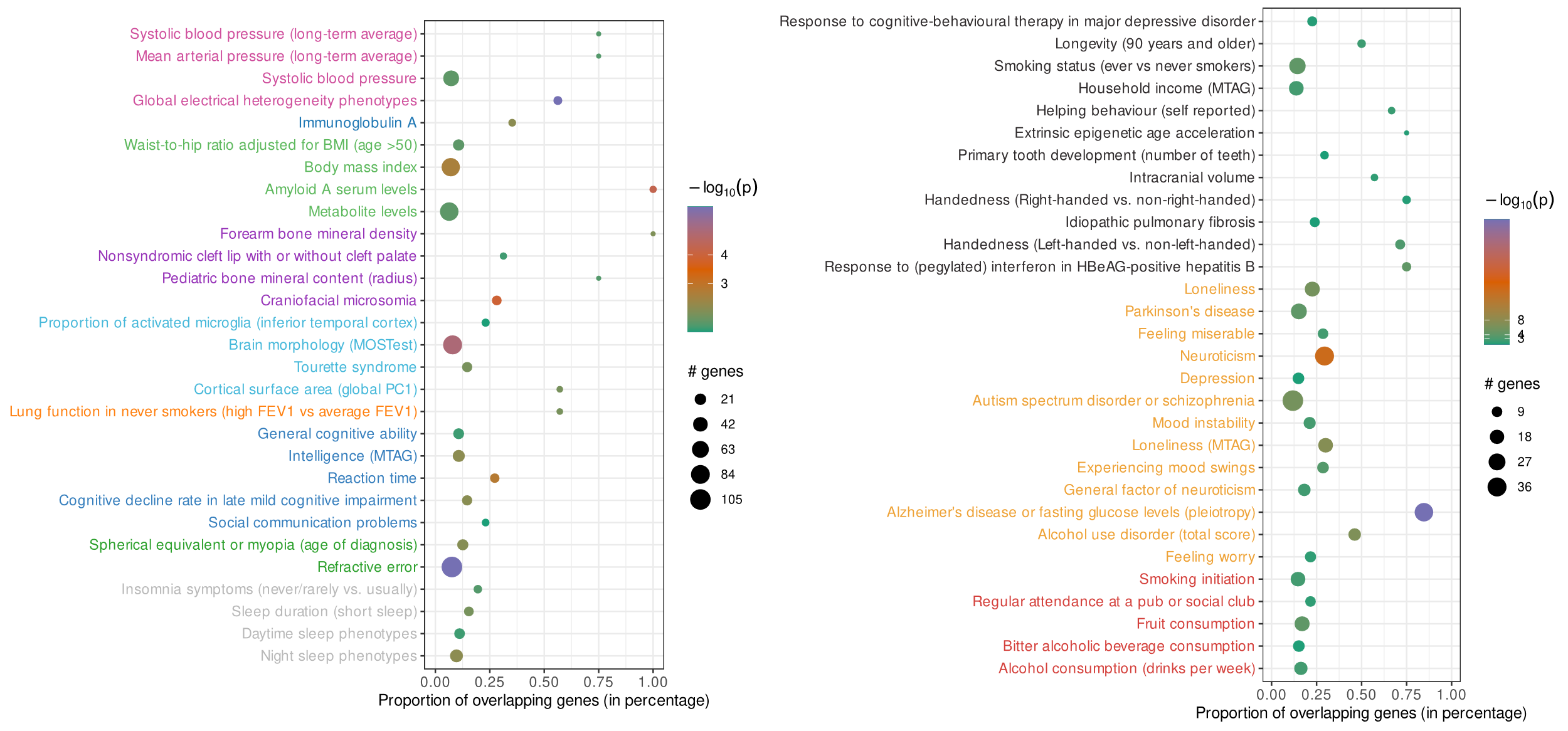
**

**Figure 3:** Manhattan plot: Gene-based test (FUMA) using the GWAS summary statistics after removing the HLA region (Chr:6, BP:25-34 GB).


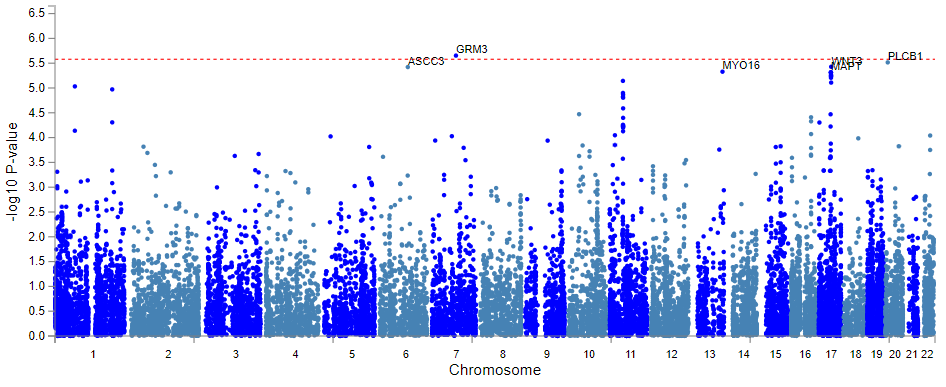


**Figure 4:** MAGMA Tissue expression analysis using the GWAS summary statistics after removing the HLA region (Chr:6, BP:25-34 GB).

**Figure 4.1:** GTEx v8 30 general tissue types


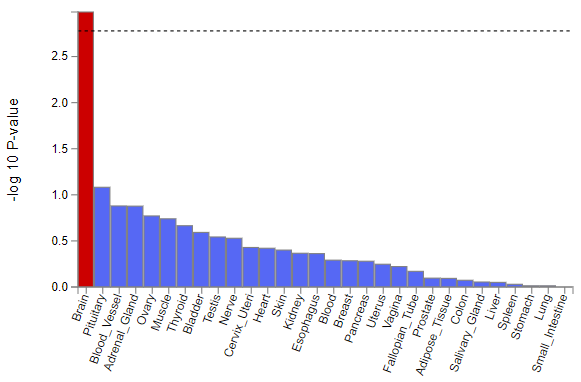


**Figure 4.2:** GTEx v8 53 tissue types


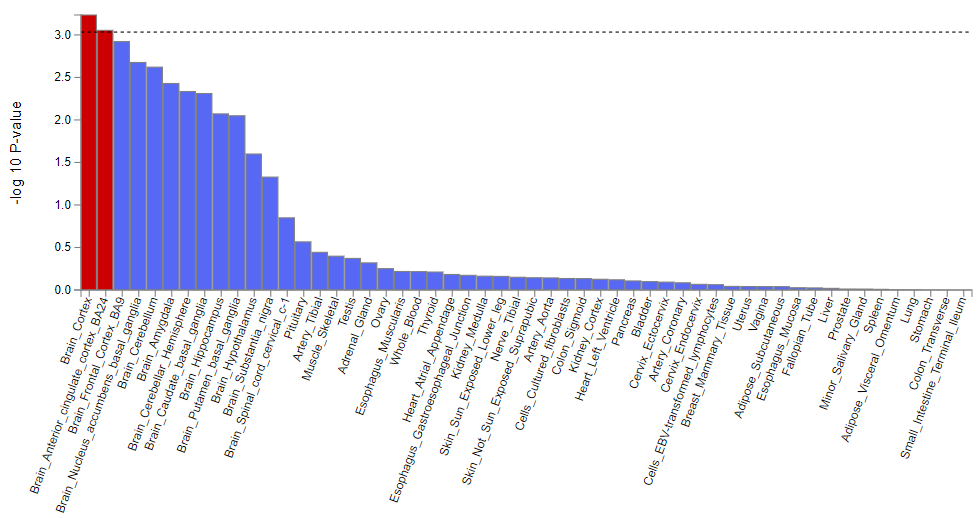


**Figure 4.3:** Cell-type specific gene set enrichment analysis for human prefrontal cortex (GSE104276)


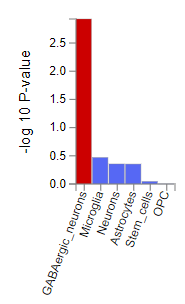


**Figure. 5**: Cell- and tissue-specific partitioning heritability analysis of SIN deficits after removing the HLA region from the GWAS summary statistics. A) Partitioned heritability with LDSC regression shows enrichment in brain-specific regulatory regions of the genome. Tissue-specific regulatory elements are marked by histone 3 acetylation or DNase hypersensitivity (for open chromatin) and H3K4me1 (for enhancers). B) Partitioned heritability-based enrichment analysis identified tissues involved in SIN deficits. C) Brain-specific analysis of partitioned heritability shows distinct regions of the brain associated with SIN genetic architecture. The graph shows *p*-values (in -log10) tissue and cell marker types. The dashed line shows the *p*-value threshold for significant enrichment after FDR correction for the number of gene sets tested.

**
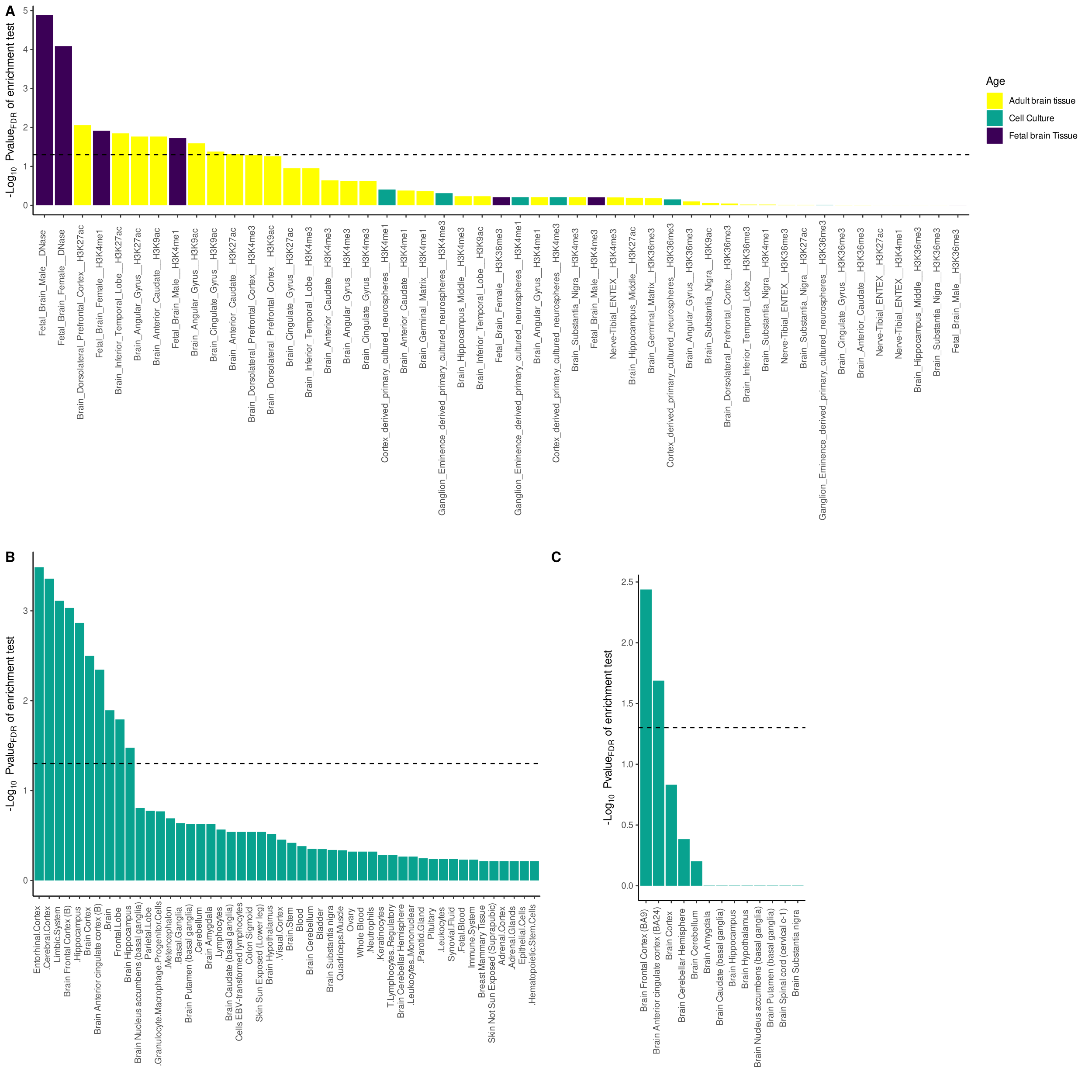
**

**Figure. 6.1**: The results of the cochlear cell-line enrichment analysis using single-cell transcriptomic data from mice cochlear tissues after removing the HLA region from the GWAS summary statistics.


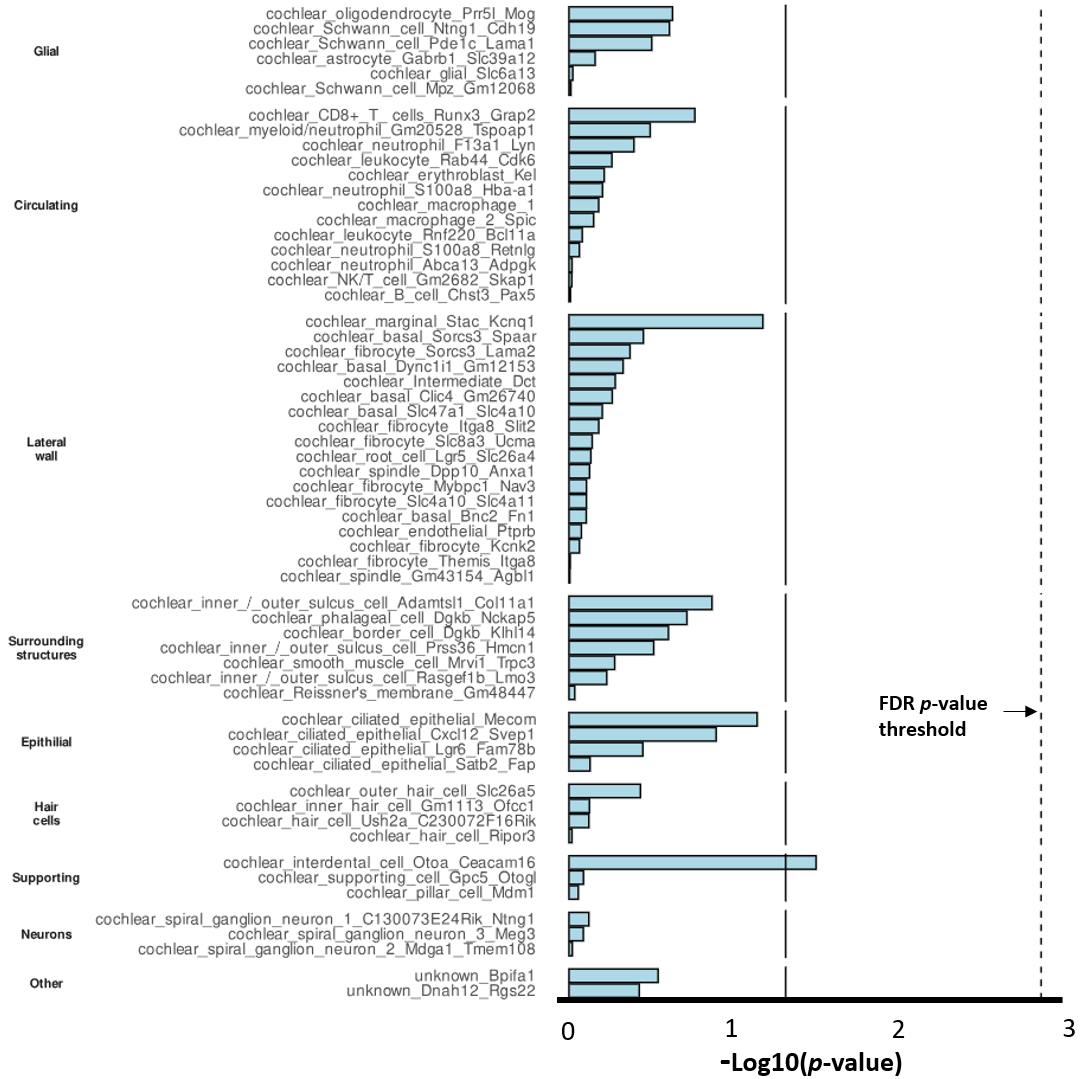


**Figure. 6.2**: The results of the cochlear cell-line enrichment analysis using single-cell transcriptomic data from mice cochlear tissues showing normal hearing thresholds (for details, Boussaty et al., 2023) after removing the HLA region from the GWAS summary statistics. After the quality control and filtering steps, transcriptomic data from 12805 cells from mice with normal hearing thresholds were used for the analysis.


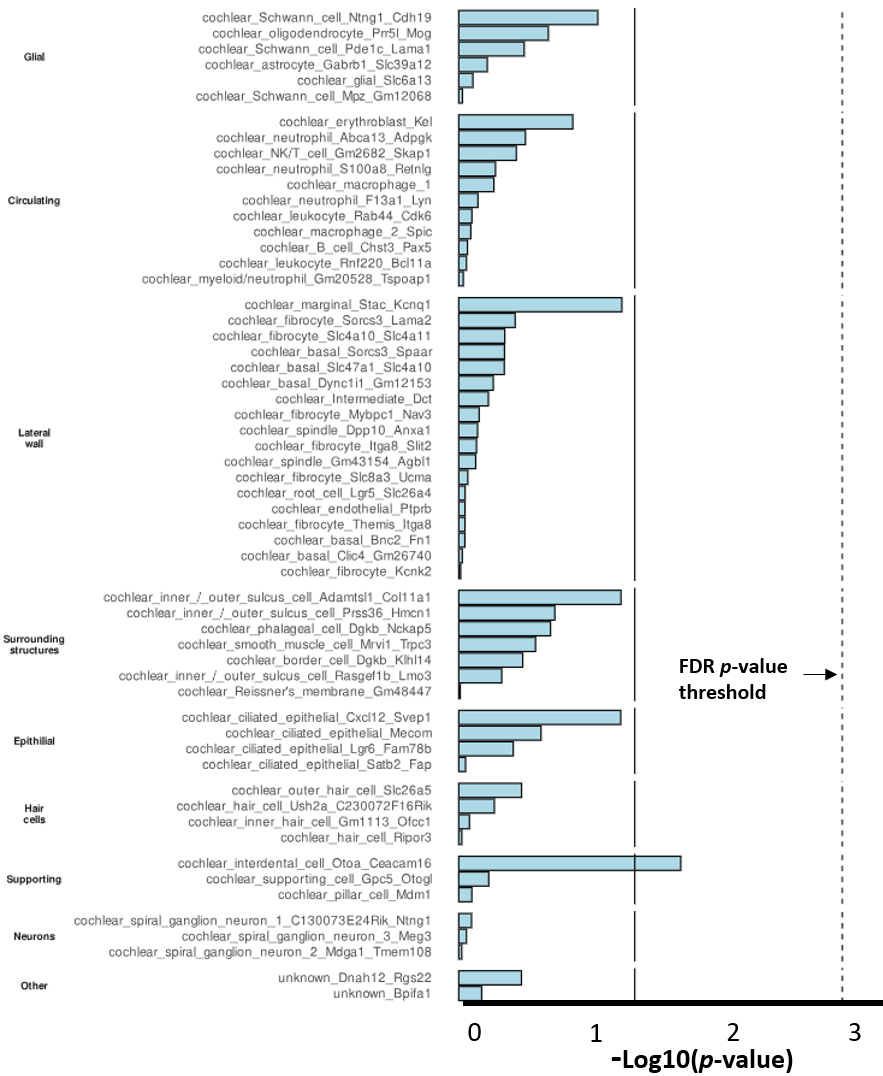

Supplement: Supplementary file 4 — Supplementary Information 4. [file 41598_2024_63972_MOESM4_ESM.docx]
